# Transcription Analysis of Central Metabolism Genes in *Escherichia coli*. Possible Roles of σ^38^ in Their Expression, as a Response to Carbon Limitation

**DOI:** 10.1371/journal.pone.0007466

**Published:** 2009-10-19

**Authors:** Leticia Olvera, Alfredo Mendoza-Vargas, Noemí Flores, Maricela Olvera, Juan Carlos Sigala, Guillermo Gosset, Enrique Morett, Francisco Bolívar

**Affiliations:** Departamento de Ingeniería Celular y Biocatálisis, Instituto de Biotecnología. Universidad Nacional Autónoma de México (UNAM), Cuernavaca Morelos, México; Baylor College of Medicine, United States of America

## Abstract

The phosphoenolpyruvate: carbohydrate transferase system (PTS) transports glucose in *Escherichia coli*. Previous work demonstrated that strains lacking PTS, such as PB11, grow slow on glucose. PB11 has a reduced expression of glycolytic, and upregulates *poxB* and *acs* genes as compared to the parental strain JM101, when growing on glucose. The products of the latter genes are involved in the production of AcetylCoA. Inactivation of *rpoS* that codes for the RNA polymerase σ^38^ subunit, reduces further (50%) growth of PB11, indicating that σ^38^ plays a central role in the expression of central metabolism genes in slowly growing cells. In fact, transcription levels of glycolytic genes is reduced in strain PB11*rpoS* ^−^ as compared to PB11. In this report we studied the role of σ^70^ and σ^38^ in the expression of the complete glycolytic pathway and *poxB* and *acs* genes in certain PTS^−^ strains and their *rpoS* ^−^ derivatives. We determined the transcription start sites (TSSs) and the corresponding promoters, in strains JM101, PB11, its derivative PB12 that recovered its growth capacity, and in their *rpoS^−^* derivatives, by 5′RACE and pyrosequencing. In all these genes the presence of sequences resembling σ^38^ recognition sites allowed the proposition that they could be transcribed by both sigma factors, from overlapping putative promoters that initiate transcription at the same site. Fourteen new TSSs were identified in seventeen genes. Besides, more than 30 putative promoters were proposed and we confirmed ten previously reported. *In vitro* transcription experiments support the functionality of putative dual promoters. Alternatives that could also explain lower transcription levels of the *rpoS* ^−^ derivatives are discussed. We propose that the presence if real, of both σ^70^ and σ^38^ dependent promoters in all glycolytic genes and operons could allow a differential transcription of these central metabolism genes by both sigma subunits as an adaptation response to carbon limitation.

## Introduction

The phospohoenolpyruvate: carbohydrate transferase system (PTS), in conjunction with Crp and Cra, constitute the main regulatory system in *Escherichia coli* involved in catabolite carbon repression that allows the utilization of glucose as the preferred carbon source. This system, composed of several proteins, is the major consumer of phosphoenolpyruvate (PEP) for the coupled translocation and phosphorylation of glucose into glucose-6P. Strains lacking this system (PTS^−^) have a significantly reduced transport rate of glucose and other sugars, resulting in permanent slow growth on glucose when it is the only carbon source [1]–[3]. In wild type *E. coli* strains the presence of PTS makes it difficult to unveil the role of other mechanisms and modulators involved in carbon scavenging responses, such as σ^38^. In fact, in strains lacking *crr* that codes for the PTS EIIA^Glc^ component, translation of the *rpoS* messenger is no longer repressed by EIIA^Glc^, thus allowing higher levels of σ^38^, even during the exponential growth phase [4]. This sigma factor takes over from σ^70^ in the expression of many genes during entry into the stationary phase [5], [6]. In a similar way when wild type cells are grown with glucose as a limiting nutrient, and in PTS^−^ strains that grow slowly on glucose, transcription of several central metabolism and especially glycolytic genes, turns out to be also under the control of σ^38^ [3], [5]–[11]. Thus, it has been proposed that σ^38^ should be considered a second vegetative sigma factor because hundreds of genes are under its control when *E. coli* cells are exposed to stress conditions, including starvation and growing slowly on glucose [5], [7], [11]–[15]. Therefore, these set of genes could have promoters for both sigma factors.

Differential DNA recognition specificities of the various sigma subunits allows promoter selectivity to the RNA polymerase core (RNAp). In *E. coli*, the alarmone ppGpp modulates the binding affinity of different sigma subunits to the RNAp; high ppGpp concentrations favors binding of σ^38^ over σ^70^ [16]–[20]. In addition, there are other factors such as DksA and Crl that apparently modulate the binding of different sigma subunits to the RNAp [6]. σ^70^ and σ^38^ are very similar proteins; in fact, genes whose transcription is σ^70^-dependent *in vivo* can often be transcribed *in vitro* by σ^38^ and *vice versa*. The current view about the “selectivity paradox”, as mentioned by Weber *et al* [5], is that these two sigma factors recognize very similar promoters, and that minor differences at the −10 region shift the specificity towards one or the other RNAp holoenzyme. Moreover, transcription initiation by the RNAp σ^38^ holoenzyme is apparently less affected by deviations from the promoter consensus (e.g. degeneration of the −35 sequence), which allows this holoenzyme to transcribe from “non-optimal” promoters [5], [6], [21]–[23]. In addition, transacting transcriptional regulators, such as Fis, H-NS and IHF, as well as DNA topology, can contribute to promoter selectivity [6], [24]. Remarkably and in support of the results of this contribution, the recent analysis of the σ^32^ regulon in *E. coli* revealed an extensive overlap between targets of this alternative sigma factor and those of σ^70^. Some degree of promoter degeneration apparently allows both RNAp holoenzymes to use overlapping promoters that share transcriptional start sites (TSSs), giving plasticity to the transcription process [6], [25]–[27].

**Figure 1:**
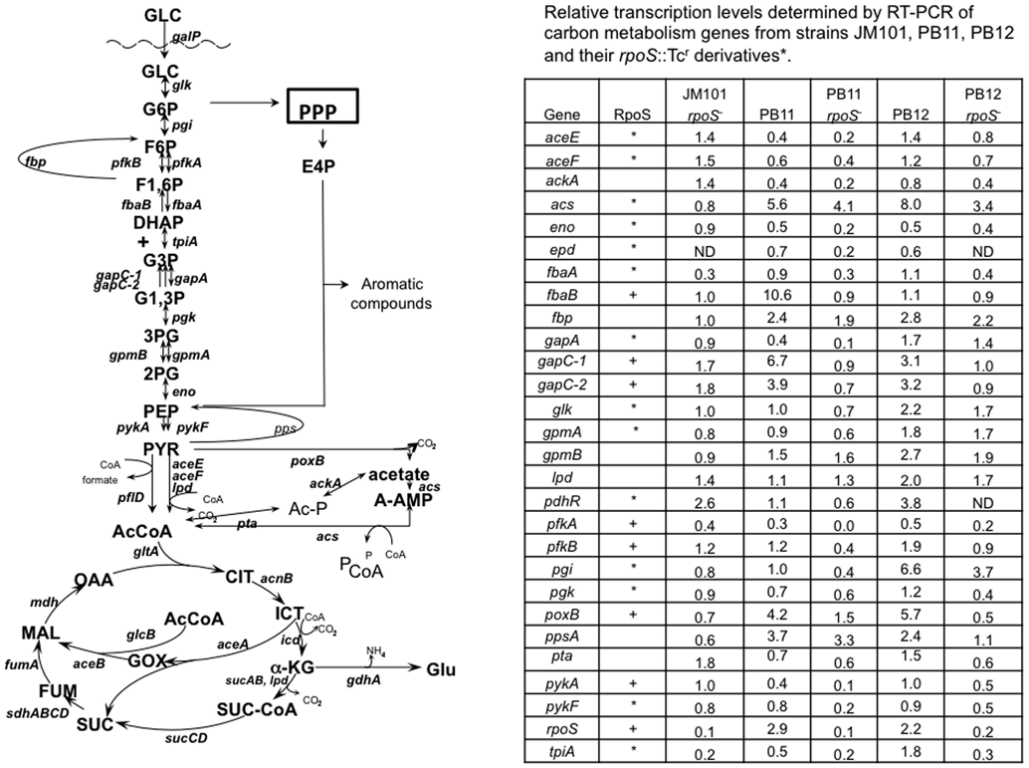
Central metabolic routes in the PTS^−^ strains showing key metabolites and the genes involved in their transformation. PTS, (not shown in the figure) is involved in the wild type strain in glucose transport and phosphorylation, using PEP and producing pyruvate. RTPCR values of these genes in strains JM101, PB11, PB12 and their *rpoS* ^−^ derivatives are shown in the included table. These RTPCR values have been previously reported [3], [11] and are presented in this figure for comparison and discussion purposes. The abbreviations are as follows: glucose (GLC), glucose-6-phosphate (G6P), fructose-6-phosphate (F6P), fructose-1,6-phosphate (F1,6P), dihydroxy-acetone phosphate (DHAP), glyceraldehyde-3-phosphate (G3P), glyceraldehyde-1,3-phosphate (G1,3P), 3-phosphoglycerate (3PG), 2-phosphoglycerate (2PG), phosphoenolpyruvate (PEP), pyruvate (PYR), acetyl-CoA (AcCoA), acetyl phosphate (Ac-P), acetyl-AMP (A-AMP), citrate (CIT), isocitrate (ICT), glyoxalate (Gox), α-ketoglutarate (α-KG), succinyl-coenzyme A (SUC-CoA), succinate (SUC), fumarate (FUM), malate (MAL), oxaloacetate (OAA).

We have been involved in the characterization of *E. coli* strains lacking PTS (PTS^−^), such as strain PB11 and its derivative strain PB12, which was selected for growth rate recovery in a chemostat with glucose fed at progressively faster rates. As expected, carbon flux analyses showed that PB11 has a reduced glycolytic capacity. Interestingly, the adaptive evolution process that gave rise to PB12 rendered an increase in its glycolytic capacity [28], [29]. In agreement with these results, transcriptome studies have shown that several glycolytic genes are downregulated in PB11; in contrast, they are upregulated in PB12, as compared to the parental wild type strain JM101. In these PTS^−^ strains that grow slowly on glucose, *poxB* and *acs* are significantly upregulated [3]. Therefore, it has been proposed that in strains lacking PTS, PoxB (pyruvate oxidase) and Acs (acetylcoenzyme synthase) play an important role in the transformation of pyruvate into acetylcoenzyme (AcCoA). In support of this proposition, *poxB* inactivation not only decreased 50% the specific growth rate (μ) of PB11 but carbon fluxes for the conversion of pyruvate into AcCoA were doubled in PTS^−^ strains as compared to the wild type parental strain JM101 and the expression of some glycolytic genes was downregulated in PB11*poxB* ^−^ as compared to PB11 [30]. Interestingly, in these strains *rpoS*, the gene coding for σ^38^, was also upregulated (figure 1), [3], [29], [30] and its inactivation which reduces the transcription of all glycolytic genes, produced also a dramatic negative effect on the growth rate of PB11. In addition, the expression of *spoT*, *gpp*, *ndk* and *ppa* genes, whose products are involved in ppGpp metabolism, was upregulated in PB12 and *rpoS* inactivation also decreased their transcription, suggesting that σ^38^ has a critical role in the expression of these genes in slow growing *E. coli* cells [11].

In this report, we carried out a detailed molecular characterization of the expression of all glycolytic genes and operons and two other central carbon metabolism genes -*poxB* and *acs*-, in the wild type and in the PTS^−^ strains by determining their TSSs. This information, and the determination of the TSSs of the same set of genes in the *rpoS* ^−^ derivatives of these strains, allowed us to determine the possible type of promoter(s) controlling their expression. Remarkably, all the genes and operons studied apparently could have more than one promoter. For many of them, σ^70^ binding sites and sequences resembling σ^38^ recognition elements (also mentioned as “possible σ^38^ recognition sites”, through this report), were superimposed. The presence of overflapping σ^70^ and possible σ^38^ recognition sites in these genes, allowed the proposition that they could be transcribed by both sigma subunits from “putative dual promoters”, in which transcription initiation, if directed by both sigma factors, occurred at the same site. In some cases, we found differential promoter usage among the studied strains and also between their *rpoS* ^−^ derivatives. The detailed knowledge at the molecular level of the different metabolic strategies for carbon utilization by *E. coli* strains lacking PTS, will allow a better understanding of the physiology of cells growing slowly on glucose. This information can also be useful in the design of novel metabolic engineering strategies for improving carbon diversion into the aromatic pathway utilizing PTS^−^ strains such as PB12 [28], [31], [32]. Finally, the analysis of this information and other related to the regulation of the glycolytic pathway and to the transformation of pyruvate into AcCoA, allowed the clarification of an important carbon diversion metabolic switch regulated by both σ^38^ and pyruvate concentrations that apparently occurs in *E. coli* strains growing slowly on glucose, at the level of the pyruvate dehydrogenase complex (Pdh) and PoxB to preserve carbon skeletons.

## Results

### 1. Transcription analysis of glycolytic genes and operons and two other central metabolism genes in strains JM101, PB11 and PB12

Here we investigated the promoters directing the expression of the glycolytic and pyruvate decarboxylation genes in *E. coli* strains growing aerobically on glucose, as the only carbon source (figure 1). Our previous work as well as that of several other groups has provided evidence for a complex regulation of the majority of these genes by both σ^70^ and σ^38^ [3], [7], [11], [30], [31], [33], [34]. However, the molecular details of this control and the type and disposition of the different promoters are still incomplete. In this work, we utilized a modified 5′RACE methodology and a global genomic strategy by pyrosequencing to determine the TSSs, and subsequently the promoters of all these genes in strains JM101, PB11, PB12 and their *rpoS* ^−^ derivative strains. Both methodologies utilized as template total RNA extracted from the cells (Materials and methods, Mendoza *et al*, submitted to PLoS ONE). In the case of the 5′RACE methodology, we utilized the same RNA that was previously used for quantitative detection of the mRNA of these genes by real time PCR (RTPCR) [3], [11]. For the modified 5′RACE analysis we designed oligonucleotide primers specific for each gene in order to recover from the RNA mixture only those cDNAs of interest, and extended the oligonucleotide primer till the end of the molecule, which normally corresponds to the 5′end of the mRNA. In contrast, the global pyrosequencing strategy does not select for any particular gene and the results obtained normally correspond to the degree of expression of each gene (Mendoza *et al*, submitted to PLoS ONE). For each gene analyzed by the modified 5′RACE we detected on PAGE at least one band corresponding to the gene-specific amplification product. For some genes we detected also nonspecific PCR products, arising from partial complementarity, coming mainly from the highly abundant rRNAs (Mendoza *et al*, submitted to PLoS ONE). In several cases, when the intensities of the bands in the gels were weak, DNA bands for the same size were mixed together and sequenced, resulting in the same TSSs. In Materials and methods and Supporting Information sections (figure S1 and table S1), the procedures utilized for cDNA synthesis, separation and sequencing are described in detail. Even when the modified 5′RACE methodology is not quantitative, in several cases the DNA band intensities in the gels correlated with previously reported RTPCR expression values (figure 1) [3], [11], probably because the same RNA was utilized for both experiments; although there were several exceptions. The cDNA bands in the gels were extracted and sequenced up to their 5′ end to allow TSSs determinations. Putative σ^70^ and σ^38^ recognition sequences (table 1) were identified by visual inspection of the DNA sequences upstream the TSSs and by motif finding with the MEME and meta-MEME programs (Materials and methods). The consensus nucleotide sequence of the −10 region for σ^38^, described by Weber *et al* [5], was utilized in this work for detecting possible σ^38^ recognition sites. Because the −35 region is less conserved or even absent, especially in the σ^38^ promoters [5], [6], [21]–[23], only the −10 region was considered in our analyses. When putative superimposed promoters for both σ^38^ and σ^70^ were detected sharing the same TSS, an additional number was included in the promoter name to indicate this fact. In those cases we named as mentioned, these two putative overlapping promoters as “putative dual promoters”. *In vitro* transcription experiments and DNA fusions of various promoters to a reporter gene of some of these genes were obtained as additional evidence to support the functionality of these promoters. In the next subsections, the analysis of transcription results for all the glycolytic, *poxB* and *acs* genes is presented:

**Table 1:** Transcriptional start sites for analyzed genes and their possible −10 elements.

name	promoter	references	involved TFs	data mapping (TSS and −10 element)	ATG dist.	sigma scores^1^	inferred boxes	Additional evidence
*glk*	*glk*P1^k^-P2^n^	[36], in this work	FruR	GCGTTGTTGTTATGCCCCCAGGTATTTACAGTGTGAGAA	−36	s70: 3.92	s38^2^	C
	*glk*P3^n^-P4^n^	in this work		CCCCCAGGTATTTACAGTGTGAGAAAGAATT ATTTTGAC	−21	s38: 2.95	s70^3^	A,C
*pgi*	*pgi*P1^k^-P2^n^	[37], in this work		ACCATCACATTTTTCTGTGACTGGCGCTACAATCTTCCA	−36	s70: 6.9; σ38: 7.44	-	A,B,E
*pfkA*	*pfkA*P1^k^-P2^n^	[39], in this work	FruR	ATCAATTCAGCAGGAAGTGATTGTTATACTATTTGCACA	−78	s70: 8.61; σ38: 5.7	-	A,B
	*pfkA*P3^n^-P4^n^	in this work		TCACTTCGATGTGCAAGAAGACTTCCGGCAACAGATTTC	−29	s70: 1.12	s38^2^	A,E
*tpiA*	*tpiA*P^ki^	[40]		CTGCCCTGCGGGGCGGCCATCTTCCTTTATTCGCTTATA	−19	-	-	-
	*tpiA*P1^n^-P2^n^	in this work		CAAAGCCTTTGTGCCGATGAATCTCTATACTGTTTCACA	−62	s70: 8.07; σ38: 7.4	-	A,E
*gapA*	*gapA*P1^k^-P1a^n^	[41], [43], in this work		CGCTTGACGCTGCGTAAGGTTTTTGTAATTTTACAGGCA	−36	s70: 3.76; σ38: 0.82	-	A,B,D
	*gapA*P2^k^	[41], [43]		CCTTTAAAATTCGGGGCGCCGACCCCATGTGGTCTCAAG	−153	-	-	A
	*gapA*P3^k^	[41], [43]	Crp	TCACATTTTTATCGTAATTGCCCTTTAAAATTCGGGGCG	−174	-	-	-
	*gapA*P4^k^	[41], [43]		AACACCAACTGGCAAAATTTTGTCCTAAACTTGATCTCG	−245	-	-	-
*epd*	epdP1^k^-P2	[45], in this work	FruR, Crp	ACATTCCTTTTATTCCACGTTTCGCTTATCCTAGCTGAA	−132	no predictions	s70^3^,σ38^2^	A,D
*pgk*	*pgk*P1^k^	[45]		AAGCAGCACAAGGTGCATTTCATGGTATAGTTGACTATA	−235	-	-	A
	*pgk*P2^n^	in this work		TGTTGCTTTCAGGTAAGACGCAAGCAGCGTCTGCAAAAC	−26	no predictions	-	D,E
	*pgk*P3^n^	in this work		GCACACCTGATCAAAACGTTGGTCTGGTGCGATAACGAA	−113	no predictions	s70^3^	A,D
*gpmA*	*gpmA*P1^ki^-P2^n^	[46], in this work	Fur	AAGCATTGCTGTTGCTTCGTCGCGGCAATATAATGAGAA	−37	s70:7.56; σ38: 3.66	-	A
	*gpmA*P3^n^-P4^n^	in this work		CCTTACACTGCGCCACTATTTTCGCTATGGTTATGCGTA	−75	s70:5.80; σ38: 7.48	-	-
*eno*	*eno*P1^k^	[47]		AATCGTGTCAGCGTCAACATCAAACTGATCGATTCACAA	−731	-	-	-
	*eno*P2^k^	[47]		CGAAGCACTGAAACACGGTGGGCTGAAGAATCGTGTCAG	−760	-	-	-
	*eno*P3^k^	[47]		TAAACGATTCAGCTTAAACTGCCCGGAAGCGAATCTGTC	−903	-	-	-
	*eno*P4^n^-P5	in this work		GCGTACCCTGGGTACGCGTTGTTTGT***CTGGAGTTTCAGT***	−27	no predictions	s70^3^,σ38^2^	A,D,E
	*eno*P6^n^	in this work		GAGTTCCAGAAACGTCAGGCGAAGTAAGTAAAAAAGTTA	−77	no predictions	-	D
	*eno*P7^n^-P8^n^	in this work		GGGGATGATCAGTTGGTCGAGATCATCGAAGTTCCGAAT	−212	no predictions	s70^3^,σ38^2^	D
*pykF*	pykFPΠκι	[49]	FruR	TGTTTTCCTTTTGGATTAATTTCAGCGTATAATGCGCGC	−231	-	-	-
	*pykF*P1^n^-P2^n^	in this work		GGATTCGCTTTCCGGCAGTGCGCCCAGAAAGCAAGTTTC	−34	no predictions	s70^3^,σ38^2^	A,B,D
	*pykF*P3^n^	in this work		GATGTCACCTATCCTTAGAGCGAGGCACCACCACTTTCG	−80	no predictions	-	D
	*pykF*P4^n^-P5^n^	in this work		CCTCTGCACGCTTTTTCGATGTCACCTATCCTTAGAGCG	−97	s70: 4.79; σ38: 9.95	-	B,D
*pykA*	*pykA*P1^n^-P2^n^	in this work		AAGTGACGATCGCTAAAAACGACTGTCACTGTCCTAATC	−105	s70: 0.90	s38^2^	B,D
	*pykA*P3^n^-P4^n^	in this work		GTCAAAGAAGCGCTGAAGGAATCGCGTTTTGATAAGCAG	−153	s70: 1.76	s38^2^	A,B,D
*pdhR*	*pdhR*P1^k^-P2^n^	[51]–[52], in this work	Crp, Fnr, PdhR	GTATGGACATAAGGTGAATACTTTGTTACTTTAGCGTCA	−58	no predictions	s70^3^,σ38^2^	B,D
*aceE*	aceEP1^k^-P2^n^	[50], in this work		AACCTGTCTTATTGAGCTTTCCGGCGAGAGTTCAATGGG	−48	s70: 1.90	s38^2^	-
*poxB*	*poxB*P1^k^-P2^n^	[57], [58], in his work	MarA, SoxS	TCCCTTCCCCCTCCGTCAGATGAACTAAACTTGTTACCG	−27	s70: 5.31; σ38: 9.5	-	B,C
*acs*	*acs*P1^k^	[60]–[61]	Fis, Crp, Ihf	TCTTTAATCAATTGTAAGTGCATGTAAAATACCACTTTA	−224	-	-	
	*acs*P2^k^-P3^n^	[60]–[61], in this work	Fis, Crp, Ihf	CCCCTACATTTAACGCTTATGCCACATATTATTAACATC	−20	s70: 4.03; σ38: 4.97	-	B,C
			consensus sequences	sigma 70:TGGTATAATG sigma 38:CTACACTT				

Proposed promoters in which putative σ^70^ and σ^38^ overlapping recognition sequences have been determined, are presented as bold sequences.

The transcription start site (TSS) for each gene is underlined at the end of each nucleotide sequence. (1) predictions were obtain with PWMs (σ^70^ and σ^38^) analyzed in meta-MEME software and inferred (2) by visual comparison using the −10 sigma 38 consensus sequence by Weber, H. et al. [5]. (3) in these cases, σ^70^ promoter sequences were inferred using the −10 sigma 70 consensus sequence. (n) new data obtained in this work; (k)known data; (ki) previously inferred promoters not detected in this work. Additional evidence that supports the functionality of the proposed promoters detected by 5′RACE: (A) global mapping of TSS; (B) *in vitro* transcription; (C) obtained with two tags; (D) repeated experiments and (E) promoter fusions to a reporter gene. TFs, transcriptional factors.

### A) Transcription start site mapping and promoter analysis of glycolytic genes and operons

In this section, the determination of TSSs and putative promoters for each glycolytic gene in JM101, PB11, PB12, and their *rpoS* ^−^ derivative strains using 5′RACE analysis, is presented.

#### a) *glk*

**Figure 2:**
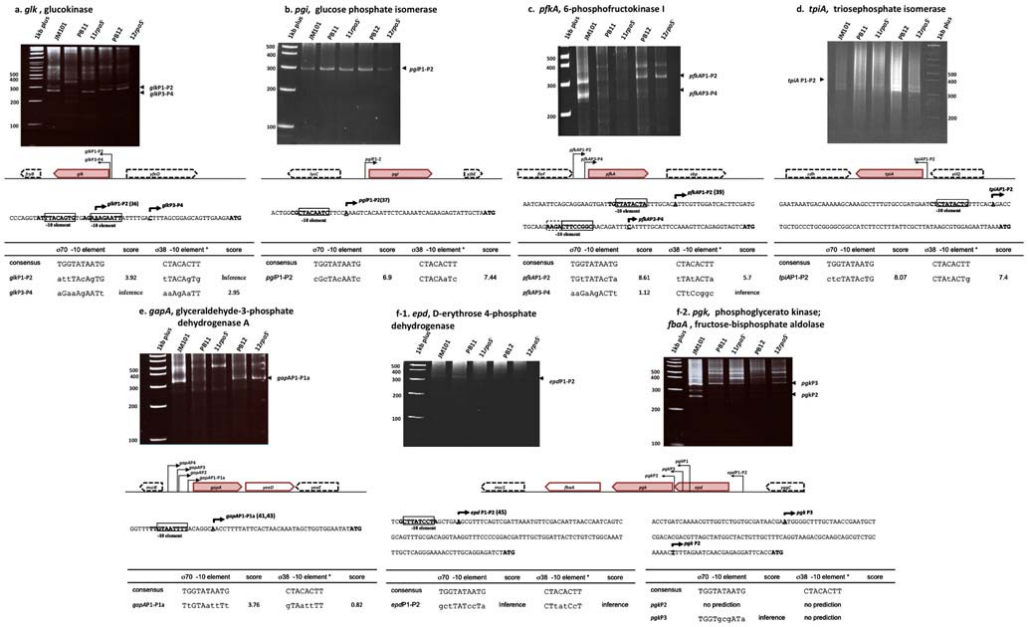
Transcription start sites (TSSs) of the *glk*, *pgi*, *pfkA*, *tpiA*, *gapA*, *epd* and *pgk* glycolytic genes and operons were determined using a modified 5′RACE methodology, as described in materials and methods. Specific oligonucleotides were designed for each gene in order to recover from the RNA mixture the cDNAs of interest. The cDNAs in the bands were extracted and their nucleotide sequences were determined to their 5′ends to allow TSSs determinations. When multiple PCR bands were present in one gel, these bands were purified and their nucleotide sequences were determined. In some cases, ribosomal or tRNA were detected. In most cases, no specific DNA sequences were obtained indicating nonspecific amplified PCR products (see a precise explanation in the text for each subfigure; the nucleotide sequences of the different cDNA are presented in figure S1 and table 1). The consensus nucleotide sequence of the −10 region for the σ^38^ sigma subunit (8 nucleotides, framed in the figures) described by Weber *et al* [5], was utilized for detecting possible σ^38^ recognition sites and a 10 nucleotides −10 σ^70^ consensus sequence was utilized for detecting possible σ^70^ binding sites. Numbers in parenthesis indicate references in which previously reported TSSs have been described: Meyer *et al* [36], Froman *et al* [37], Helling and Evans [39], Charpentier *et al* [41], Thouvenot *et al* [43], Bardey *et al* [45].

This gene codes for glucokinase (Glk), the enzyme that phosphorylates glucose into glucose-6P in the absence of PTS (figure 1) [29], [35]. A σ^70^-dependent promoter has previously been reported for this gene [36]. As shown in figures 2a and S1a, there are multiple bands in the gel in which *glk* TSSs were detected. Only the two smaller ones between 200 and 300 bp produced *glk* complementary sequences. Nucleotide sequence of the longest of these bands (shown in the gel as *glk*P1-P2), detected only in PB12 and its *rpoS* ^−^ derivative PB12*rpoS* ^−^ strains, showed that transcription initiated at an A residue, 36 nucleotides upstream the ATG initiation codon. This TSS was one nucleotide longer than a previously reported one [36]. We named this putative dual promoter *glk*P1-P2 because a sequence resembling a σ^38^ recognition element overlaps the σ^70^ recognition element (see below). Interestingly, JM101, PB11 and PB11*rpoS* ^−^ strains utilized a different downstream possible dual promoter, *glk*P3-P4. Nucleotide sequence of the pooled bands from these strains showed that transcription started at a C residue, 21 nucleotides from the ATG initial codon. The *glk*P3-P4 putative dual promoter does not have a clear σ^70^ recognition element but its transcription is apparently dependent on this sigma factor since in the *rpoS* ^−^ strains it was still expressed. Sequences resembling σ^38^ recognition sites were detected in both *glk*P1-P2 and *glk*P3-P4 putative dual promoters (figures 2a and S1a, table 1). Remarkably, inactivation of *rpoS* in PB11*rpo*S^−^ strain apparently enhanced in the gel the amount of transcript from *glk*P3-P4. RTPCR expression values (figure 1) indicated that in PB11 and in PB12 strains transcription of *glk* was only slightly dependent on σ^38^ been in the limits of the experimental error; nevertheless, transcription of these genes, detected by RTPCR was downregulated (figure 1) [3], [11].

#### b) *pgi*

This gene codes for phosphoglucose isomerase (Pgi) an enzyme involved in the transformation of glucose-6P into fructose-6P (figure 1). A σ^70^-dependent promoter, which directs transcription starting at an A residue, 36 nucleotides upstream the ATG initiation site has been reported [37]. As shown in figures 2b and S1b, after 5′RACE was performed a single band was detected in all strains. Nucleotide sequences of these bands showed that all initiated at exactly the same position as reported. In PB11 and PB12 strains there was clearly a higher amount of this transcript than in JM101, which is in agreement with the previously reported phosphoglucose isomerase specific activity in PB12 that was 4–5 fold higher than in JM101 (figure 1) [3], [11], [29]. Less amount of transcript was detected in the gel in the *rpoS* ^−^ derivatives as compared to their respective *rpoS* ^+^ strains, in agreement with reported RTPCR expression values. A possible strong σ^38^ recognition -10 element overlapping the σ^70^ recognition element was located in the promoter region of this gene (figures 2b and S1b, table 1). This result allowed the proposition that *pgi*, as it will be seen for other genes below, could be transcribed by these two sigma factors from possible overlapping σ^70^ and σ^38^ promoters. Therefore, we named it *pgi*P1-P2 putative dual promoter to indicate that the same TSS could be shared by two different RNAp holoenzymes.

#### c) *pfkA*

The main phosphofructokinase in *E. coli* is coded by the *pfk*A gene (figure 1). *pfkA* mutants are growth impaired on glucose. However, there is a second minor (10%) phosphofructokinase activity, the product of the *pfkB* gene. This explains the residual growth of *pfkA* mutants [38]. A σ^70^-dependent promoter has been reported which directs transcription for the *pfkA* gene initiating at an A residue, 78 nucleotides from the ATG start codon [39]. As shown in figures 2c and S1c, three main bands appeared in the gel in all strains after 5′RACE methodology was performed. The sequence of the uppermost band corresponded to rRNA. The nucleotide sequences of the middle bands, clearly observed in JM101, PB12, and PB12*rpoS* ^−^ showed that in all strains transcription initiated at the same position as previously reported [39]. We named this putative dual promoter *pfkA*P1-P2 (see below). Very low intensity bands located at the same position in strains PB11 and PB11*rpoS* ^−^ suggests that this promoter was utilized in all strains. The shorter band, which is clearly observed in strain JM101, produced a sequence that started at a C residue, 29 nucleotides from the ATG start codon. Since there were equivalent bands in the other strains it is likely that all of them initiated at this position, although much less efficiently. This low level of expression is in agreement with reported RTPCR transcription values (figure 1) [3], [11]. The latter transcription event was the result of a second *pfkA*P3-P4 possible dual promoter (see below). Interestingly, inactivation of *rpoS* in PB11*rpo*S^−^ decreased transcription from both putative dual promoters (*pfkA*P1-P2 and *pfkA*P4-P5), indicating that these promoters could be recognized by σ^38^ and could be dual promoters. In fact, a highly consensus possible σ^38^ −10 element was located upstream the TSS of the *pfkA*P1-P2 dual promoter, and another possible σ^38^ recognition −10 element that included the σ^70^ −10 element, could be also located in the *pfkA*P3-P4 putative dual promoter; however, in this case the possible σ^38^ binding site is located two nucleotides closer to the ATG initiation site (figures 2c and S1c, table 1). Again, the level of transcription of this gene was higher in PB12, as expected from RTPCR expression values (figure 1), and in this strain *rpoS* inactivation apparently decreased transcription mainly from the *pfkA*P3-P4 promoter, despite having a poor putative σ^38^ recognition sequence.

#### d) *tpiA*

*tpiA* codes for triosephosphate isomerase (TpiA). This enzyme catalyzes the transformation of dihydroxyacetone-P into glyceraldehyde-3P (figure 1). As shown in figures 2d and S1d, there were two main distinct bands in the gel but only the smallest one, labeled in the gel as *tpiA*P1-P2, and present in all the strains, came from *tpiA*. The other band produced an 23S rRNA sequence. Nucleotide sequences of bands from strains JM101, PB11, and PB12 showed the same TSS, not previously reported that initiated at an A residue, 62 nucleotides upstream the ATG start codon. This newly identified putative dual promoter *tpiA*P1-P2, carried strong overlapping possible σ^70^ and σ^38^ recognition sequences (figures 2d and S1d, table 1). Pichersky *et al* [40] predicted a promoter much closer to the ATG that was not detected in our work. Due to the low transcription levels of this gene in the PB11 strain, it was difficult to unambiguously conclude about the effect of *rpoS* inactivation on PB11*rpoS* ^−^ because of the presence of very small amounts of DNA in the gels. However, less DNA was apparently present in the *rpoS* ^−^ derivatives as compared to their parental *rpoS* ^+^ strains, in agreement with RTPCR values (figure 1), indicating that transcription of this promoter could be σ^38^-dependent.

#### e) *gapA*

Four promoters have been reported for the *gapA* gene, which codes for glyceraldehyde-3-phosphate dehydrogenase A (GapA) (table 1, figure 1), and transcription of one of them (*gapA*P3) is regulated by Crp [41]. 5′RACE experiments resulted in three main bands in the gel (figures 2e and S1e). Attempts to determine the nucleotide sequence of the uppermost did not produce any result, and the smallest band produced a DNA sequence that corresponded to 23S rRNA. The middle one labeled in the gel as *gapA*P1-P1a, which was clearly present in all strains except in PB11*rpoS* ^−^, produced sequences pointing to the same TSS that initiated at an A residue, 36 nucleotides from the ATG start codon (figures 2e and S1e). This TSS precisely coincided with the *gapA*P1 reported promoter [41]–[43]. Interestingly, inactivation of *rpoS* decreased transcription in strain PB11*rpo*S^−^, while it remained constant in strain PB12*rpo*S^−^ as compared to PB12. These results coincide with published RTPCR expression values (figure 1) [11]. Again, as in the cases of *pgi* and *tpiA*, in this DNA region a sequence resembling a σ^38^ −10 element, which included the σ^70^ −10 reported element, could explain RTPCR results obtained after inactivation of *rpoS*. Due to previously reported promoters in this gene, this putative dual promoter was labeled as *gapA*P1-P1a.

#### f) *pgk and fbaA*

*pgk* and *fbaA* are part of the *epd-pgk-fbaA* operon in *E. coli* which is conserved in the gamma proteobacteria, as detected by the GeCont Server [44]. *pgk* codes for the phosphoglycerate kinase (Pgk) and *fbaA* codes for the fructose-1,6 biphosphate aldolase (FbaA) [38] (figure 1). Two promoters have been reported for this operon; one that transcribes the three genes, located upstream of *epd*, which initiates transcription 132 nucleotides from the ATG initiation codon and a second one, located at the 3′ end of the *epd* structural gene that transcribes only *pgk* and *fbaA*. This latter promoter initiates transcription 235 nucleotides from the ATG initiation codon of the *pgk* gene [45]. As can be seen in figures 2f-1 and S1f-1 transcription from the *epd* gene initiated in all strains from the same reported promoter whose expression is controlled by Crp and Cra [45]. Interestingly, a sequence resembling a σ^38^ −10 element was detected overlapping this putative dual *epd*P1-P2 promoter (see below), (table 1).

We also investigated the presence of internal promoters between *pgk* and *fbaA* because there is a large intergenic region of 214 nucleotides between these two genes. Oligonucleotide primers specific for *fbaA* were used, but no transcription products were detected, (data not shown), suggesting that there is no promoter immediately upstream of this gene. This result suggests that transcription of *fbaA* is dependent on the promoters located further upstream in the operon, at least in the growth conditions tested. In order to analyze the possible existence of additional TSSs in front of *pgk* we designed specific oligonucleotide primers for this gene. Figures 2f-2 and S1f-2, show multiple bands in the gel. The first and third bands from the bottom of the gel label as *pgk*P2 and *pgk*P3, were the only ones that rendered sequences from this *E. coli* region. The other bands did not produce any sequence in multiple attempts utilizing the *pgk* oligonucleotide primer. The mapped 5′ends of the specific *pgk* sequences were located at 26 (*pgk*P2) and 113 nucleotides (*pgk*P3) from the ATG start codon. A poor sigma σ^70^ recognition site was inferred in the *pgk*P3, but no sequences resembling σ^38^ −10 elements were detected in these two proposed promoters (figures 2f-2 and S1f-2, table 1). We did not detect a previously reported TSS located at 235 nucleotides from the ATG [45]. This could be due to different growth conditions used here. Published RTPCR values indicate that transcription levels of these three genes are lower in PB11 and PB12, as compared to JM101. Inactivation of *rpoS* clearly decreased transcription of *epd*, *pgk* and *fbaA* in the *rpoS* ^−^ derivative strains (figure 1). However, we did not detect any relevant difference in the transcription levels in the gels in the *rpoS* ^−^ derivatives as compared to the corresponding *rpoS* ^+^ strains.

#### g) *gpmA*

**Figure 3 pone-0007466-g003:**
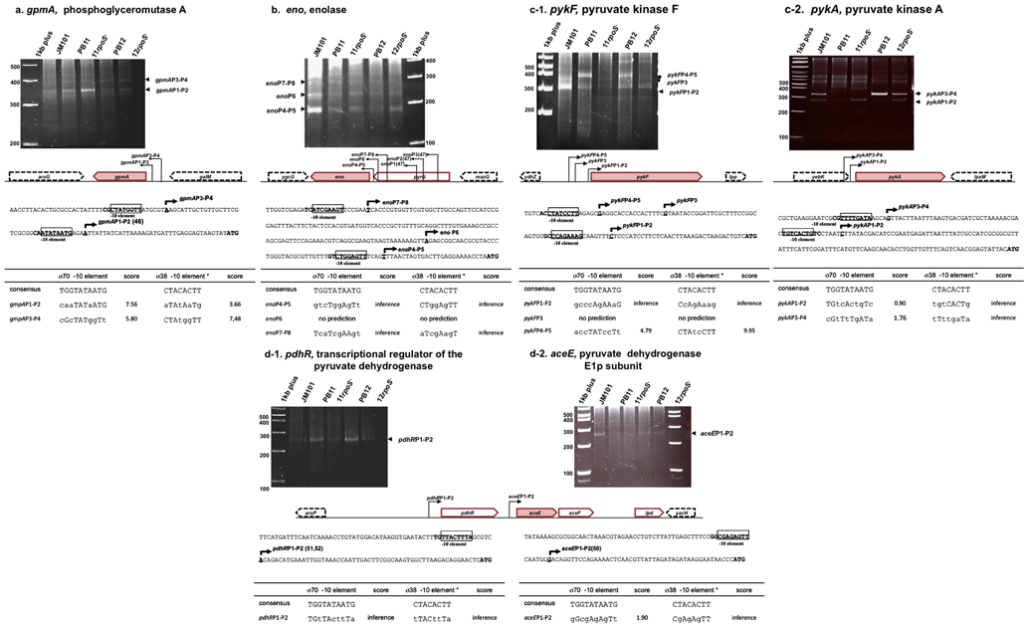
Transcription start sites (TSSs) of the *gpmA*, *eno*, *pykA*, *pykF*, *pdhR* and *aceE* glycolytic genes and operons were determined using a modified 5′RACE methodology, as described in materials and methods. The cDNAs in the bands were extracted and their nucleotide sequences were determined to their 5′ends to allow TSSs determinations. When multiple PCR bands were present in one gel, these bands were purified and their nucleotide sequences were determined. In some cases, ribosomal or tRNA were detected. In most cases, no specific DNA sequences were obtained indicating nonspecific amplified PCR products (see a precise explanation in the text for each subfigure; the nucleotide sequences of the different cDNA are presented in figure S1 and table 1). The consensus nucleotide sequence of the −10 region for the σ^38^ sigma subunit (8 nucleotides, framed in the figures) described by Weber *et al* [5], was utilized for detecting possible σ^38^ recognition sites and a 10 nucleotides −10 σ^70^ consensus sequence was utilized for detecting possible σ^70^ binding sites. Numbers in parenthesis indicate references in which previously reported TSSs have been described: Vassinova *et al* [46], Shimada *et al* [47], Spencer and Guest [50], Quail *et al* [51], Cunningham *et al* [52].

Phosphoglyceromutase A (GpmA) is coded by *gpmA*. 5′RACE experiments resulted in three main bands in the gel (figure 3a). Attempts to sequence the uppermost band did not produce any result. The lower band, labeled in the gel as *gpmA*P1-P2, which was clearly present in all strains, produced nucleotide sequences with the same TSS that initiated at an A residue, 37 nucleotides from the ATG start codon (figures 3a and S1g, table 1). This TSS almost coincided (two nucleotides difference), with a proposed TSS from an inferred σ^70^ promoter for this gene [46]. The middle band present in all strains labeled as *gpm*P3-P4, produced sequences with the same TSS in all strains that initiated transcription at an A residue, 75 nucleotides from the ATG start codon (figure 3a). Interestingly, inactivation of *rpoS* reduced transcription from this band, in strain PB11*rpoS* ^−^ as compared to PB11, in agreement with RTPCR values (figure 1), while there was no apparent effect in PB12*rpoS* ^−^ as compared to PB12 (figure 1, figure 3a). Possible strong σ^38^ recognition elements were located in both *gpmA*P1-P2 and *gpmA*P2-P3 putative dual promoters (figures 3a and S1g, table 1).

#### h) *eno*

Enolase (Eno) is coded by the *eno* gene (figure 1), which is part of the *pyrG-eno* operon. Figures 3b and S1h show the results of the 5′RACE methodology that consistently produced (four independent experiments), three different extension products labeled in the gel *eno*P4-P5, *eno*P6, and *eno*P7-P8. Transcription from the closest and stronger possible dual promoter (*eno*P4-P5) (see below), initiated at a T residue, located 27 nucleotides from the ATG initiation codon. The second (*eno*P6) promoter and the third (*eno*P7-P8) putative dual promoter (see below), initiated transcription at a T and A residues, located 77 and 212 nucleotides, respectively, from the ATG start codon. Strains PB11 and PB12 utilized the same promoters. Inactivation of *rpoS* in strain *PB11rpoS* ^−^ slightly decreased transcription from *eno*P7-P8, in agreement with lower transcription levels detected by RTPCR (figure 1). Sequences resembling poor σ^38^ −10 element were located in the *eno*P7-P8 and *eno*P4-P5 putative dual promoters; however, as shown in the gel, inactivation of *rpoS* apparently did not decrease transcription from this promoter. Shimada *et al* [47], reported three TSSs for this gene with very long 5′ untranslated regions initiating at 731, 760, and 903 nucleotides from the ATG. These TSSs were obtained from cells growing in Luria broth, and were not detected in our work. Importantly, no possible σ^38^ recognition sequences were detected in the promoters reported by Shimada *et al* [47], (table 1).

#### i) *pykF* and *pykA*

*E. coli* has isoenzymes to perform some critical metabolic steps. The pyruvate kinases PykA and PykF are one example (figure 1). It has been shown that PykF plays a more significant role than PykA when *E. coli* is growing aerobically on glucose [48]. Certainly, the conversion of PEP into pyruvate is a very important step that in principle should be finely controlled. Furthermore, in strains lacking PTS, as PB11 and PB12, the pyruvate kinase catalyzed carbon flux from PEP to pyruvate is highly increased probably to compensate for the absence of PTS, the major pyruvate producer [1], [29]. As shown in figures 3c-1 and S1i-1, 5′RACE produced three clear bands in the gel that were the result of promoters not previously reported named *pykF*P1-P2, *pykF*P3, and *pykF*P4-P5, respectively. Transcription from the putative dual *pykF*P1-P2 promoter that started at a C residue located at 34 nucleotides from the ATG, was high in JM101 and was apparently under σ^70^ and σ^38^ control (see below). *pykF*P3 started transcription at a G residue at 80 residues from the ATG and seemed to be specific for JM101 since no bands corresponding to this transcript were detected in the PTS^−^ derivatives. On the contrary, *pykF*P4-P5, which initiated also at a G residue 97 nucleotides from the ATG, was only detected in these mutant derivatives and also seemed to be highly controlled by σ^38^ (see below). Apparently, in these PTS^−^ strains, there was no transcription from *pykF*P3. These results suggest that in the PTS^−^ strains the utilization of the putative dual promoter *pykF*P4-P5 is probably inhibiting the use of the closely located *pykF*P3. A previously predicted promoter (*pykF*P) was not detected in our work [49].

Inactivation of *rpoS* clearly decreased the level of *pykF* transcription detected by RTPCR expression experiments in both PB11*rpoS* ^−^ and PB12*rpoS* ^−^ derivatives (figure 1) [3], [11], in strong agreement with the apparent dependence of the *pykF*P4-P5 promoter on σ^38^, suggesting that this putative dual promoter could be transcribed mainly by this sigma factor but also by σ^70^. This interpretation is supported by the presence of a sequence resembling a strong σ^38^ −10 recognition element upstream this TSS that also includes a possible superimposed σ^70^ −10 element in this putative dual promoter. Interestingly, there was also apparently less transcription as detected in the gel in PTS^−^ *rpoS* ^−^ strains from the *pykF*P1-P2 putative dual promoter as compared to their parental strains. A possible poor σ^38^ −10 recognition element is also present in this putative dual *pykF*P1-P2 promoter (figures 3c-1 and S1i-1, table 1). In fact, less transcription from *pykF*P1-P2 was apparently also observed as compared to the one obtained from *pykF*P3 in strain JM101*rpoS* ^−^ as compared to JM101 (data not shown). This latter result is in agreement with RTPCR values for this gene, where less transcription was detected in JM101*rpoS* ^−^ as compared to JM101 (figure 1) [11]. These results suggest that *pykF*P3 is not apparently regulated by σ^38^ while *pykF*P1-P2 could be transcribed by both sigma subunits. Nevertheless, no clear possible σ^70^ recognition sites were detected neither in *pykF*P1-P2 nor in *pykF*P3.

As shown in figures 3c-2 and S1i-2, two main bands and some nonspecific bands of high molecular weight appeared in the transcription analysis of *pykA*. However, only the two main bands labeled in the gel as *pykA*P1-P2 and *pykA*P3-P4, produced *pykA* specific sequences. Putative dual promoter *pykA*P1-P2 was clearly expressed in JM101 and in the *rpoS* ^−^ derivatives of the PTS^−^ strains indicating that it was apparently transcribed by σ^70^. Interestingly, this transcript that initiated at a C residue, 105 nucleotides from the ATG start codon was apparently as detected in the gel, less expressed in PB11 and PB12, suggesting that σ^38^ could also be modulating its expression. *pykA*P3-P4 that initiated transcription at a G residue, located 153 nucleotides from the ATG start codon, could also be a dual promoter because its expression is diminished in the *rpoS* ^−^ derivatives as compared to the *rpoS* ^+^ parental strains, in agreement with previously reported reduced transcription levels of *pykA* by RTPCR expression experiments (figure 1). However, very poor possible σ^38^ recognition elements are present in these putative dual promoters (figures 3c-2 and S1i-2, table 1). As it will be discussed later, the differential expression of the *pykF* and *pykA* genes, could be the result of different regulatory mechanisms absent in the PTS^−^ strains as compared to JM101.

#### j) The *pdhR-aceEF-lpd* operon

This operon codes for the pyruvate dehydrogenase (Pdh) subunits and its transcriptional regulator PdhR. Pdh is involved in the synthesis of AcCoA from pyruvate (figure 1) [38]. Three different promoters for this operon have been previously described [50]–[52]. Interestingly, two of these promoters are within the operon at 48 nucleotides upstream the *aceE* ATG start codon and 195 nucleotides from the *lpd* ATG initiation codon. This latter promoter is regulated by ArcA since Lpd is also part of the succinate dehydrogenase complex. Therefore, regulation of this operon is very complex [3], [52]–[54].

### *pdhR*

As shown in figures 3d-1 and S1j-1, when the transcripts upstream of *pdhR* were analyzed we found a single band in all strains. The nucleotide sequence of the DNA generated by 5′RACE extracted from every strain pointed to a TSS that coincided precisely with a previously reported one [51], [52]. This TSS initiated at an A residue 58 nucleotides from the *pdhR* ATG start codon and we named this putative dual promoter *pdhR*P1-P2 (see below). Inspection of this promoter region showed possible poor σ^70^ and σ^38^ recognition elements and it could be partially transcribed by σ^38^ since the intensities of the bands in the gel in the *rpoS* ^−^ derivatives were lower than in the *rpoS* ^+^ parental strains. These data are in agreement with RTPCR expression, because transcription values in strain PB11*rpoS* ^−^ are lower as compared to PB11 (figure 1). In agreement with this observation, Weber *et al* [5], reported that this gene is regulated by σ^38^ and it is overexpressed in several stress conditions.

### *aceE*

Since there are reported internal promoters for the *pdhR-aceEF-lpd* operon we decided to investigate possible TSSs in front of the *aceE* gene. AceE is the E1p component of the pyruvate dehydrogenase complex. This subunit binds the thiamin cofactor. Figures 3d-2 and S1j-2 show a main band labeled *aceE*P1-P2 in the gel that was present in all strains (the very faint bands in the uppermost part of the gel are nonspecific). The nucleotide sequence of the band from the JM101 strain indicated that the TSS initiated at a G residue, 48 nucleotides from the ATG start codon of the *aceE* gene. This TSS coincided (one nucleotide difference) with a previously reported one for this gene [50]. According with RTPCR expression values, the transcripts of PB11, PB11*rpoS* ^−^, and PB12*rpoS* ^−^ were very low (figure 1). These results indicate that transcription of this dual putative *aceEF* promoter could be under the control of σ^38^ and, in fact, as in previous cases, a DNA sequence resembling a poor σ^38^ recognition −10 element that includes the reported σ^70^ promoter was detected in this DNA region (figures 3d-2 and S1j-2, table 1).

### B. Transcription start sites determination of *poxB* and *acs* genes by 5′RACE

Pyruvate oxidase (PoxB) and Acetyl CoA synthase (Acs) constitute an alternative metabolic route to produce acetyl CoA from pyruvate by first oxidizing this metabolite to acetate, producing reducing power at the membrane, and then converting acetate to AcCoA (figure 1). Inactivation of *poxB* reduces only 5% the growth rate of the wild type strain, but a 50% reduction is observed in the PB11 strain, indicating the critical role that PoxB plays in cells growing slowly on glucose [3], [30], [55]. In addition, it is known that when *E. coli* cells are growing in low acetate concentrations (< = 10 mM), *acs* inactivation drastically inhibits their growth [56]. Low acetate concentrations produced by PoxB are probably present in PTS^−^ *E. coli* cells growing slowly on glucose and this explains upregulation of *acs* (5–8×) as compared to the wild type, while the expression of the *ackA* and *pta* coding for the other acetate producing/incorporating system, is unchanged [3], [11].

#### a) *poxB*

**Figure 4:**
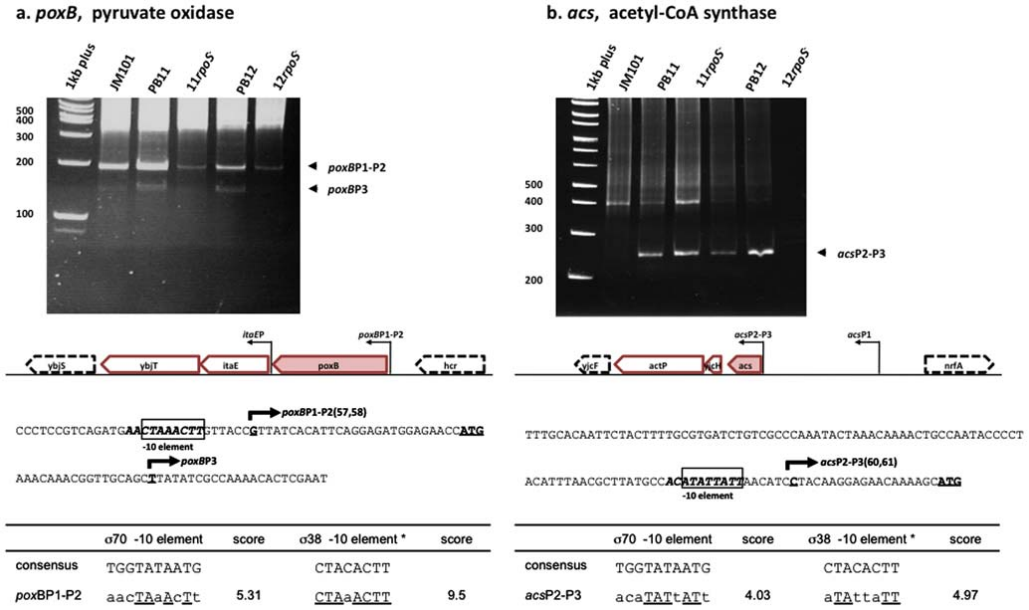
Transcription start sites (TSSs) of the *poxB* and *acs* genes were determined using a modified 5′RACE methodology, as described in materials and methods. The cDNAs in the bands were extracted and their nucleotide sequences determined to their 5′ends to allow TSSs determinations. The nucleotide sequences of the different cDNA, are presented in figure S1 and table 1. The consensus nucleotide sequence of the −10 region for the σ^38^ sigma subunit (8 nucleotides framed in the figures) described by Weber *et al* [5], was utilized for detecting possible σ^38^ recognition sites and a 10 nucleotides −10 σ^70^ consensus sequence was utilized for detecting possible σ^70^ binding sites. Numbers in parenthesis indicate references in which previously reported TSSs have been described: Wise *et al* [57], Chang *et al* [58], Kumari *et al* [60], and Beatty *et al* [61].

This gene forms part of an operon with *itaE* and *ybjT*, which is conserved in the Enterobacteria. Figures 4a and S1k show the amplified PCR products obtained with an oligonucleotide specific for *poxB*. A major band, labeled *poxB*P1-P2 in the gel, was clearly observed in all strains. Sequence analysis showed that the TSSs initiated at a G residue, 27 nucleotides upstream the ATG start site of this gene; the same TSS was previously reported [57]. As expected from previous expression levels detected by RTPCR (figure 1), in the *rpoS* ^−^ derivatives of the PTS^−^ strains (figure 4a), these transcripts were less abundant, which indicate a strong dependence of the *poxB* gene on σ^38^. However, there was still some transcription occurring in the *rpoS* ^−^ strains. As shown above for many glycolytic genes, the expression was also apparently partially dependent on σ^70^. The DNA region upstream of this TSS has a consensus sequence for σ^38^, which has been previously reported [57], [58], as well as an overlapping σ^70^ −10 recognition element that was identified in this study. Therefore, we named this putative dual promoter *poxB*P1-P2. A second band of lower intensity labeled *poxB*P3 in the gel, was detected mainly in strains PB11 and PB12. Nucleotide sequence of this shorter band located this TSS into the *poxB* structural gene, 20 nucleotides downstream the ATG initiation codon. We do not know the functionality of this promoter.

#### b) *acs*

This gene is the first of an operon with *yjcH* and *actP*, present in *E. coli* [59] and several other Enterobacteria, as detected by the GeCont Server [44]. *yjcH* codes for a protein of unknown function, while *actP*, interestingly, codes for an acetate permease. Figures 4b and S1l, show the product of the cDNA amplification as a mayor band in the PTS^−^ strains, labeled as *acs*P2-P3 in the gel. As observed for other analyzed genes, there were high molecular weight nonspecific bands that did not produce DNA sequence. In strains PB11 and PB12, the TSSs determined from the bands obtained from the gel, initiated at a C residue, located 20 nucleotides from the ATG start codon. Interestingly, Kumari *et al* [60], inferred two TSSs based on the presence of binding sites for the regulatory Fnr and CRP proteins. The shorter one coincides precisely with the one mapped here. The second longer one could be one of the bands detected in the uppermost part of the gel. However, as we mentioned above, the DNA bands that appeared in the gels mainly in strains JM101 and PB11 at approximately 400 nucleotides were carefully analyzed but no DNA sequence was obtained. Therefore, we cannot confirm the existence of the other predicted promoter. Beatty *et al* [61], constructed mutants of the putative promoters and analyzed their effect *in vivo* by promoter fusions and by *in vitro* transcription experiments. They concluded that both promoters are functional and the closest one, the same detected here, is the one responsible for the major part of *acs* expression. It is very interesting that this promoter as expected, was highly expressed in the PTS^−^ strains as compared with JM101. We have also observed in RTPCR expression experiments a 5 to 8 times enhanced transcription of the *acs* gene in the PTS^−^ strains (figure 1). These observations are consistent with the expected catabolite repression of acetate utilization in the wild type when growing on glucose [60]. Since the expression of this gene as detected by RTPCR (figure 1), was clearly reduced in the PTS^−^ *rpoS* ^−^ strains as compared to the parental PTS^−^ strains, we classified this *acs*P2-P3 also as a putative dual promoter. Sequence upstream of the TSS showed clear overlapping strong possible recognition sites for both σ^38^ and σ^70^ (figures 4b and S1l, table 1).

### C. Confirmation of several of the detected TSSs by 5′RACE in a global TSS mapping experiment and additional evidence that supports the functionality of the proposed promoters

**Figure 5:**
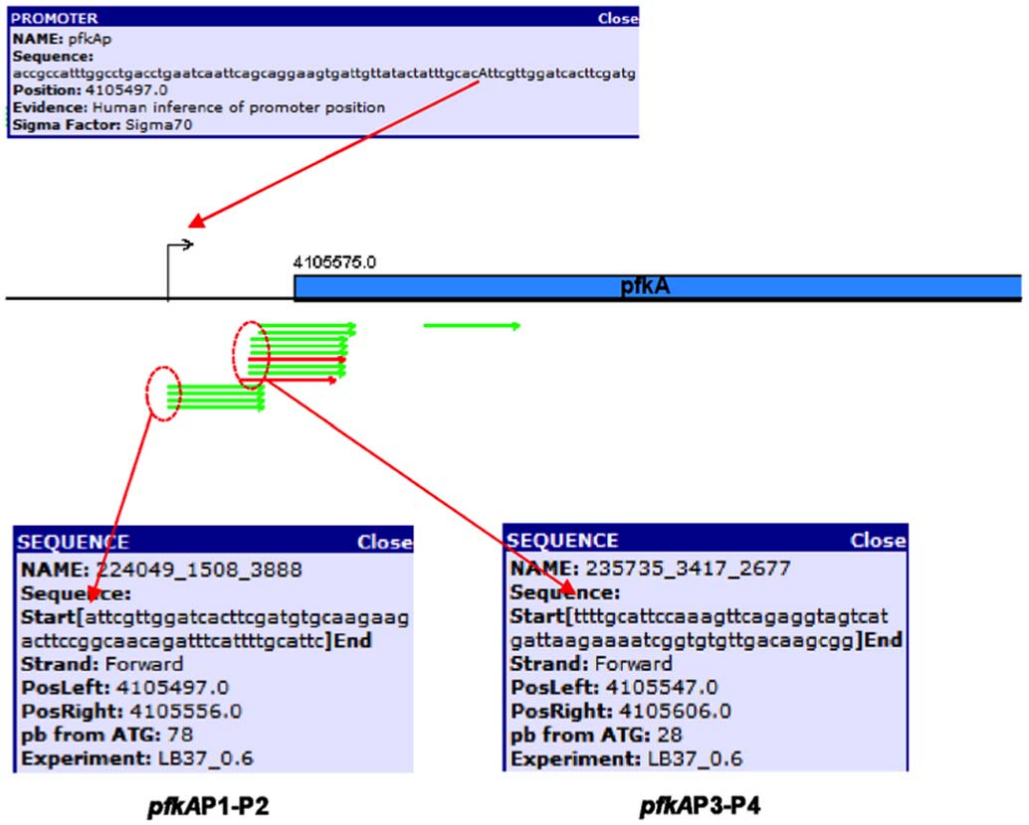
This figure shows the TSSs determined for the *pfkA* gene in an independent global transcription initiation mapping experiment in which random primers were utilized instead of specific oligonucleotides. As can be seen in this experiment in which more than 1500 TSSs were determined (Mendoza *et al*, submitted to PLoS ONE), there are four of these transcripts that initiated at the same nucleotide A (78 nucleotides from the initiation ATG codon). This nucleotide has been reported as the transcription initiation site for the *pfkA* gene [39]. In addition, transcription also occurred in several cases from another site that initiated with a T residue, (29, nucleotides from the ATG start codon). It has been shown that transcription from the *pfkA*P1-P2 promoter is responsible for the previously reported transcription event and, *pfkA*P3-P4 is responsible for a second event (figure 2c).

In a global transcription initiation mapping by pyrosequencing of the wild type *E. coli* strain MG1665 more than 1500 TSSs were determined (Mendoza *et al*, submitted to PLoS ONE). Transcripts from all the glycolytic genes (except *aceEF*, and *lpd*), were detected in this genomic experiment. Twelve TSSs were exactly the same as those reported in table 1, from these, five (*pgi*P1-P2, *pfkA*P1-P2, *gapA*P1P1a, *epd*P1-P2 and *gpmA*P1-P2) had previously been reported or inferred, and seven corresponded to new proposed promoters described in this work (*glk*P3-P4, *pfkA*P3-P4, *tpiA*P1-P2, *pgk*P3, *eno*P4-P5, *pykA*P3-P4 and *pykF*P1-P2). As an example, the TSSs detected for the *pfkA* gene are presented in figure 5.

**Figure 6:**
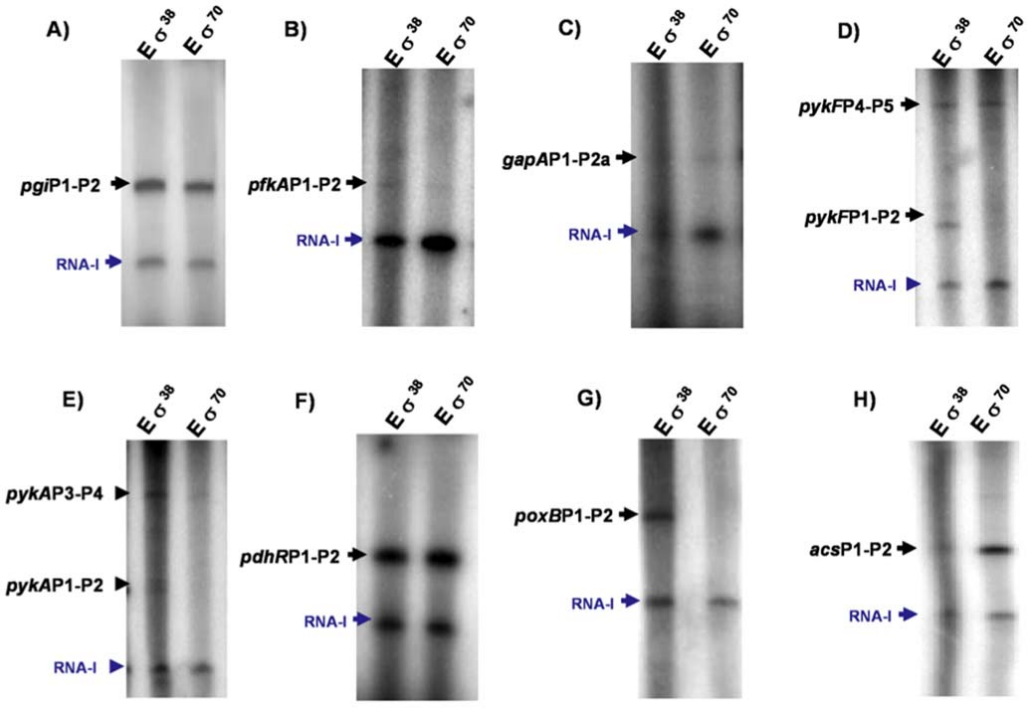
*In vitro* transcription from several promoters by Eσ^70^ and Eσ^38^. The figure shows scans of denaturing 5% polyacrylamide gels of transcriptions generated by RNA polymerase (E) with either Eσ^38^ or Eσ^70^, from supercoiled pSR plasmids carrying different promoters. As can be seen in all the gels, all the promoters were transcribed by both Eσ^70^ and Eσ^38^, with the exception of the *poxB* promoter which is not transcribed *in vitro* by σ^70^. Different intensities indicate varying binding capacities. In all the gels the presence of a plasmid pSR correspondent RNA-I transcript produced with both sigmas, was detected, as previously reported [24]. The expression of this RNA-I was utilized as an internal control, since as expected in all the experiments was present. The sizes of the transcripts and the nucleotide sequences of the DNA fragments carrying these promoters corresponded to the expected for each promoter. Similar results were obtained in duplicate gels for several of the analyzed genes. (For details, see figure S2 and Materials and methods).

*In vitro* transcription experiments demonstrated that several of the proposed putative dual promoters were transcribed by both sigma subunits. For example, in the cases of genes with one detected TSS as *pgi*, *gapA*, *pdhR* and *acs* genes, both σ^38^ and σ^70^ subunits recognized and transcribed *in vitro* the *pgi*P1-P2, *gapA*P1-P1a, *pdhR*P1-P2 and *acs*P2-P3 putative dual promoters. The same was true for genes with more than one TSSs as *pykF* and *pykA* in which *in vitro* transcription from both putative dual promoters *pykF*P1-P2, *pykF*P4-P5, *pykA*P1-P2 and *pykA*P3-P4 was detected. In the case of the *pfkA* gene in which two TSSs were detected, *in vitro* transcription occurred only from the *pykA*P1-P2 putative dual promoter by both sigma factors. Remarkably, the *poxB* promoter was only transcribed *in vitro* by the σ^38^ subunit. Importantly, the same TSSs identified by 5′RACE, were detected in these *in vitro* experiments (figures 6 and S2).

The functionality of some of the proposed putative promoters in *pgi*, *pfkA*, *tpiA*, *pgk*, and *eno*, genes was also demonstrated by fusion of DNA fragments carrying some of these promoters to a reporter gene. DNA fragments carrying the “closest to the ATG initiation codon proposed promoters” (*pgi*P1-P2, *tpiA*P1-P2, *pfkA*P3-P4, *pgk*P2, and *eno*P4-P5) of these genes, were constructed and cloned in front of the *cat* reporter gene which confers chloramphenicol resistance (Cm^r^) present in the plasmid PKK232-8 (see Materials and methods and figure S3). Cells carrying recombinant plasmids were selected for their growth on chloramphenicol. The correct sequence of the cloned DNA segments carrying the different promoters, were verified by gel electrophoresis and nucleotide sequences of the cloned fragments. All these constructions allowed Cm^r^, indicating the functionality of these promoters (figure S3, table 1).

In conclusion, transcription from all the putative proposed promoters reported here by 5′RACE, has been confirmed by at least one other method, except for *gpmA*P3-P4, *eno*P6, *eno*P7-P8, *pykF*P3 and *aceE*P1-P2 (table 1).

### 2. The roles of pyruvate and σ^38^ in carbon flux diversion during carbon limitation in *E. coli*

When *E. coli* is growing slowly on glucose due to low concentrations of this sugar or by PTS inactivation, the genes coding for alternative glucose transport and phosphorylation such as MglB, GalP and Glk are upregulated. In these nutrient scavenging stress conditions, the expression of the σ^38^ modulon that includes the upregulation of several central metabolism genes, such as *poxB* and *acs*, is induced, thus modifying the strategy for carbon utilization [5], [6], [8], [11], [15], [30]. In these conditions PoxB is responsible for pyruvate oxidation, reducing the role of Pdh for pyruvate decarboxylation (see below).

In the wild type strain growing fast on glucose the complete Pdh operon (*pdhR-aceEF-lpd*) is expressed as a polycistronic mRNA, because in these growing conditions the concentration of pyruvate is relatively high, and therefore, there is no repression by PdhR [51]. However, in strains growing slowly on glucose, such as PB11, where the glycolytic flux is reduced, pyruvate concentration is also reduced allowing PdhR to repress transcription of its own promoter, with the subsequent decrease in the transcription of the Pdh operon. This proposal explains higher RTPCR values for the *aceEF* genes in JM101 as compared to PB11, and the result that alanine addition to the growing medium, which can rapidly be converted into pyruvate [62], increased substantially the μ of PB11 and PB11*rpoS* ^−^ strains but not that of PB12 or PB12*rpoS* ^−^, as shown in table 2. These results, as will be discussed in detail, indicate that when *E. coli* cells are growing slowly on glucose or in other non optimal conditions, σ^38^ as the general regulatory stress modulator, performs the regulation of *pdhR*. If low pyruvate concentration is present, this reduces transcription of the Pdh operon. Simultaneously, σ^38^ induces the synthesis of PoxB which is in charge of pyruvate oxidation in stress conditions. Therefore, *E. coli* cells growing slowly on glucose apparently reduce carbon flux through the Pdh system which yields directly AcCoA from pyruvate, and increase the flux through the PoxB enzyme that produces acetate and reducing power at the membrane from pyruvate. Acetate, as part of the gluconeogenic metabolism, is incorporated through Acs as AcCoA into the gluconeogenic “carbon preserving-recycling” metabolism that is present in strain PB11 which grows slowly on glucose [3], [11], [30] (figure 1).

**Table 2:** Specific growth rate determinations (μ = h^−1^) for the PB11 and PB12 strains and their *rpoS*::Tc^r^ derivatives.

Strain	Glucose	Glc + Alanine
PB11	0.13	0.30
PB11*rpoS*::Tc^r^	0.05	0.26
PB12	0.42	0.40
PB12*rpoS*::Tc^r^	0.42	0.44

Alanine was added at a final concentration of 0.5 g/l.

## Discussion

In this work, several TSSs and proposed promoters of all glycolytic genes and operons as well as two other central carbon metabolism genes involved in the transformation of glucose into AcCoA, were identified by a modified 5′RACE strategy and confirmed by pyrosequencing; a total of 17 genes were analyzed. It has been published that in the PTS^−^ strain PB11 which grows slowly on glucose, the transcription level of all glycolytic genes is reduced when *rpoS* was inactivated, indicating that their expression was partially controlled by σ^38^ [11]. The evidence presented in this work strongly supports the proposal that in genes with previously reported σ^70^ promoters (*glk*P1-P2, *pgi*P1-P2, *pfkA*P1-P2, *epd*P1-P2, *gapA*P1-P1a, *pdhR*P1-P2 and *aceE*P1-P2), transcription could occur from two overlapping or putative dual promoters in each gene that included both σ^70^ recognition sites and possible σ^38^ binding sites, in which transcription initiation if directed by both sigma factors, occurred at the same site in all strains. Additionally, new possible σ^70^ and σ^38^ dependent putative dual promoters were described in this report for other glycolytic genes, (*glk*P3-P4, *pfkA*P3-P4, *tpiA*P2-P3, *pgk*P2, *pgk*P3, *gpmA*P1-P2, *gpmA*P3-P4, *eno*P4-P5, *eno*P6, *eno*P7-P8, *pykF*P1-P2, *pykF*P3, *pyk*P4-P5, *pykA*P1-P2, *pykA*P3-P4), in which possible σ^70^ and sequences resembling σ^38^ recognition sites were also overlapped in most of them. No sequences resembling σ^38^ recognition sites were found neither in *pgk* nor in *fbaA*. However, these two genes are part of the *epd-pgk-fbaA* operon and RTPCR data indicate that these three genes could be transcribed by both sigma factors from the *epd*P1-P2 putative dual promoter, in the PTS^−^ strains. PoxB and Acs play an important role in carbon metabolism in the convertion of pyruvate into AcCoA in cells growing slowly on glucose. *pox*B and *acs*, also could have putative dual promoters. We found that *poxB* is mainly transcribed by σ^38^ from the previously reported promoter; however, inactivation of *rpoS* did not abolish its transcription, in agreement with RTPCR results, indicating that this could also be dual promoter. Again, as for many glycolytic genes, a strong putative σ^70^ recognition sequence was detected overlapping this reported σ^38^ promoter. The *acs* gene was mainly transcribed by its originally proposed σ^70^ *acs*P2 promoter. However, in the *rpoS* ^−^ derivative strains its expression, as detected by RTPCR, was reduced, implying that could be also controlled by σ^38^. In agreement with this observation, we found a possible strong σ^38^ recognition element superimposed to the σ^70^ promoter integrating the putative dual *acs*P2-P3 promoter. The experiments presented here, in agreement with RTPCR values indicate that in strain PB11, at least one of the putative dual promoters described for each gene or operon, could be transcribed by σ^38^.

The majority of the TSSs detected in this work lay within the first 100 nucleotides upstream the ATG initiation codons, except for *epd* that initiated transcription 132 nucleotides, *pykA* in which two TSSs were located at 105 and 153 nucleotides, and for one of the three TSSs in *eno* that initiated 212 nucleotides upstream the ATG initiation codon. Remarkably, for those genes with previously reported promoters mapped less than 100 nucleotides from the ATG, we found the same TSSs (except for *tpiA*). On the other hand, for promoters previously described further apart of the ATG start codon (more than 100 nucleotides), as *pykF*, *eno* and *pgk*, we did not find transcription from these sites; instead we detected new promoters for all of these genes located within the first 100 nucleotides upstream the ATG codon. It is important to mention that some of the previously reported promoters (*eno*P1, *eno*P2, *eno*P3 and *pgk*P1) not detected in this report, were described in other metabolic conditions (growing on Luria broth), suggesting differential expressions from these promoters.

The existence and functionality of these new putative dual promoters is supported by the following results: a) most of the previously experimentally determined TSSs and two inferred promoters (*gpmA*P1 and *acs*P2) coincided with the ones obtained here, b) the presence of DNA sequences resembling σ^38^ recognition sites overlapping the σ^70^ binding sites in all these genes, allowed the proposition that these genes could be transcribed from overlapping putative σ^38^ and σ^70^ promoters, defined as putative dual promoters, c) TSSs for most of the genes identified by 5′RACE were mapped in five, and in some cases six different strains. The same results were consistently obtained. In several cases, when the intensities of the bands in the gels were very weak, DNA bands of the same size from different strains were mixed together and sequenced, resulting in the same single TSS. For the majority of the analyzed genes two or more independent experiments were performed, obtaining identical results, d) two different methods for labeling the cDNA ends were employed: polyhomonucleotide tail incorporation and oligonucleotide ligation (developed for cDNA library construction in pyrosequencing, but also used to assess the results of the 5′RACE TSSs mapping), and both methodologies were coincident, e) in most of the genes, the inactivation of σ^38^ reduced the amount of transcript detected in the gel (in agreement with RTPCR values), in at least one of the *rpoS* ^−^ strains as compared to their parental *rpoS* ^+^ strains, f) a global transcription initiation mapping experiment by pryrosequencing of a different *E. coli* K12 strain, in which random primers were utilized instead of gene-specific oligonucleotides (Mendoza *et al*, submitted to PLoS ONE), provided independent evidence for the existence of five previously reported TSSs (*pgi*P1-P2, *pfkA*P1-P2, *gapA*P1-P1a, *epd*P1-P2 and *gmkA*P1-P2) and seven TSSs mapped in this work by 5′RACE (*glk*P3-P4, *pfkA*P3-P4, *tpiA*P1-P2, *pgk*P3, *eno*P4-P5, *pykF*P1-P2, and *pykA*P3-P4), g) promoter fusions of *pgi*P1-P2, *pfkA*P3-P4, *tpiA*P1-P2, *pgk*P2, and *eno*P4-P5, with a reporter *cat* gene, demonstrated the functionality of these DNA sequences, h) *in vitro* transcription experiments for several genes demonstrated that they were transcribed by both σ^38^ and σ^70^, and produced the same TSSs as the ones detected *in vivo*.

σ^70^ and σ^38^ are very similar proteins and in fact genes whose transcription is σ^70^ dependent *in vivo* can often be transcribed *in vitro* by σ^38^ and vice versa However, *in vitro* transcription has been extensively used as evidence to support the expression capability of different sigma subunits [4], [5], [24]. We utilized this technique to generate additional important evidence to support the functionality of the proposed putative promoters. *In vitro* transcription of promoters with one detected TSS as *pgi*, *gapA*, *acs* and *pdhR*, or with more than one as *pykF*P1-P2, *pykF*P3-P4, *pykA*P1-P2 and *pykA*P3-P4 was performed by both sigma subunits (figures 6 and S2), even when DNA sequences resembling very poor σ^70^ and σ^38^ recognition sites were present in the *pdhR*P1-P2, *pykA*P1-P2 and *pykA*P3-P4 putative dual promoters. Transcription of the *poxB* gene clearly occurred *in vitro* when σ^38^ was utilized, in agreement with the fact that a strong σ^38^ recognition sequence is present in this promoter, but not by σ^70^, despite the fact that there is also a σ^70^ recognition element in this dual promoter (figure 4a, table 1). Nevertheless, the results obtained in this report demonstrated that this putative dual promoter was transcribed *in vivo* by σ^70^ in the PTS^−^ *rpoS* ^−^ strains where transcription is clearly reduced as compared to their *rpoS* ^+^ parental strains, but the promoter is still functional (figures 1 and 4a). It is not clear why σ^70^ was unable to transcribe *in vitro* this promoter. Importantly, the same TSSs were detected in the *in vitro* transcription experiments as those reported by 5′RACE, supporting the fact that obtained 5′RACE results are real and not the result of processing. These results also indicate that both sigma subunits are apparently capable of recognizing their binding sites and produce the same transcript as *in vivo*, supporting the proposition that these putative dual promoters could be transcribed *in vivo* by both sigma subunits. It is important to emphasize, as mentioned that since σ^70^ and σ^38^ are very similar proteins this situation could allow the recognition of similar binding sites *in vivo* and *in vitro*, in certain conditions [5].

Remarkably, all glycolytic genes and operons as well as the *poxB* and *acs* genes could have functional putative overlapping dual σ^70^ and σ^38^ promoters, whose positions are at the typical distance from the TSSs. A possible differential utilization of these two sigma factors correlates with carbon availability: σ^38^ plays a major role in the expression of these genes in carbon deprivation stress conditions, while σ^70^ is mainly used when cells are growing under carbon surplus conditions. This interpretation, as it will be explained below in more detail, is supported by two additional facts to those previously described in this discussion: 1) the observation that, as mentioned, inactivation of *rpoS* decreased transcription of all glycolytic genes in strain PB11*rpoS* ^−^ as detected previously by RTPCR, and 2) its inactivation reduced also the growth rate in carbon deprived PTS^−^ strains, particularly in PB11*rpoS* ^−^, but also to some extend in PB12*rpoS* ^−^ which as expected, is less stressed. Wild type JM101 growing exponentially on glucose is less dependent on σ^38^. The functionality of σ^38^ apparently could also hold for several genes previously reported as having σ^70^ promoters, indicating that their expression could be indeed also partially controlled by the former sigma factor. Several of these putative dual promoters have possible strong recognition elements for both σ^38^ and σ^70^, while others have possible weaker elements also for both σ^38^ and σ^70^ factors (figures 2, 3 and 4, table 1). However, it is known that other factors such as DNA structure or interaction with transcriptional regulators, influence the ability of sigma factors to recognize weak binding sites [63]. As it will be discussed below in detail, transcription plasticity, understanded as the capacity of a gene to be transcribed by more than one sigma factor, could have been selected, to allow permanent transcription of the genes involved in the transformation of glucose-6P into pyruvate in different metabolic conditions; remarkably, many of these genes work also as gluconeogenic and their expression is controlled by few general transcriptional regulators. Following, a comparative analysis of the proposed promoters is presented.

### Comparative and detailed analysis of the identified proposed promoters

In the case of *glk* in which two TSSs were identified by 5′RACE: *glk*P1-P2 and *glk*P3-P4; the former, found in PB12, has been previously reported as a σ^70^ dependent promoter [37], while the latter used by strains JM101 and PB11, was also detected by pyrosequencing (table 1). DNA sequences resembling poor σ^38^ and σ^70^ recognition elements were detected in these putative dual promoters of this gene, where inactivation of *rpoS*, apparently increased transcription in the gel from the reported promoters, as detected by PAGE (figures 1 and 2a, table 1). The use of a different promoter in PB12, as compared to JM101 and PB11, could be partially responsible for the higher (2×) level of *glk* transcription detected by RTPCR that correlates with higher (2×) Glk specific activity in PB12 as compared to JM101 [3], [30]. Increased transcription levels from *glk*P1-P2 detected in the gels in PB12*rpoS* ^−^ that do not in agree with RTPCR values; if real, could indicate that *rpoS* inactivation increased the transcription levels from this putative dual promoter, by allowing a more efficient transcription of this promoter by σ^70^. The same type of phenomenon could explain the apparent increase in transcription levels in the gel of the PB11*rpoS* ^−^ strain as compared to PB11 that are also not in agreement with RTPCR values. However, it should be taken into account that 5′RACE is not a quantitative methodology and this could explain these differences. It is important to point out that *glk* is one of the few glycolytic genes whose transcription was not reduced as detected by RTPCR in strain PB11 as compared to JM101. In PTS^−^ strains, and probably in wild type strains growing very slowly on glucose, Glk is the enzyme that phosphorylates transported glucose into glucose-6P. Interestingly, the transcription of this gene was upregulated in a wild type strain grown on glucose as carbon limited nutrient [8], [15]. Nevertheless, it is remarkable that in this gene in which no mutation occurred neither in its regulatory nor in its structural regions in strains PB11 and PB12 [3], apparently two different TSSs are present, indicating that transcription plasticity could allow the utilization of more than one promoter in different metabolic conditions. Apparently, all glycolytic genes could have more than one promoter, supporting this transcription plasticity proposal (see below). The fact that transcription is reduced as detected by RTPCR in both PTS^−^ *rpoS* ^−^ derivatives as compared to their *rpoS* ^+^ parental strains (figure 1), suggests that both *glk*P1-P2 and *glk*P3-P4 could work as dual promoters recognized by σ^38^ and σ^70^.

In the case of genes with only one detected TSS by 5′RACE methodology, such as *pgi*, *tpi*A, *gapA*, *epd* (first promoter of the *epd-pgk-fbaA* operon), *pdhR* (first promoter of the *pdhR-aceEF-lpd* operon), *aceEF* (internal promoter of this operon), *poxB*, and *acs*, *rpoS* inactivation reduced the amount of transcription in these genes as detected by RTPCR and gel electrophoresis (with the exception of *epd* in the gel), suggesting that they are at least partially σ^38^-dependent. As mentioned, possible overlapping strong σ^70^ and strong σ^38^ −10 recognition elements were detected in these genes (with the exception of *epd* and *pdhR*). However, all these promoters (except for *poxB*) seem to be mainly transcribed by σ^70^ (figures 2, 3 and 4, table 1). Transcription from several promoters of this group of genes was also detected by pyrosequencing, and *in vitro* transcription confirmed that *pgi*, *gapA*, *pdhR* and *acs* were transcribed by both sigma subunits, while *poxB* was only transcribed *in vitro* by σ^38^ (figures 6 and 2S, table 1).

In addition to *glk*, there are other genes in which 5′RACE detected two or three TSSs such as *pkf*A, *gmkA*, *eno*, *pyk*F and *pyk*A (figures 2 and 3, table 1). Transcription levels of several of these genes was reduced as detected by RTPCR when *rpoS* was inactivated, suggesting that they could be recognized by σ^38^ [11] (figure 1). This proposition is supported by the presence of putative overlapping dual σ^38^ and σ^70^ recognition elements located upstream of the TSSs of several of these putative dual promoters; in all the cases, possible overlapping σ^70^ and σ^38^-10 recognition elements were present, as it is discussed below:

In the case of *pfkA* a σ^70^dependent promoter part of a *pfk*AP1-P2 putative dual promoter has previously been reported. In this gene a second putative dual promoter *pfk*AP3-P4 utilized in the three strains, was identified (figure 2c). In addition, the same two TSSs were detected for this gene by pyrosequencing supporting their functionality (figure 5, table 1). Possible σ^70^ and σ^38^ overlapping recognition elements were found in both putative dual promoters; in *pfkA*P1-P2 a sequence resembling a strong σ^38^ recognition sequence was detected, whereas a poor one was detected in the *pfkA*P3-P4 putative dual promoter. The proposition that both could be dual promoters is supported by the observation that *rpoS* inactivation in PB11*rpoS* ^−^ decreased transcription as compared to PB11 as detected by gel electrophoresis and by RTPCR (figures 1, 2c, table 1). However, only *pfkA*P1-P2 was transcribed *in vitro* by both sigma subunits (figure 6).

In the *gpmA* gene, two putative dual promoters *gpmA*P1-P2 and *gpmA*P3-P4 were described (figure 3a). Transcription from *gpmA*P1-P2 was also detected by pyrosequencing (table 1). Apparently, in agreement with RTPCR values, inactivation of *rpoS* decreased transcription in strain PB11*rpoS* ^−^ at least from *gpmA*P3-P4. For *gpmA*P1-P2 in this strain transcription seemed unchanged in the gel; however, possible strong superimposed σ^38^ and σ^70^recognition elements were detected in both putative dual promoters *gpmA*P3-P4 and in *gpmA*P1-P2. Interestingly, in this gene and also in *gapA* and *eno*, inactivation of *rpoS* did not have any effect neither in the RTPCR value nor in the transcription level on the gel of strain PB12*rpoS* ^−^ as compared to PB12. These results suggest a more important role of σ^70^ in the transcription of these promoters in this strain with higher glycolytic flux, where RTPCR values were enhanced as compared to the parental strains JM101 and PB11 (figure 1).

As mentioned earlier, transcription of the complete *epd-pgk-fbaA* operon initiated in all strains at the same *epd*P1-P2 putative dual promoter; repeated experiments confirmed these results (figures 2f and S1f, table 1). Inactivation of *rpoS* decreased transcription of all these genes in the *rpoS* ^−^ derivative strains as detected by RTPCR (figure 1); in agreement, a sequence resembling a σ^38^ recognition element was located overlapping the reported σ^70^ recognition site of this promoter. Notice that transcription from *epd*P1-P2 was also detected by pyrosequencing (figure 2f-1, table 1). Two additional TSSs were detected by 5′RACE for the *pgk* gene. Nevertheless, no sequences resembling σ^38^ recognition elements were detected in these *pgk*P2 and *pgk*P3 putative promoters (figure 2f-2). Importantly, transcription from *pgk*P3 was also detected by pyrosequencing, and *pgk*P2 was able to promote transcription of the reporter *cat* gene (figure S3, table 1). In addition, no TSSs were detected in the intergenic region between *pgk* and *fbaA*. These results not only suggest that transcription of this last gene occurred from upstream promoters in this operon, but also that these three genes are apparently transcribed from the putative *epd*P1-P2 dual promoter, as a polycistronic mRNA in the analyzed growing conditions, since inactivation of *rpoS* decreased transcription of all three genes in the PTS^−^ strains (figure 1).

Three different new transcription products for the *eno* gene were detected in several independent 5′RACE experiments. We have labeled the proposed promoters responsible for these expression events as *eno*P4-P5, *eno*P6 and *eno*P7-P8 (figures 3b and S1h, table 1). Importantly, the same TSS for the *eno*P4-P5 was found by pyrosequencing, and this promoter was able to transcribe the reporter *cat* gene (figure S3, table 1). Inactivation of *rpoS* decreased transcription from this gene especially in strain PB11*rpoS* ^−^ as detected by RTPCR. Transcription levels in the gels were slightly reduced from the putative dual *eno*P7-P8 promoter in the PTS*^−^rpoS* ^−^ strains, but not apparently from the putative dual *eno*P4-P5 promoter. Interestingly, these two putative dual promoters carry sequences resembling poor σ^38^ recognition sites (figure 3b, table 1). Again, as in several other cases, it is important to emphasize that 5′RACE is not a quantitative methodology.

In the case of *pykF*, three different putative promoters *pykF*P1-P2, *pykF*P3 and *pykF*P4-P5 were described. Inactivation of *rpoS* apparently decreased transcription levels in the gel of the two putative dual promoters *pykF*P1-P2 and *pykF*P4-P5 that were functional in the PTS^−^ strains (figure 3c-1). Expression from *pykF*P4-P5 was not observed in the wild type strain. This putative dual promoter has strong overlapping possible σ^38^ and σ^70^ −10 recognition elements. In this gene, transcription was performed in the three strains from the three different putative promoters. However, while in all the strains transcription from *pykF*P1-P2 was detected, transcription from *pykF*P3 was only observed in the wild type strain. Metabolic conditions in PTS^−^ strains allowed transcription mainly from the *pykF*P4-P5 apparently σ^38^-dependent promoter, probably inhibiting the utilization of the closely located *pyk*P3 that is functional in the wild type strain. Remarkably, transcription levels determined by RTPCR were slightly diminished in both PTS^−^ strains as compared to JM101 (figure 1). This could be explained by high transcription levels apparently occurring from the *pykF*P4-P5 putative dual promoter in the PTS^−^ strains as shown in the gel. No clear sequences resembling σ^70^ recognition elements were found neither in *pykF*P1-P2 nor in *pykF*P3. However, a sequence resembling a very poor σ^38^ recognition element present in *pykF*P1-P2 could explain lower transcription levels from this promoter in the *rpoS* ^−^ derivative strains including JM101*rpoS^−^* (figure 1).The absence of distinct σ^70^ −10 recognition elements upstream the *pykF*P1-P2 and *pykF*P3 putative promoters allows the speculation that the two transcripts seen in the gel corresponding to these two promoters could be specific processing or degradation products from a larger mRNA produced from the non detected *pykF*P predicted promoter [49]. However, no transcription was detected from this predicted promoter even when using oligonucleotides designed to prime closer to it, ruling out its expression at least in these growing conditions (data not shown). Cra, which recognition site is located 197 nucleotides upstream the ATG initiation codon [49, Regulon DB http://www.ccg.unam.mx] or some other factor, could play a role in the utilization of these putative promoters in which no clear σ^70^ −10 recognition elements were detected. As mentioned, certain factors can facilitate transcription of promoters with poor or no obvious sigma recognition sites [63]. Nevertheless, *pykF*P4-P5 and *pykF*P1-P2 could be transcribed by σ^38^ since inactivation of this sigma factor reduced transcription in the gel of these two putative dual promoters in the *rpoS* ^−^ derivative strains. These experiments suggest that they could be real promoters and, as mentioned, the wild type strain transcribed this gene from both *pykF*P1-P2 and *pykF*P3 promoters in the absence of σ^38^, such that σ^70^ is apparently controlling their expression in this strain. In fact, *in vitro* transcription indicates that the two putative dual promoters *pykF*P1-P2 and *pykF*P4-P5 are recognized by both sigma factors and transcription initiation from *pykF*P1-P2 has been detected by pyrosequencing (figures 6 and S2, table 1). Therefore, these two putative dual promoters seem to be functional in these strains. It is important to emphasize that in *E. coli* strains lacking PTS, the absence of this general regulator increases the carbon flux through the PykA and PykF enzymes, and apparently in these PTS^−^ strains the *pykF* gene seems to be preferentially transcribed from the *pykF*4-P5 putative dual promoter, which is not functional in the wild type strain when growing exponentially on glucose.

In *pyk*A, two putative dual promoters were detected in the three strains by 5′RACE. Clearly, these two promoters were transcribed in JM101 indicating that σ^70^ is performing this expression. While inactivation of *rpoS* apparently decreased transcription in the gel from *pykA*P3-P4 putative dual promoter in PB12*rpo*S^−^ and possibly in PB11*rpo*S^−^, it is interesting that this inactivation apparently increased transcription in the gel from *pykA*P1-P2 in both PTS^−^ strains (figure 3c-2). The same type of phenomenon of increased transcription detected in the gel for two promoters occurred in *glk*. Importantly, both *pykA* promoters have DNA sequences resembling poor σ^70^ −10 recognition elements and also DNA sequences resembling very poor σ^38^ recognition sequences. However, inactivation of *rpoS* markedly decreased transcription levels (more than 50%) of *pykA* in both PTS^−^ *rpoS* ^−^ strains as compared to their respective parental *rpoS* ^+^ derivatives, as detected by RTPCR (figure 1), suggesting an important role of this sigma factor in the expression of *pykA*. In fact, *in vitro* transcription demonstrated that both putative dual promoters are transcribed by σ^38^ and σ^70^ (figures 6 and S2). Remarkably, *in vitro* transcription of these two putative dual *pykA* promoters by both sigma fagctors, rendered weaker bands than those corresponding to other promoters, including the one in the plasmid responsible for RNA-I. These results suggest, as has been proposed for the *pykF*P1-P2 promoter in which a DNA sequence resembling a poor σ^70^ recognition site is present that *in vivo* some other factor could play a role in the utilization of these *pykA* putative dual promoters where DNA sequences resembling weak σ^70^ and σ^38^ recognition sites are present [63]. Transcription from the putative *pykA*P3-P4 dual promoter has also been detected by pyrosequencing. Interestingly, higher RTPCR values were present in the transcription of this gene in PB12 as compared to PB11, while the same transcription level was exhibited in both strains in the *pykF* gene as detected by RTPCR. Therefore, it is possible that during the selection of PB12 for growth recovery on glucose the transcription of several genes, including *pkyA* was upregulated as compared to PB11, increasing in turn the glycolytic carbon flux. Clearly, there is a differential utilization of the *pykA* promoters in strain PB12 as compared to PB11. Apparently, this latter strain utilizes preferentially the putative dual promoter *pykA*P1-P2 whereas PB12 transcription of *pykA*P3-P4 is highly increased in comparison to the parental PB11 strain. Differential utilization of promoters is also apparently present in the *glk* gene and clearly upregulation of the expression of most glycolytic genes in PB12, as compared to PB11, as revealed by RTPCR (figure 1) is also detected in several other genes (*pgi*, *gapA*, *pfkA*, *tpiA*, *pdhR*, *aceE*) in the gels. These results could explain in part, faster growth of PB12 over PB11.

In all strains, transcription of the *pdhR-aceEF-lpd* operon initiated at the same *pdhR*P1-P2 putative dual promoter, upstream of *pdhR*. In derivative strains lacking σ^38^, not only transcription levels of *pdhR* were diminished, as seen from the RTPCR values (figure 1), but also the intensity of the bands in the gels (figure 3d-1). These results suggest that this promoter region may include functional overlapping σ^70^ and σ^38^ promoters and are in agreement with the report that σ^38^ modulates the expression of *pdhR* [5], [51]. As mentioned, *in vitro* transcription provided additional evidence to support the fact that both sigma subunits recognize and transcribe this putative dual promoter (figures 6 and 2S, table 1). Interestingly, possible poor σ^70^ and possible poor σ^38^ overlapping recognition sites are present in this putative dual promoter. Independently from the results concerning the TSS in the *pdhR* gene, the data presented here also indicate that an *aceEF* internal putative dual promoter is apparently functional in all strains, and may work as a constitutive promoter because PdhR does not repress it [51]. This is in agreement with the result that in all strains the TSS at the *aceE* gene coincided with the reported one [50]. Again, transcription from this DNA region carrying both possible σ^70^ and σ^38^ superimposed boxes apparently depended on both σ^70^ and σ^38^ and transcription was decreased in PB11*rpoS* ^−^ and more evidently in PB12*rpoS* ^−^ strains, as compared to their parental strains. Finally, upregulation of *pdhR* was detected in PB12 (figure 1) and in another wild type strain grown on Luria broth in different stress conditions [5]. However, less upregulation was detected in *aceEF* (1.4×) in PB12 whereas no upregulation of these two genes was reported in a wild type strain grown on Luria broth in different stress conditions [5]. These results indicate that a transcription termination mechanism could be present at the end of the *pdhR* structural gene allowing lower upregulation values of *aceEF* in PB12 and in other *E. coli* strains [5] as compared to *pdhR*, since these genes are mainly transcribed as part of a polycistronic mRNA. In this regard, a possible terminator sequence has been described at the end of *pdhR* structural gene that could explain these differences [50], [51].

Our results show that *rpoS* inactivation decreased transcription in several previously reported σ^70^-dependent promoters in glycolytic genes and operons: *pgi*P1-P2, *pfkA*P1-P2, *gap*AP1-1a, *epd*P1-P2, *pdhR*P1-P2, *aceE*P1-P2. In fact, in these six genes which have the same reported transcription initiation sites and are mainly transcribed by σ^70^, six new proposed putative σ^38^ promoters superimposed to σ^70^ recognition sequences, were described in these putative dual promoters. Several of these new possible σ^38^ sites are strong recognition sequences. In addition, new dual putative promoters were described: *tpiA*P2-P3, *pykF*P1-P2, *pykF*P4-P5, *pykA*P1-P2, *pykA*P3-P4 and possibly *glk*P1-P2, *glk*P3-P4, *pfkA*P3-P4, *gpm*AP1-P2, *gpmA*P3-P4, *eno*P4-P5, and *eno*P7-P8 that all seem to include both σ^70^ and σ^38^ superimposed possible recognition elements. Importantly, some of these putative dual promoters in this last group carry DNA sequences resembling poor σ^38^ recognition sequences especially *pykF*P1-P2, *pykA*P1-P2, *pykA*P3-P4, *pfkA*P3-P4, and *eno*P7-P8 (figures 2 and 3, table 1). However, inactivation of *rpoS* in strain PB11*rpoS* ^−^ reduced the transcription levels of all these genes detected by RTPCR, suggesting, as previously mentioned that at least one of the putative dual promoters in each gene or operon could be transcribed by σ^38^ (figure 1). In addition, the independent global transcription mapping experiment allowed the identification by pyrosequencing of the same TSSs detected for twelve of these proposed dual promoters, and fusion of chromosomal DNA fragments carrying one or more of these promoters to a reporter gene (figure S3, table 1), corroborated the functionality of some of these promoters. Finally, *in vitro* experiments confirmed that several proposed dual promoters are transcribed in these experiments by both sigma subunits (figure 6). These results indicate that σ^38^ plays a central role in the regulation of all glycolytic genes and operons and at least in two other important central metabolism genes -*poxB* and *acs*- that could be at the transcription level since possible superimposed σ^70^ and σ^38^ putative dual promoters were detected in all these genes when *E. coli* cells were grown slowly on glucose, as occurred in the PTS^−^ strains, specially in PB11. The presented results allowed the identification of fourteen new TSSs by 5′RACE and the proposal of more than 30 putative promoter sequences in 17 central metabolism genes and operons not previously described. The majority of these putative promoter sequences could be recognized by both σ^38^ and σ^70^ sigma factors (table 1). Certainly as mentioned, some of these putative promoters carry poor σ^70^ and σ^38^ binding sites. If these promoters are real and they are transcribed by both sigma subunits, other factors such as DksA, Crl, Cra, Fis, H-NS and IHF, could modulate the binding capacities and selectivities of these sigma subunits in these promoters [5], [6], [16], [24], [63].

### Alternative scenarios that could also explain the low transcription levels in the PTS^−^ *rpoS* ^−^

It is important to emphasize that some of the proposed dual promoters have possible poor −10 σ^38^ recognition sequences especially those located in *pfkA*P3-P4, *eno*P7-P8, *pykF*P1-P2, *pykA*P1-P2 and *pykA*P2-P3 (table 1). Therefore, if any of the putative σ^38^ recognition elements that have been described in this report are really not functional *in vivo*, then an alternative explanation for the decreased RTPCR values in *rpoS* ^−^ derivatives as compared to their *rpoS* ^+^ parental strains, could be that the transcription of these genes in these PTS^−^ strains must depend indirectly on σ^38^. This hypothesis entails that some other factor(s) of the σ^38^ regulon could modulate transcription by directly interacting with the transcription machinery or indirectly, allowing differential exposure of certain DNA regions required for transcription. Weber *et al* [5], have proposed that σ^38^ containing RNAp holoenzyme has the ability to cooperate with additional regulatory factors, similar as σ^70^ RNAp does. Therefore, some of these regulatory factors that interact with the vegetative σ^70^ RNA polymerase could be part of the sigma σ^38^ modulon. Hence the absence of one or some of these factors could have a negative impact on the transcription of certain σ^70^ promoters. An additional alternative possibility that could also explain, at least partially, lower transcription levels in glycolytic genes in strain PB11*rpoS* ^−^ as compared to PB11 is the fact that the lack of σ^38^ may decrease the levels of acetic acid produced by PoxB in these strains, which is used as carbon source through the activity of Acs; this could reduce gluconeogenic and glycolytic fluxes that in turn may decrease both growth rate and the transcription of glycolytic genes in the PB11*rpoS* ^−^ strain. We have published that inactivation of *poxB* reduced 50% the specific growth rate of PB11*poxB* ^−^ as compared to PB11. We have proposed that less acetate should be produced in the PB11*poxB* ^−^ strain, as well as in the PB11*rpoS* ^−^ strain, since *poxB* is transcribed by σ^38^, to be recycled as a carbon source and therefore this situation could be as mentioned, at least partially, responsible of a decrease in the transcription of glycolytic genes. In fact, transcription of some glycolytic genes is reduced in PB11*poxB* ^−^ when compared to PB11 [30]. It is known that acetate could have a role, directly or indirectly, in the expression of several genes among those coding for regulatory proteins, including those which regulate or belong to the transcription machinery like *rpoS* and RpoS [3], [11], [64]. Therefore, we compared the expression levels of glycolytic genes in the *poxB* ^−^ and *rpoS* ^−^ derivative strains with the goal of determining the possible roles of σ^38^ and acetate in their expression. As mentioned, the transcription values of two of the analyzed glycolytic genes *glk* and *pgi*, are reduced roughly at the same levels in the PB11*poxB* ^−^ and PB11*rpoS* ^−^ strains as compared to PB11 [11], [30], allowing the possibility that, low acetate levels could play a role in the expression of at least these two genes. However, since the reported transcription values of other glycolytic genes (*aceEF*, *lpd*, *pykA*, *pykF*), in the PB11*poxB* ^−^ strain are not modified in comparison with PB11, this result indicates that at least in these five genes [30], lower acetate levels are apparently not responsible of reducing their expression in the PB11*rpoS* ^−^ strain. In addition, the expression of the seven analyzed glycolytic genes in the PB12*poxB* ^−^ strain is not modified in comparison to the PB12 strain [30]. These results support the proposition that σ^38^ could play a role in the expression of these genes. Therefore, if these putative dual promoters really function *in vivo* and if several of them are transcribed by σ^38^, this may indicate that transcription initiation by σ^38^ is in fact less affected by different deviations from the proposed promoter consensus sequence [5]. Degeneration of the −35 recognition sequences has been associated with the capacity of σ^38^ to transcribe “non optimal promoters” [5], [6], [21]. It appears that this could also occur in the −10 region of the promoter because some of the proposed −10 regions in some of the analyzed genes have relatively poor σ^38^ recognition sequences.

### Transcription plasticity in central carbon metabolic genes and operons could allow differential expression of these genes by σ^38^ and σ^70^ subunits

Transcription plasticity, defined here as the capacity of a gene or an operon to be transcribed by more than one sigma subunit, could be present in all the analyzed genes and operons. This capability could allow differential expression of those genes by the two sigma subunits. Accordingly, in several cases the strength of a specific promoter in one gene was diverse in the various strains with different genetic backgrounds and in different metabolic conditions. Importantly, most of these different sigma possible recognition sites described in this report that could allow transcription plasticity, are superimposed as putative dual promoters. Therefore, transcription plasticity could be present not only at the gene level, but also at the promoter level. Thus, RNAp with different σ factors holoenzymes could perform transcription, depending on the metabolic conditions and as suggested in this report, as an adaptation response to carbon limitation in cells lacking PTS^−^ growing slowly on glucose in which as mentioned, due to the absence of PTS, higher levels of σ^38^ are present [3], [4]. Alarmones, as ppGpp, are involved in modulating sigma subunits affinities for the RNA polymerase core. Therefore, depending on carbon availability that is translated into different ppGpp concentrations, *E. coli* cells could differentially utilize certain possible dual promoters for the expression of glycolytic genes: less carbon, more sigma σ^38^ RNAp holoenzyme. Certainly, other factors such as DksA and Crl, modulate the binding capacities of the different sigma subunits to the RNA polymerase core and also transacting transcriptional regulators such as Fis, IHF and H-NS contribute to promoter plasticity and selectivity [6], [16], [24], [27]. Recently, it has been reported that Fis and H-NS modulate the expression of the *dps* gene by allowing preferential utilization of the *dps* promoter a putative dual promoter, by σ^38^ over σ^70^ when cells reach the stationary phase. These results suggest that the cells can modulate the expression of different promoters in the same gene by allowing the preferential utilization of σ^38^ over σ^70^ for transcription in cells growing in non optimal conditions [24]. In agreement, the presence of both σ^38^ and σ^70^ putative dependent promoters in all genes and operons of the glycolytic pathway studied and in other important central metabolism genes such as *poxB* and *acs*, could allow the transcription of these central metabolic genes by the cells in both high (optimal) and low (starvation) glucose growing conditions. As mentioned earlier in most cases promoter plasticity could be achieved by selecting superimposed dual promoter sequences for both σ^70^ and σ^38^ and in other cases, not superimposed promoters could be selected. In the first instance, the cell could have selected this type of superimposed promoters to ensure that transcription initiation is the same in different metabolic conditions; whereas transcription initiation from different promoters could allow a differential postranscriptional regulation. As an example of this case, a new putative dual promoter apparently appeared in the *pykF* gene in cells lacking PTS. This promoter is preferentially used in these strains in this metabolic node where carbon flux is increased due to the absence of PTS. Importantly, most glycolytic genes are upregulated in PB12 as compared to PB11 as the result of the selection process. As proposed, at least in some of these upregulated genes (*glk*, *gapA*, *tpiA*, *pykA*) in PB12, higher transcription could be the result of the differential utilization of σ^38^ and σ^70^. Finally, promoter plasticity could be the result of promoter degeneracy from the vegetative σ^70^ sequence. [6], [27]. As previously mentioned, the analysis of the σ^32^ regulon revealed an extensive overlap between its targets and those of σ^70^, as in the case of the σ^38^ regulon. Some degree of promoter degeneration that could be translated into promoter plasticity, probably could allow different RNAp holoenzymes to share transcriptional start sites in many genes [25]–[27].

Additionally, there are metabolic steps in which both glycolytic and gluconeogenic enzymes coded by different genes are present. In these cases, the expression of the genes coding for gluconeogenic enzymes, such as *fpb*, *fbaB* and *ppsA* [38], was clearly upregulated in strains growing slowly on glucose as PB11 (figure 1) [3], [11]. Also in this strain other genes as *gapC*1, *gapC*2 and *gmpB* were upregulated. As expected, in some of these genes, sequences resembling σ^38^ recognition elements have been identified and, accordingly, σ^38^ inactivation downregulated their expression [5], [11], [65, Regulon DB http://www.ccg.unam.mx 2008]. Therefore, these genes, together with *fpb*, *fbaB* and *ppsA* that are also upregulated in PB11, could also be involved in the gluconeogenic pathway. Nevertheless, it is important to emphasize that the identification of new possible σ^38^ recognition elements reported in this work has been detected in glycolytic genes whose products work only as glycolytic enzymes such as *glk*, *pykF*, *pykA* and *aceEF* and in genes whose products work in both glycolytic and gluconeogenic reactions such as *pgi*, *tpiA*, *eno*, *pgk*. Sequences resembling σ^38^ recognition sites have been also identified in the *pfkA*, *gapA* and *gpmA* genes whose coded proteins seem to work as well as glycolytic and gluconeogenic enzymes [38].

It is possible that most of the described putative dual promoters could also work with gluconeogenic substrates such as acetate. When this compound is used as carbon source simultaneously with glucose in minimal medium, these PTS^−^ strains coutilize both carbon sources due to the lack of catabolite repression with a subsequent increase in their μ [3]. In fact, it has been demonstrated that in addition to those genes mentioned above, which work both as gluconeogenic and glycolytic, the inactivation of *rpoS* decreases transcription levels of most gluconeogenic and putative gluconeogenic genes as: *aceA*, *aceB*, *acnB*, *fbaB*, *fbp*, *glcB*, *gapC*-1, *gapC*-2, *ppsA*, *sfcA* in the PTS^−^ strains, indicating a role for this sigma factor in their expression when growing on glucose as the only carbon source [11]. At least in some of these genes, putative overlapping σ^70^ and σ^38^ possible recognition elements have been detected (unpublished observations).

Therefore, the presence of both types of putative promoters usually superimposed, could allow the cells the possibility of using different sigma subunits for transcribing a gene in different metabolic conditions. Interestingly, this capacity could be very important, especially in the upper part of the glycolytic pathway, responsible of the transformation of glucose-6P into pyruvate, in which most of the reactions, except for the *pyk* isoenzymes, are reversible as part of the gluconeogenic pathway. Remarkably, very few transcriptional regulators modulate the expression of this group of glycolytic genes, as discussed in the next section.

### Expression of the genes involved in the transformation of glucose-6P into pyruvate seems not to be substantially regulated by general transcription regulatory elements. The presence of different promoters in these genes could allow a permanent transcription response in different metabolic conditions

Remarkably, few general transcription regulators have been reported to be involved in modulating the expression of most glycolytic genes -Cra and Crp, among others- especially at the upper part of the glycolytic pathway involved in the transformation of glucose-6P into pyruvate. Cra works as a repressor of certain glycolytic genes such as *ptsHI*, *pfkA*, *pykF*, and the *epd-pgk-fbaA* operon and as an activator of some gluconeogenic genes. When wild type *E. coli* grows fast aerobically on glucose, such as strain JM101, Cra whose DNA binding activity is modulated by sugar catabolites (fructose-1-P and fructose-1-6P), does not significantly repress glycolytic genes. This explains why Cra inactivation exerts no effect on the growth rate of the wild type *E. coli* [66], [67]. In gluconeogenic conditions, where *E. coli* grows relatively slow, transcription of glycolytic Cra regulated genes should be decreased, while transcription of Cra regulated gluconeogenic genes should be increased; in agreement, *cra* inactivation inhibits growth on gluconeogenic substrates. These results can be explained by the fact that sugar catabolites present at higher concentrations when growing rapidly on glucose, bind to Cra and displace it from the operator sites in target genes. However, in gluconeogenic conditions these sugar catabolites are present in relatively lower concentrations thus allowing Cra to repress glycolytic and activate gluconeogenic genes [49], [66], [67]. In strain PB11 that grows slowly on glucose sharing some responses of the gluconeogenic metabolism, particularly the fact that sugar catabolites are probably in lower concentrations as compared to the wild type, the transcription of glycolytic genes was downregulated (with the exception of *glk* and *pgi*), as compared to JM101. The possible role of Cra in modulating the expression of *pfkA* and *pykF* genes and the *epd-pgk-fbaA* operon downregulated in PB11, could be part of a more general mechanism, in which due to the absence of PTS, not only the Cra regulated genes but most glycolytic genes were downregulated in PB11. Nevertheless, downregulation of these genes may be due in part to Cra repression; however, *pykF* and *epd-pgk-fbaA* transcription in both PTS^−^ strains was only slightly downregulated as compared to JM101, suggesting, a minor role of Cra in the expression of these genes [66], [67] in the tested conditions. Crp regulates the expression of the *gapA* gene and also the *epd-gmk-fbaA* operon. However, the expression of *gapA* in these strains in these growing conditions with glucose as the only carbon source, occurred only from the *gapA*P1 reported promoter which is not regulated by Crp [41], [42]. Therefore, neither Crp in the case of *gapA* nor Cra for *pykF* and the *epd-pgk-fbaA* operon were apparently significantly involved in modulating the expression of these genes in the tested conditions. *E coli* in its natural environment must rapidly adjust its metabolism from conditions with high to low glucose concentrations [7], [8], [12], [15]. The glycolytic pathway, from glucose-6P to pyruvate, is a flexible pathway in which, as mentioned, most of the steps work in both directions, allowing the utilization of gluconeogenic substrates to produce glucose-6P. In fact, the glycolytic and gluconeogenic pathways work simultaneously in PTS^−^ strains that grow slowly on glucose. The apparent absence of regulation at the level of transcription of most glycolytic genes involved in the transformation of glucose-6P into pyruvate, except the ones mentioned above, may indicate that the modulation of the expression of the genes coding for this critical reversible pathway is being performed by a different strategy. Apparently, *E. coli* could have selected a less transcriptional regulated glycolytic pathway at least in the initial part of the pathway, in which typical repressors or activators are less involved, in order to allow permanent transcription of the genes involved in these reactions in different metabolic conditions. Transcription plasticity and promoter redundancy, utilizing several sigma subunits, could allow a more flexible transcriptional regulatory strategy for this section of the glycolytic pathway, in which several reactions may also be gluconeogenic.

### The role of σ^38^ in the transformation of pyruvate into AcCoA in different growing conditions: carbon flux divertion during carbon limitation

Nevertheless, there are some critical metabolic steps of the central carbon metabolic pathway that are clearly transcriptionally regulated, such as the transformation of pyruvate into AcCoA performed by both Pdh and PoxB/Acs in different growing conditions. As mentioned, in the transformation of pyruvate into AcCoA, PdhR works as a specific transcriptional repressor of the Pdh operon when pyruvate is present at low concentrations; its expression is controlled by Crp and regulated by σ^38^ [5], [11], [51]. In agreement, σ^70^ and σ^38^ superimposed overlapping putative promoters were reported in this work in the *pdhR* gene. In conditions where bacterial cells are growing slowly on glucose, *E. coli* apparently utilizes PoxB for pyruvate oxidation into acetate, reducing the role of Pdh for pyruvate decarboxylation (figure 1). These propositions are supported by the facts that PoxB inactivation in strain PB11*poxB* ^−^ reduced μ by 50% whereas in JM101 the effect was minor (5% reduction in μ). In PB11, *aceEF* transcription values were reduced, while *poxB* and *acs* were upregulated as compared to JM101. Additionally, the carbon flux between pyruvate and AcCoA is doubled in the PTS^−^ strains [3], [11], [29], [30], [55]. Importantly, transcription of *pdhR* is not upregulated in PB11 (figure 1), but is upregulated in other stress conditions in a wild type strain as previously mentioned [5]. Furthermore, it has been demonstrated that in cultures with acetate as the only carbon source, the addition of alanine which can rapidly be converted into pyruvate [62], increases the μ of strains PB11 and PB12, indicating intrinsic pyruvate deficiencies in these strains [64]. In fact, a similar effect was observed in strains PB11 and PB11*rpoS* ^−^ growing on glucose. As mentioned, the addition of alanine increased their μ substantially (table 2). These results are in agreement with the proposition that in strain PB11, in which low pyruvate concentrations are apparently present, the addition of alanine, increased its μ 100%, because in these conditions more pyruvate is probably utilized by Pdh instead of PoxB due to the lack of repression of the transcription of *pdhR-aceEF-lpd* operon by PdhR in the presence of pyruvate [51]. This effect is more dramatic in strain PB11*rpoS* ^−^ where the absence of σ^38^, and consequently PoxB, has an important detrimental effect on the μ, since no carbon can be recycled through PoxB/Acs. In this strain, the addition of alanine increased μ in 250%, reaching a specific growth rate, only 25% lower than PB11, indicating again that in these conditions with high pyruvate concentrations due to the addition of alanine, PB11 utilizes preferentially the Pdh complex, reducing the role of PoxB, to convert pyruvate into AcCoA. Interestingly, no effect was detected when alanine was added to cultures of strain PB12 and PB12*rpoS* ^−^, in agreement with the higher glycolytic flux in PB12 where pyruvate should be present in higher concentrations. These results support the metabolic carbon flux diversion proposed at the pyruvate node. In conclusion, *E. coli* has two alternative routes to produce AcCoA from pyruvate that are differentially utilized in distinct growing conditions.

Diversion of metabolic flux from Pdh to PoxB is also apparently involved in decreasing oxidative stress during glucose metabolism in non growing *E. coli* cells under aerobic phosphate starvation conditions [68]. In fact in PB11, *soxS* is upregulated as compared to JM101 (unpublished results). Therefore, it appears that this carbon flux diversion is a more general strategy to deal with different stress conditions including slow growth on glucose. We emphasize that σ^38^ is directly involved during growth on non optimal conditions, in two aspects: 1) in the regulation, possibly by transcription of the *pdhR* gene whose product decrease the expression of the Pdh operon in the absence of pyruvate, and, 2) in the transcription that allows upregulation of *poxB*, which allowed carbon flux diversion, during carbon limitation, from Pdh to PoxB. This metabolic adaptation may allow cells growing slowly on glucose the possibility of preserving carbon atoms by reducing CO_2_ production at the TCA, probably by recycling higher amounts of AcCoA into the glyoxalate shunt and the gluconeogenic pathways for carbon preservation purposes. Remarkably, strong superimposed σ^70^ and σ^38^ putative dual promoters were also located in the *poxB* and *acs* genes that code for the enzymes that perform this important “Pox-Acs-shunt” involved in the transformation of pyruvate into acetate, with concomitant generation of reducing power at the membrane and the recycling and incorporation of acetate into AcCoA (figure 1) [3], [7], [8], [11], [28]–[30], [57], [64], [68], [69].

### Comments on the differential expression of some of these genes in the evolved strain PB12 as compared to PB11

During the selection process performed to isolate PB12, several mutations occurred and one (or more) of them allowed the upregulation of glycolytic genes that permitted faster growth on glucose (3, 11, unpublished results). However, the expression of these genes remained upregulated in a gluconeogenic substrate as acetate, suggesting that this general mechanism which has been modified is responsible for the upregulation of the glycolytic genes in both glycolytic and gluconeogenic growth conditions [64]. In addition, as mentioned, a possible differential utilization by σ^38^ and σ^70^ of the proposed putative promoters in these genes, could also play a relevant role in their differential expression in these genes in these strains. Interestingly, as mentioned, inactivation of *rpoS* clearly has not an important effect on the growth rate of PB12, as compared to PB11. In agreement, RTPCR values of many glycolytic genes in PB12 are not substantially reduced in the PB12*rpoS* ^−^ derivative as compared to PB12, indicating a minor role of σ^38^ in the regulation of many of the glycolytic genes (figure 1). Remarkably, in this strain there is upregulation (3.5×) of the genes (*gpp*, *spoT*, *ndk*, *ppa*) involved in ppGpp metabolism. This situation could modify, probably reducing, the concentrations of this alarmone in PB12 as compared to PB11, and in turn could allow a differential utilization of these sigma subunits by the RNAp [11].

The analysis of the mutations that occurred in PB12 will allow a better insight into the physiology of *E. coli* strains that grows permanently stressed on glucose such as PTS^−^ strains and as well as a deeper understanding on the metabolic plasticity of this bacteria that has the capability of generating new metabolic adaptive capabilities in the absence of the general regulatory PTS.

## Materials and Methods

### 1) Bacterial strains, media and growth conditions

*E. coli* strain PB12 (JM101 Δ(*ptsH*, *ptsI*, *crr*)::*kan*, Glc^+^) was obtained from PB11 Δ(*ptsH*, *ptsI*, *crr*::*kan*), a PTS^−^ mutant derivative of strain JM101 (F′ *tra*D36 *proA^+^ proB^+^ lac*I^q^ Δ(*lac*Z)Z15/*supE thi* Δ(*lac*-*proAB*)) [28], [70]). Derivatives of these strains lacking *rpoS* were previously obtained by P1 transduction using strain RH90 *rpoS*::TC^r^ as donor of *rpoS*::Tc^r^ [11]. These strains were utilized for isolating RNA that was previously used for RTPCR transcription experiments; the same RNA was used for the modified 5′RACE methodology. Wild type *E. coli* strain MG1665, was used for the extraction of RNA that was utilized for the global transcription experiment (Mendoza *et al*, submitted to PLoS ONE). Duplicate cultures for RNA isolation, were grown aerobically in 1 l fermentors with 700 ml of M9 minimal medium with 2g/l of glucose as the only carbon source, at 37°C, 600 rpm controlling pH at 7 with NaOH and air flow rate of 1 vvm. Bacterial growth was monitored and cells were collected at 1.0 (OD 600 nm) when growing in the log phase [3]. Strain Top 10 (Invitrogen) was used as the recipient of DNA fusions carrying specific promoters with a reporter gene.

### 2) Construction of DNA fusions carrying promoters of several glycolytic genes with a reporter cat gene that confers chloramphenicol resistance (Cm^r^)

DNA fusions carrying different chromosomal DNA regions with the “closest to the ATG promoters” of various glycolytic genes (*pgi*, *pfkA*, *tpiA*, *pgk*, and *eno*), were constructed using a *cat* reporter gene, as previously described [71]. Briefly, chromosomal DNA regions were constructed carrying the promoters of the selected genes and the first 20 bp of the specific structural gene. The nucleotide sequences of the oligonucleotides that were utilized for amplifying the selected specific DNA chromosomal fragments are presented in table S1. In all cases two oligos were used for isolating the closest to the ATG promoter present in these genes: one forward (Fw) and one reverse (Rv). Each different fragment was amplified, and the product ligated into *SmaI* digested pKK232-8; this site is located in front of the *cat* reporter gene in this plasmid (figure S3) [71]. Recombinant molecules were used to transform *E. coli* strain Top 10. Transformed cells were plated on minimal medium with glucose and chloramphenicol (10 µg/ml). Alkaline minipreps of different construction were performed and plasmids were digested with *PstI* to verify the size of the ligated chromosomal DNA. The nucleotide sequence of the cloned inserts, was also determined (figure S3).

### 3) RNA Extraction

Total RNA extraction was performed using a hot phenol method. After extractions, RNA was precipitated with 3 M sodium acetate/ethanol and centrifuged at 20,000*g* at 4°C. Supernatant was discarded and the RNA resuspended in water and treated with Dnase kit (DNA-free™, Ambion, USA). RNA concentrations were determined by absorbance at 260/280 nm ratio. RNA integrity was verified by electrophoresis in agarose gels [3], [11].

### 4) Construction of cDNA libraries using 5′RACE for TSSs mapping

For determining the TSSs of the glycolytic, *poxB* and *acs* genes we used the 5′RACE methodology [72], [73] with some important modifications. A more detailed protocol is described in a separate report (Mendoza *et al*, submitted to PLoS ONE). Briefly, we generated cDNA libraries for each strain (*E. coli* JM101, PB11, PB12 and their *rpoS* ^−^ derivatives) by reverse transcription using SuperScript III reverse transcriptase (Invitrogen, Carlsbad, USA) together with an hexamer random primer-adaptor B (5′GCCTTGCCAGCCCGCTCANNNNNN3′). The cDNA synthesis reactions were incubated in a RoboCycler equipment (Stratagene, Amsterdan, The Netherlands) under the following program (28°C for 20 min, 45°C for 40 min, 70°C for 10 min). The cDNA final products were purified using the High Pure PCR product purification kit (Roche Indianapolis, USA), according to the manufacture's instructions. Purified cDNA libraries were labeled (tagged) at the 3′ terminal end using two approaches. In the first one, the 3′ terminal end was labeled with a tail of adenine polynucleotide using a terminal transferase reaction. In this case, 20 µl of purified cDNA sample was mixed with terminal deoxynucleotidyl transferase (20 U/µl), dATP 0.2 mM final concentration and 1× reaction buffer in a final reaction volume of 25 µl. The reaction was incubated at 37°C for 30 min, followed by enzyme inactivation at 70°C for 10 min.

In the second approach, the 3′ terminal end was labeled with the adaptor A, a double stranded DNA sequence (5′GCCTCCCTCGCGCCATCAGNNNNNN3′ 3′CGGAGGGAGCGCGGTAGTC5′) by ligation. In this type of experiment, 5 µl of purified cDNA sample was mixed with T4 DNA ligase (1 Weiss U/µl), 1× reaction buffer, 35 pmol of adaptor A, in a final reaction volume of 25 µl. The reaction was incubated at 16°C overnight, following by enzyme inactivation at 70°C for 10 min. In most cases only one of these two approaches was utilized for determine the TSSs; however, there were some genes in which both approaches were utilized for TSSs determinations (table 1). All the reagents were purchased from Fermentas (St. Leon-Rot, Germany).

### 5) Linear or exponential amplification of tagged cDNA libraries

As a first approach we performed a linear PCR amplification in order to enrich the yield of the tagged cDNA complementary strand. Briefly, 20 pmol of RACE 1 primer (5′GACTCGAGTCGACATCGATTTTTTTTTTTTTTTTT3′) which harbors an adaptor sequence at its 5′ terminal end, was allowed to anneal to the poly A tract of the tagged cDNA, and used to linearly expand the library in an standard PCR amplification reaction under the following conditions: 1 cycle, 94°C for 10 min; 30 cycles of 94°C for 1 min, 45°C for 2 min, 72°C for 3 min, and finally one last extension cycle at 72°C for 5 min). In the second approach we performed exponential amplifications of the tagged cDNA libraries. Briefly, cDNA with labeled terminal ends was used as template and PCR primer adaptors A (5′GCCTCCCTCGCGCCATCAG3′) and B (5′GCCTTGCCAGCCCGCTC3′) for a PCR amplification reaction under the following conditions: 1 cycle, 94°C for 2 min; 35 cycles of 94°C for 1 min, 59°C for 45 sec and 72°C for 45 sec; 1 cycle, 72°C for 2 min. Both types of amplified samples were purified using the High Pure PCR product purification kit (Roche, Indianapolis, USA), accordingly to the manufacturers instructions. Figures 2, 3, 4 and S1 show the PAGE results of the amplified fragments.

### 6) Prime-specific PCR amplification and nucleotide sequence determination for TSSs identification

Both cDNA pools the linear and the exponentially amplified, were used as templates to selectively and individually amplify each selected gene using the high fidelity PCR system (Fast Start High Fidelity PCR System, Roche USA). This was achieved by using general adaptor primers (5′GACTCGAGTCGACATCGATT3′ and 5′GCCTCCCTCGCGCCATCAG3′) and a primer that specifically anneals to the cDNA complementary strand of the target gene. The nucleotide sequences of the specific primers of the analyzed genes are presented in table S1. A sample of the PCR product was analyzed by 8% native polyacrylamide gel electrophoresis and the band or bands, according to the number of detected TSSs, were then excised and purified from the gel. Finally, the nucleotide sequences of purified PCR products were determined using the same specific gene-primer employed for PCR amplification. Sequence reactions were run in an Applied Biosystem 3100 Genetic Analyzer/ABI PRISM device. The sequences were aligned with the *E. coli* K-12 genome of strain MG1655 and the specific TSSs, were identified as the first nucleotide immediately adjacent to the utilized adaptor. The nucleotide sequences obtained for the different genes and various TSSs are presented in figure S1.

### 7) Transcription start sites determination by pyrosequencing

Using random primers and the methodology described in the Mendoza *et al* (submitted to PLoS ONE), we were able to determine the TSSs of twelve promoters of glycolytic genes in wild type strain MG1665. These are presented in table 1. Briefly, total RNA from *E. coli strain* MG1665 grown in LB and minimal media at both 37° and 30°C was extracted and the rRNA was eliminated. For each sample a cDNA library was prepared utilizing the double adapter method (A and B previously described in 4). Using primers complementary to adaptors A and B, PCR amplicons were generated by Fast Start High Fidelity PCR System (Roche Applied Sciences, Indianapolis, USA) purified with MiniElute PCR purification Kit (Qiagen, Valencia, USA), and quantified using the NanoDrop spectrophotometer (NanoDrop Technologies, Wilmington, USA). At least 3 ug of cDNA were obtained for each sample. The quality of the DNA was evaluated by capillar electrophoresis using the Agilent Bioanalyser 2100 (Agilent Technologies, Palo Alto, USA). For pyrosequencing, samples were prepared according to 454 Roche GS FLX DNA Amplicon Library Preparation Kit user manual. Each amplicon mix was sequenced independently using the GS emPCR Kit II (454 Life Sciences Corporation, Branford, USA).

### 8) Real time PCR (RTPCR)

Previously reported real time PCR values for the analyzed genes, are presented in figure 1 [3], [30]. These values were utilized in this report, for comparative and discussion purposes.

### 9) In vitro transcription assays

Plasmid pSR a derivative of pBR322, was utilized for cloning the different DNA fragments carrying the *pgi*, *tpiA*, *pfkA*, *pykF*, *pykA*, *pdhR*, *acs* and *poxB* promoters that were analyzed for *in vitro* transcription. Standard techniques were used for recombinant DNA manipulation. Table S1 lists the nucleotide sequences of the primers used to amplify these promoters flanked by *Eco*RI and *Hind*III sites [24], [70], [74].

In order to overexpress and purify each sigma factor, *rpoS* and *rpoD* genes from *E. coli* were cloned into the expression vector pet28b+ (Novagen, Madison USA.) at the *Nde*I and *Eco*RI restriction sites. These constructions were introduced into strain ER2566 from IMPACT™ System from New England Biolabs (New York, USA). His-tag sigma factors were purified essentially by Ni^+2^ affinity chromatography as described by Morett *et al* [75]. *E. coli* RNA polymerase core was purchased from Epicenter Biotechnology (Madison, USA).

For the *in vitro* transcription experiments, the RNA polymerase holoenzyme was reconstituted by mixing the *E. coli* core enzyme with each purified σ^70^ and σ^38^ transcription factor at a molar ratio of 1∶5 and incubating at room temperature for 10 min in 100 µl protein storage buffer (50 mM Tris-HCl pH 8.0, 50% glycerol, 0.1 mM DTT, and 50 mM NaCl). The reaction mixture was prepared as follows: 4 µl of 5× transcription buffer (200 mM Tris-HCl, at pH 8.0, 5 mM DTT, 0.5 mg/ml BSA, 50 mM MgCl_2_ and 750 mM NaCl), 3 µl supercoiled plasmid DNA containing each promoter (pSR 0.1 µg/µl), and 7 µl H_2_O. 14 µl aliquots of the reaction mixture were mixed with 2 µl of the reconstituted RNAP holoenzyme, in a final reaction volume of 20 µl. Following a 15 min preincubation at 37°, the transcription reactions were initiated by the addition of 4 µl of 5× NTP mixture (1 mM each of ATP, GTP, and CTP, and 0.1 mM of UTP) containing 2 µCi of [α^32^P]-UTP. After incubation at room temperature for 15 min, reactions were stopped by the addition of 20 mM EDTA in formamide containing xylene cyanol and bromophenol blue. After heating at 70°C, samples were subjected to electrophoresis in 5% acrylamide sequencing gels. The RNA transcripts were visualized by exposure of the gel to a PhosphorImager screen (Amersham, Piscataway, USA) (Figures 6 and 2S).

### 10) Computer analysis and predictions of putative promoter regions

Putative σ^70^ and σ^38^ recognition sequences (table 1) were identified by visual inspection of the DNA sequences upstream the TSSs, and by multiple motif finding using the MEME and meta-MEME programs [http://meme.sdsc.edu/, http://metameme.sdsc.edu/) using the conserved elements in the promoter regions of *E. coli* for which the TSSs was experimentally determined. 297 σ^70^ and 51 σ^38^ promoter sequences were obtained. For the σ^70^ binding sites, signature of 10 nucleotides containing the −10 regions were detected in a window of 21 nucleotides upstream the TSSs. In the case of the σ^38^ recognition sequences, the −10 region of the consensus sequence for this sigma subunit (8 nucleotides) described by Weber *et al* [5], was utilized (table 1). Gene neighborhood was analyzed by the GeCont server [44].

## Supporting Information

Figure S1 Complete dataset of experiments carried out to map the 25 TSSs reported in this work corresponding to the 15 genes analyzed which produced TSSs. DNA sequence electropherograms, gels showing PCR products corresponding to the cDNA 3′ ends for each gene, DNA alignments of each sequence obtained with the genomic reference nucleotide sequence, and, if previously determined, the nucleotide sequence highlighting the reported TSS. Nucleotide sequences of the oligonucleotides utilized as specific primers for each gene for the 5′RACE experiments are described in table S1a. Subfigures a. to l. correspond to each gene analyzed and the order is the same as in table 1 and figures 2–4. Gels were loaded with PCR amplification reactions for each strain in order to separate different extension products, for the genes with more than one TSS, and to purify such DNA bands for nucleotide sequencing. Nucleotide sequences were determined for as many strains as possible and for the different DNA bands in the gels. For some genes very faint DNA bands were cut and mixed together to be able to get DNA sequence. As can be seen in several panels, the same TSSs were obtained from different strains. Figures 2–4 show the proposed and reported promoters for each one of the detected TSSs.
(7.58 MB PDF)

Figure S2 Detailed experimental strategy and complete set of results of in vitro transcription with purified E-sigma38 or E-sigma70. a; schematic representation of the promoter and terminator regions of the pSR plasmid used for in vitro transcription. This figure presents the way DNA fragments carrying each specific promoter region were cloned in plasmid pSR as EcoRI-HindIII fragments. As an example the nucleotide sequence of the EcoRI-HindIII fragment carrying the poxB promoter region, inserted into pSR, is presented. This vector has a rho-independent terminator, depicted in its stem and loop structure, such that the transcripts terminate at 88 nucleotides from the HindIII site marked by an asterisk. In the case of the poxB gene an RNA transcript of 88 nucleotides plus the size of the transcript from the +1(G) to the HindIII site (62 nucleotides), resulted in a transcript of 150 nucleotides (see also figure S2h). b; nucleotide sequence of the pgi promoter region inserted into pSR. As shown, two independent in vitro transcription experiments were performed for this gene. Transcripts originating from both putative overlapping dual pgiP1-P2 promoters were detected. c; nucleotide sequence of the pfkA promoter region inserted into pSR. As shown, two independent in vitro transcription experiments were performed for this gene. Transcripts originating from the putative overlapping dual pfkAP1-P2 promoters were detected. d; nucleotide sequence of the gapA promoter region inserted into pSR. As shown, an in vitro transcription experiment was performed for this gene. Transcripts originating from putative overlapping gapAP1-P1a dual promoters were detected. e; nucleotide sequence of the pykF promoter region inserted into pSR. As shown, an in vitro transcription experiment was performed for this gene. Transcripts originating from pykFP4-P5 and pykFP1-P2 putative dual promoters were detected. f; nucleotide sequence of the pykA promoter region inserted into pSR. As shown, two independent in vitro transcription experiments were performed for this gene. Transcripts originating from pykAP3-P4 and pykAP1-P2 putative overlapping dual promoters were detected. g; nucleotide sequence of the pdhR promoter region inserted into pSR. As shown, four independent in vitro transcription experiments were performed for this gene. Transcripts originating from putative pdhRP1-P2 overlapping dual promoters were detected. h; nucleotide sequence of the poxB promoter region inserted into pSR. As shown, five independent in vitro transcription experiments were performed for this gene. Transcripts originating from the putative overlapping dual poxBP1-P2 promoters were detected. i; nucleotide sequence of the acs promoter region inserted into pSR. As shown, one independent in vitro transcription experiment was performed for this gene. Transcripts originating from acsP2-P3 putative dual overlapping promoters were detected. RNA-I, which is transcribed by both sigma subunits from a promoter on plasmid pSR was used as internal control for in vitro transcription experiments (24).
(0.85 MB PDF)

Figure S3 The strategy for the construction of DNA fusions carrying the “closest to the ATG” promoters of selected glycolytic genes with a reported cat gene that confers chloramphenicol resistance (Cmr) is presented. Chromosomal DNA regions were constructed carrying these promoters of the selected genes together with the first 20 bp of the structural gene. Nucleotide sequences of the oligonucleotides that were utilized for amplifying the selected specific DNA fragments are presented in table S1. Each experiment utilized one forward (Fw) and one reverse (Rv) fragments to produce the PCR amplified products. Selected amplified DNA fragments were cloned into the SmaI site of plasmid pKK238; this site is in front of the cat reporter gene of this plasmid. The sizes of recombinant molecules in the inserts, were assayed by digesting plasmids with PstI. As can be seen in the gel, we were able to clone all the “promoter or dual promoters closest to the ATG initiation codon” of the analyzed genes. DNA fragments carrying these promoters: pgiP1-P2 (PCR size of 171 bp and 1127 bp after PstI digestion, respectively), pfkAP3-P4 (140 and 1096), tpiAP1-P2 (117 and 1073), enoP4-P5 (105 and 1064), and pgkP2 (108 and 1064), are presented in the figure and in the gel. The agarose gel exhibits PstI digested DNA from the different recombination molecules, including the vector digested with the same enzyme that generates a band of 953 bp (column B). All cloned DNA fragments had higher molecular weights than 953 bp after PstI digestion, indicating that the insert is present. The gel also includes molecular weight markers (column A). The nucleotide sequences of the inserted fragments confirmed the existence of the cloned DNA fragments (data not shown).
(1.49 MB PPT)

Table S1 Table S1 lists the oligonucleotides utilized in this work. Section A shows the specific primers used in the modified 5′RACE methodology for DNA sequencing the TSS (figure S1). Section B lists the oligonucleotides employed for the construction of DNA fusions carrying different promoters of several genes with the cat reporter gene (figure S3). Section C shows the oligonucleotides used in the in vitro transcription experiments. DNA fragments carrying the promoters of the selected genes were amplified using the two oligonucleotides that carry EcoRI and HindIII sites at their ends. After PCR, the amplified fragments were cloned in the PSR plasmid for the in vitro transcription experiments (for details see Materials and methods and figures 6 and S2). The table shows the nucleotide sequences of the amplified fragment for each gene (in lower case) in which the TSSs are underlined.
(0.04 MB XLS)
