# Supplementary material for: Transcription Analysis of Central Metabolism Genes in Escherichia coli. Possible Roles of σ38 in Their Expression, as a Response to Carbon Limitation
Source: PLoS One. 2009 Oct 19;4(10):e7466. doi: 10.1371/journal.pone.0007466 (PMC2759082; doi:10.1371/journal.pone.0007466)
Supplement: Figure S1 — Complete dataset of experiments carried out to map the 25 TSSs reported in this work corresponding to the 15 genes analyzed which produced TSSs. DNA sequence electropherograms, gels showing PCR products corresponding to the cDNA 3′ ends for each gene, DNA alignments of each sequence obtained with the genomic reference nucleotide sequence, and, if previously determined, the nucleotide sequence highlighting the reported TSS. Nucleotide sequences of the oligonucleotides utilized as specific primers for each gene for the 5′RACE experiments are described in table S1a. Subfigures a. to l. correspond to each gene analyzed and the order is the same as in table 1 and figures 2– 4. Gels were loaded with PCR amplification reactions for each strain in order to separate different extension products, for the genes with more than one TSS, and to purify such DNA bands for nucleotide sequencing. Nucleotide sequences were determined for as many strains as possible and for the different DNA bands in the gels. For some genes very faint DNA bands were cut and mixed together to be able to get DNA sequence. As can be seen in several panels, the same TSSs were obtained from different strains. Figures 2– 4 show the proposed and reported promoters for each one of the detected TSSs. (7.58 MB PDF) [file pone.0007466.s001.pdf]

Figure S1

a. *glk* Glucokinase

One of the detected TSSs of the *glk* gene (*glk*P1-P2) is different from one previously reported by one nucleotide (36).

Name:	glkp
+1:	2507483
Sequence:	ttacagggagcctgcctttccggcgttgttgttatgcccccaggtatttacagtgtgagaAagaattattttgactttagc

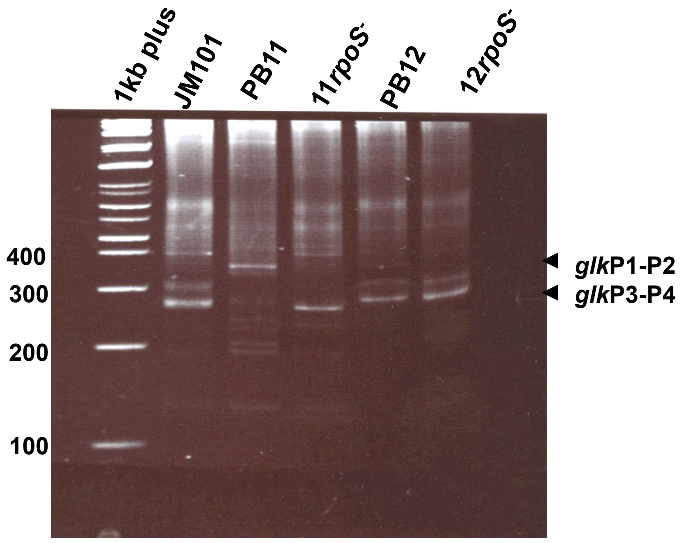

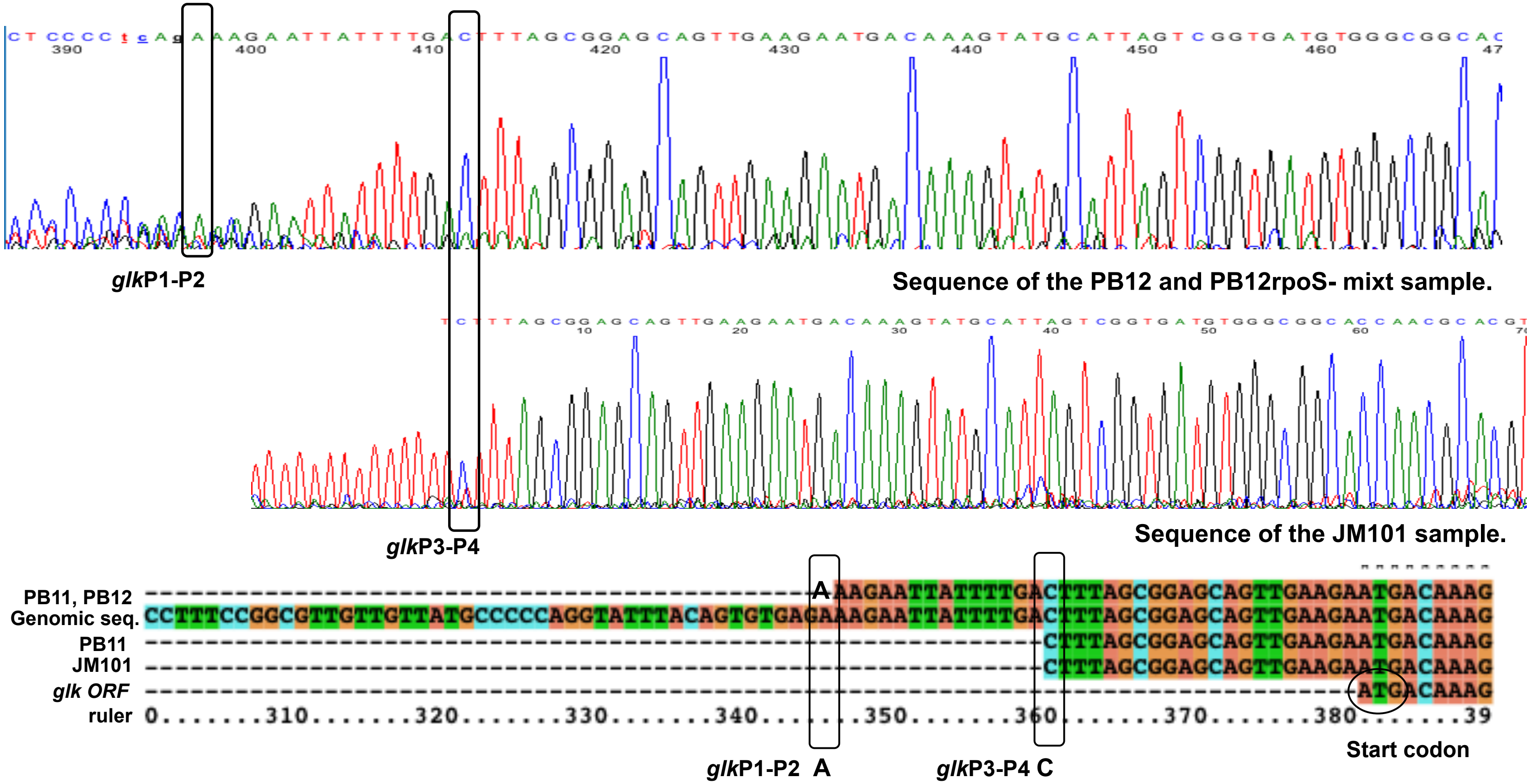

a. *glk* Glucokinase

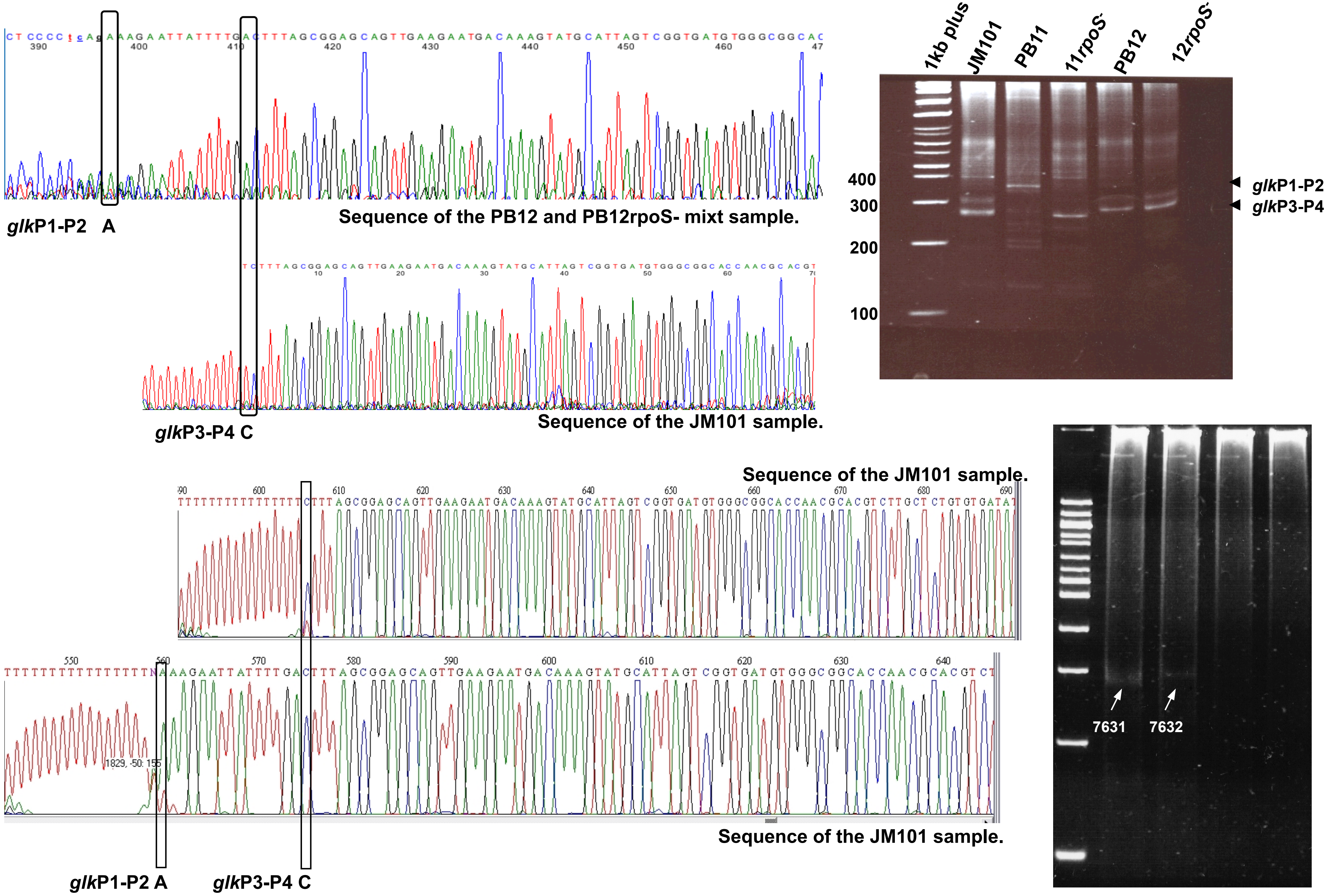

b. *pgi* Glucosephosphate isomerase

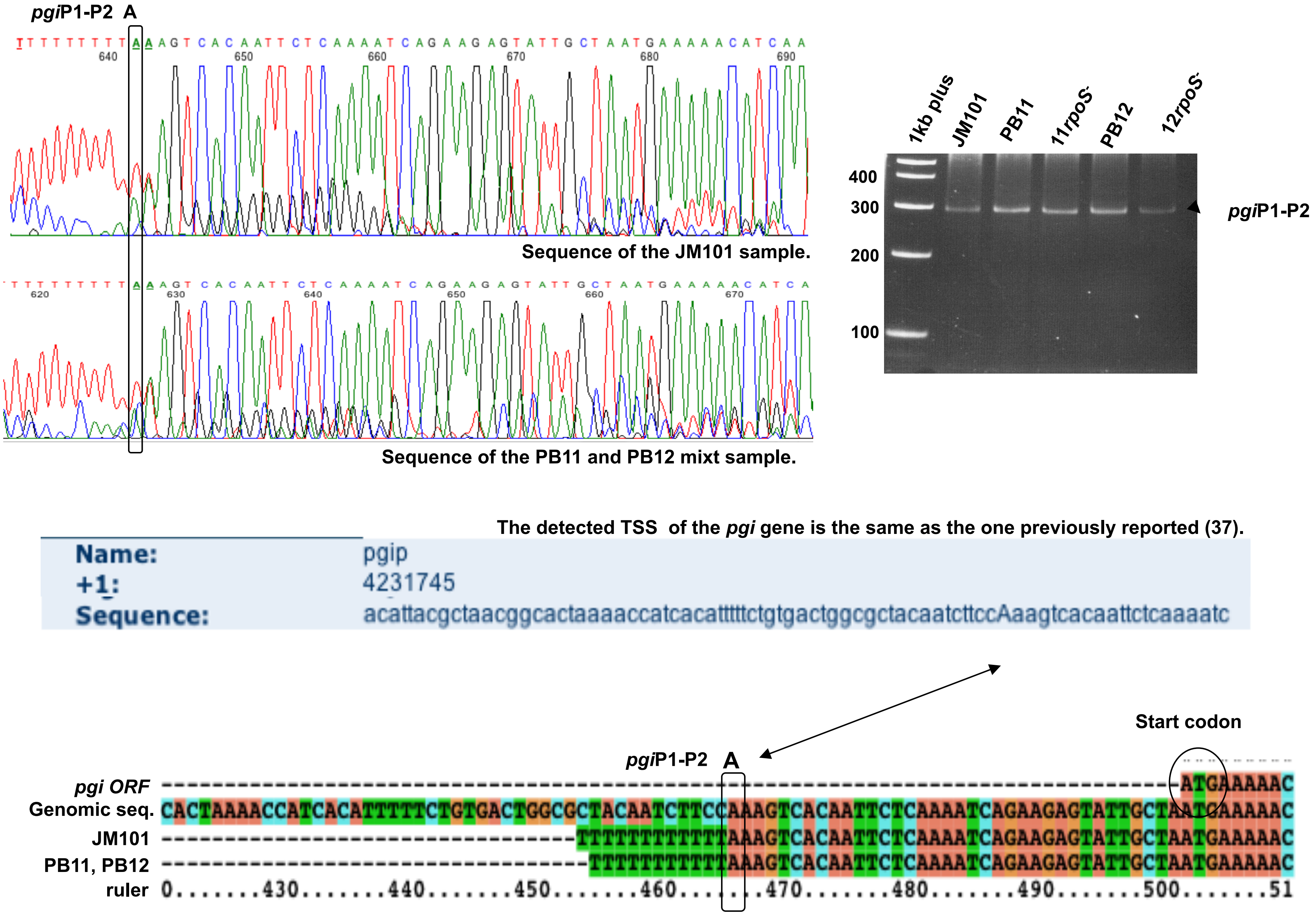

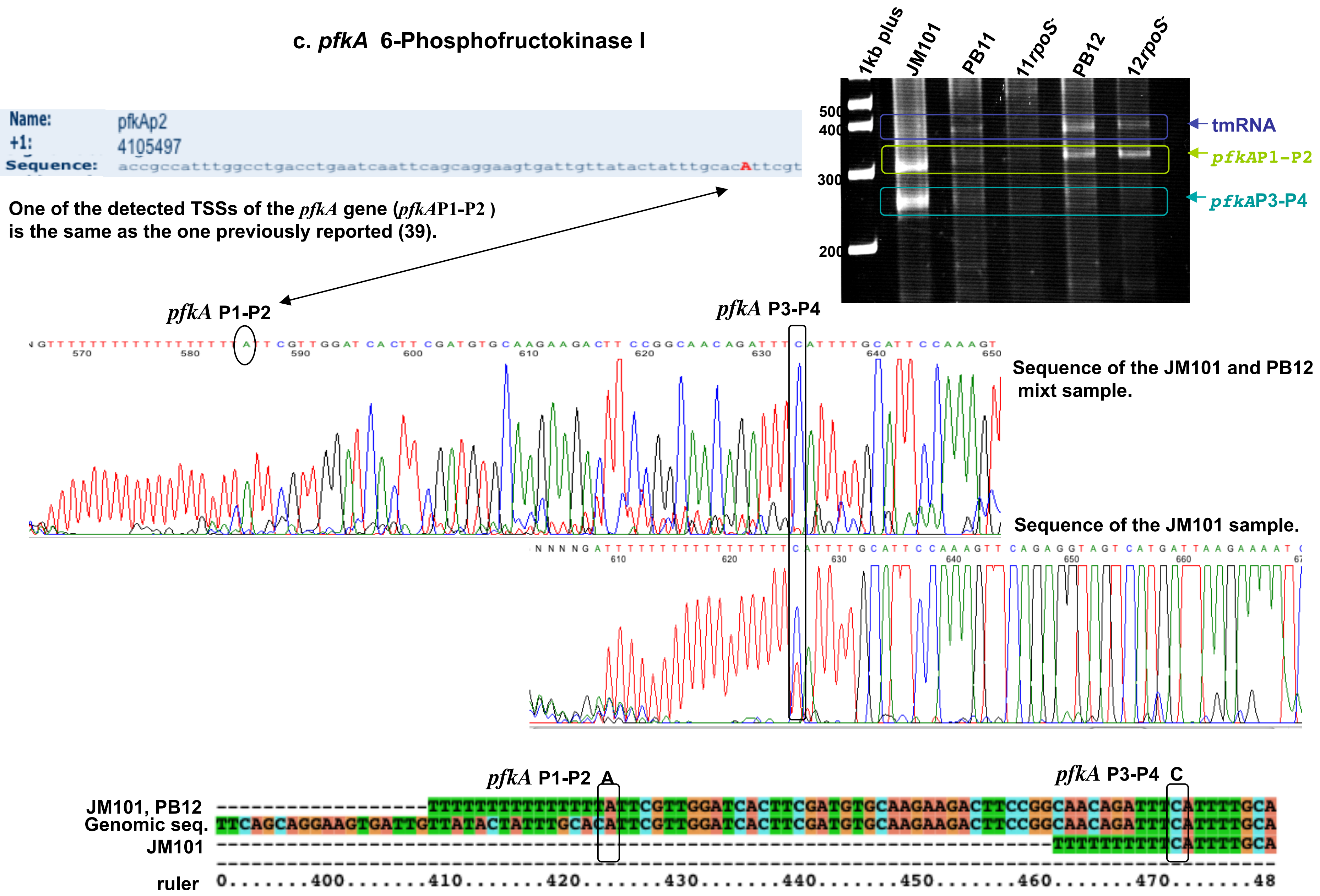
c. pfkA 6-Phosphofructokinase I
Name: pfkAp2
+1: 4105497
Sequence: accgccatttggcctgacctgaatcaattcagcaggaagtgattgttatactatttgcacAttcgt
One of the detected TSSs of the pfkA gene (pfkAP1-P2) is the same as the one previously reported (39).
1kb plus
JM101
PB11
11rpoS-
PB12
12rpoS-
500
400
300
200
tmRNA
pfkAP1-P2
pfkAP3-P4
pfkA P1-P2
pfkA P3-P4
Sequence of the JM101 and PB12 mixt sample.
Sequence of the JM101 sample.
pfkA P1-P2 A
pfkA P3-P4 C
JM101, PB12 ------------------TTTTTTTTTTTTTTTATTCGTTGGATCACTTCGATGTGCAAGAAGACTTCCGGCAACAGATTTCATTTTGCA
Genomic seq. TTCAGCAGGAAGTGATTGTTATACTATTTGCACATTCGTTGGATCACTTCGATGTGCAAGAAGACTTCCGGCAACAGATTTCATTTTGCA
JM101 --TTTTTTTTTCATTTTGCA
ruler 0.......400.......410.......420.......430.......440.......450.......460.......470.......48

c. *pfkA* 6-phosphofructokinase I

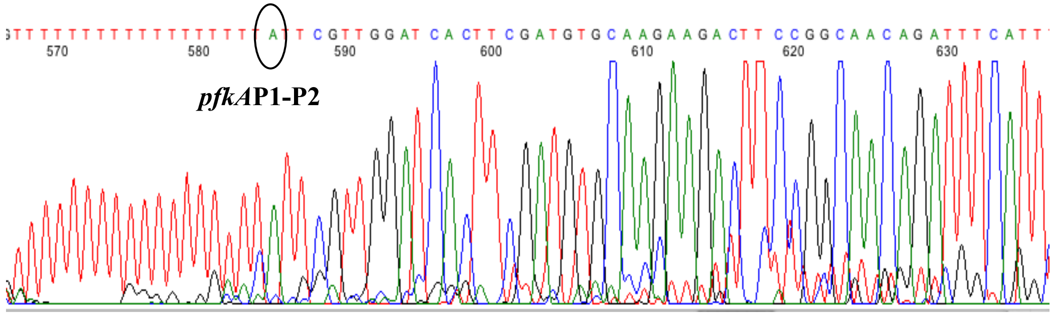

Sequence of the JM101 and PB12 mixt sample.

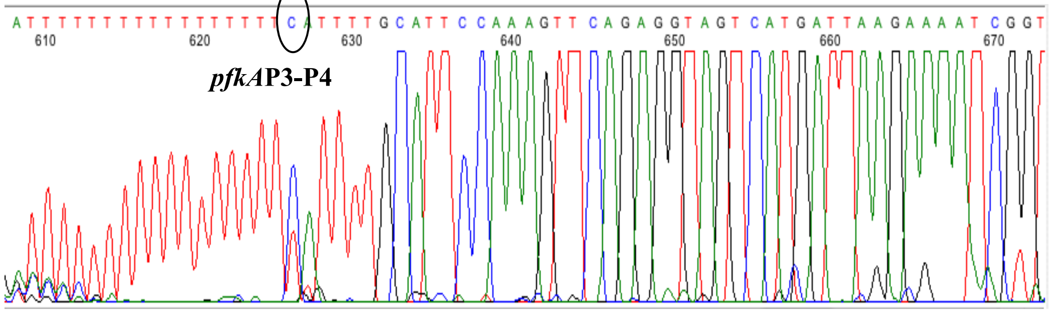

Sequence of the JM101 sample.

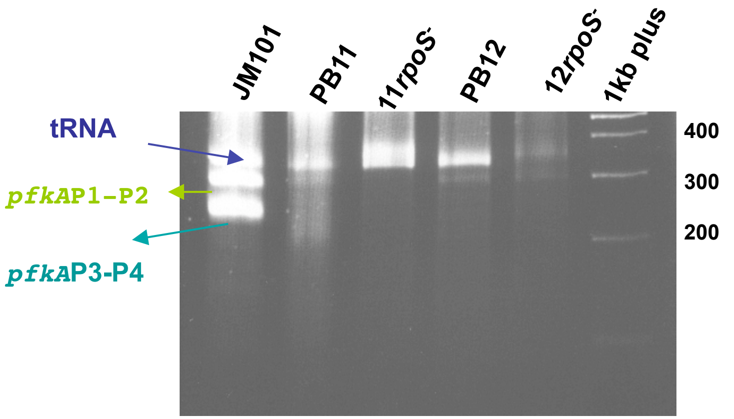

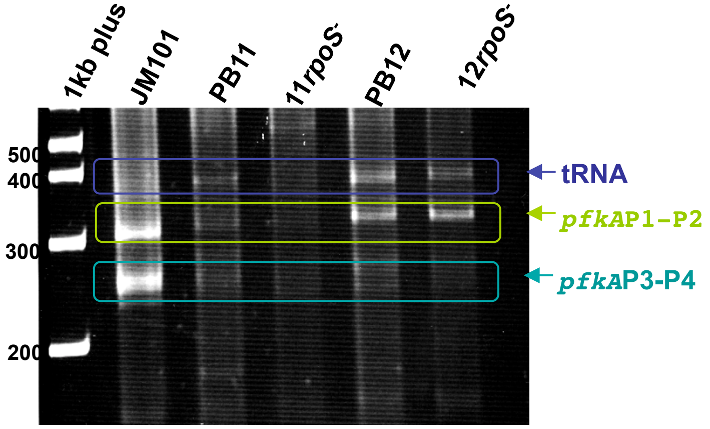

Two independent experiments were performed for this gene.

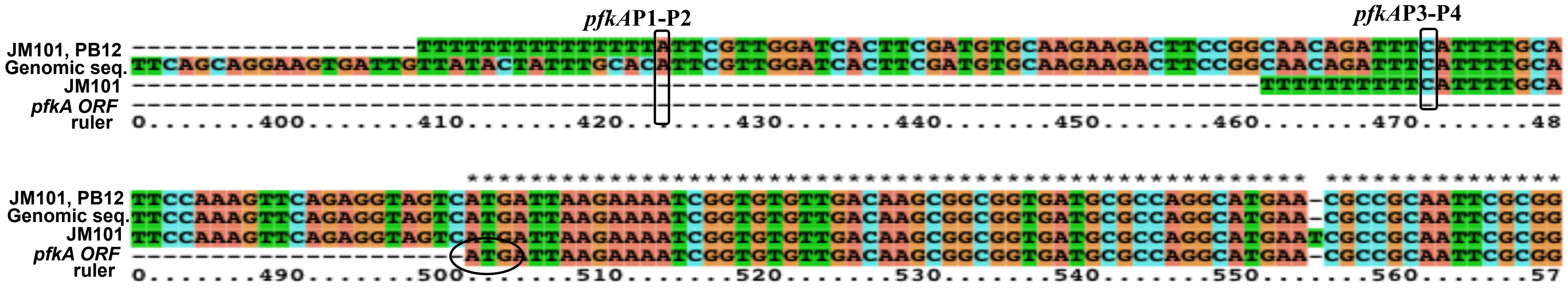

d. *tpiA* Triosephosphate isomerase

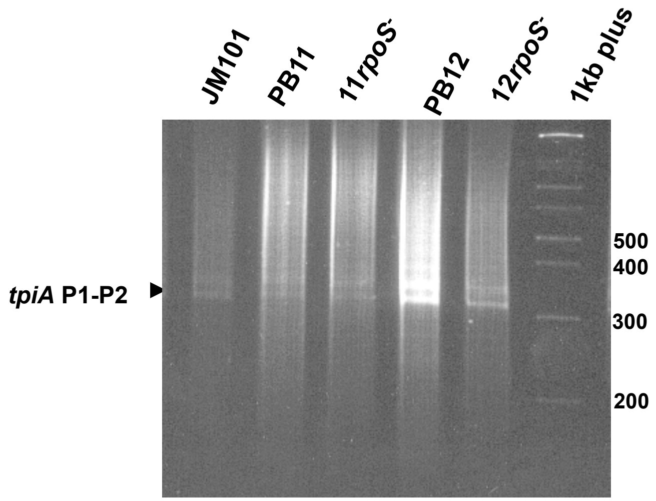

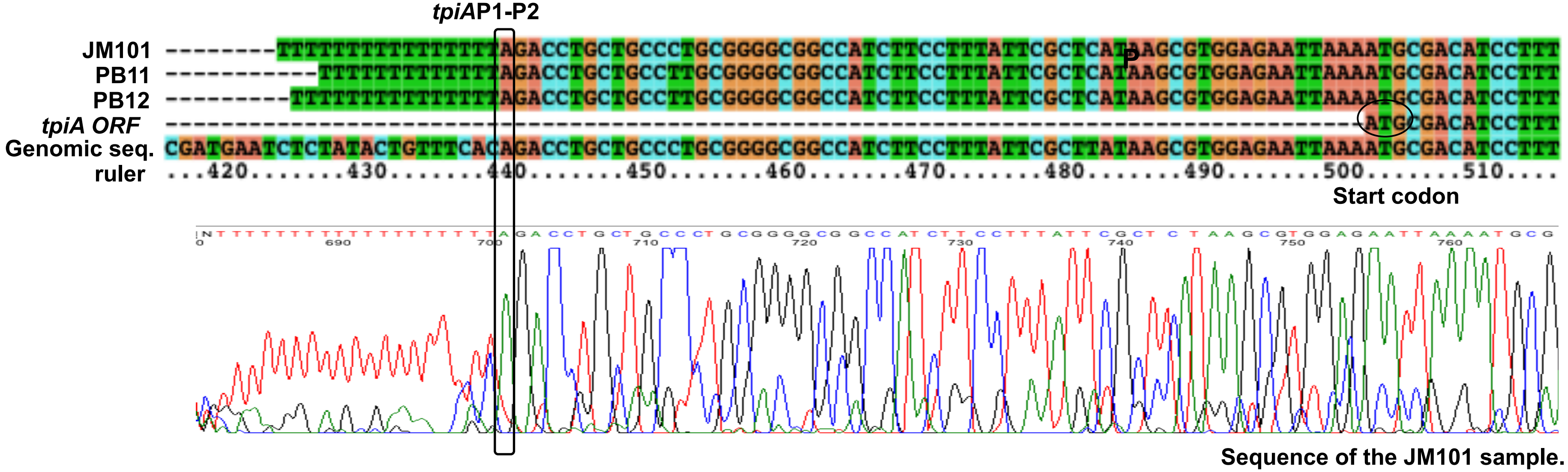

Sequence of the JM101 sample.

A previously reported TSS of the *tpiA* gene was not detected (40).

Name:	tpiAp
+1:	4109547
Sequence:	tctatactgtttcacagacctgctgccctgcggggcggccatcttcctttattcgcttatAagcgtggagaattaaaa

d. *tpiA* Triosephosphate isomerase

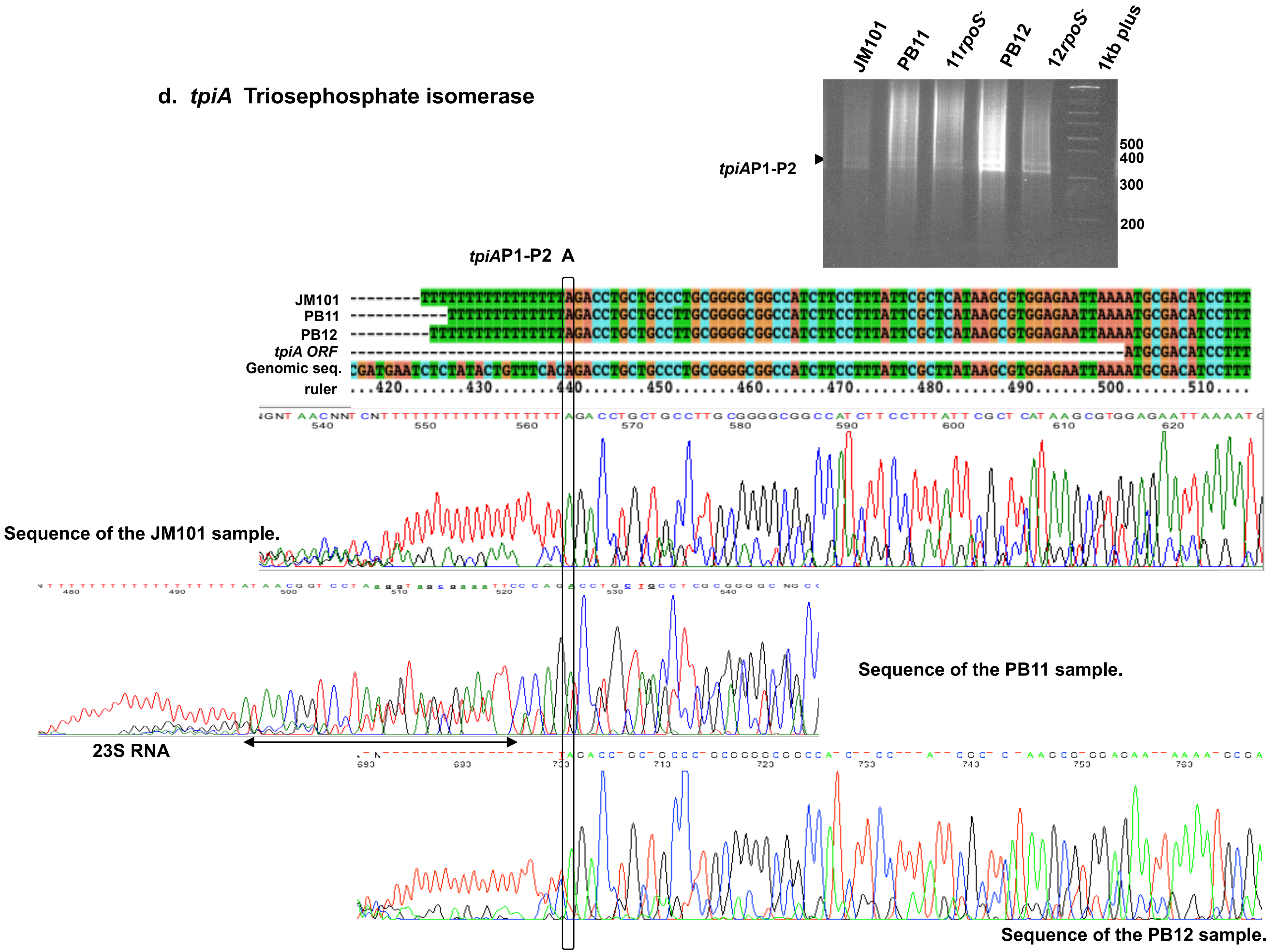

e. *gapA* Glyceraldehyde 3-phosphate dehydrogenase A

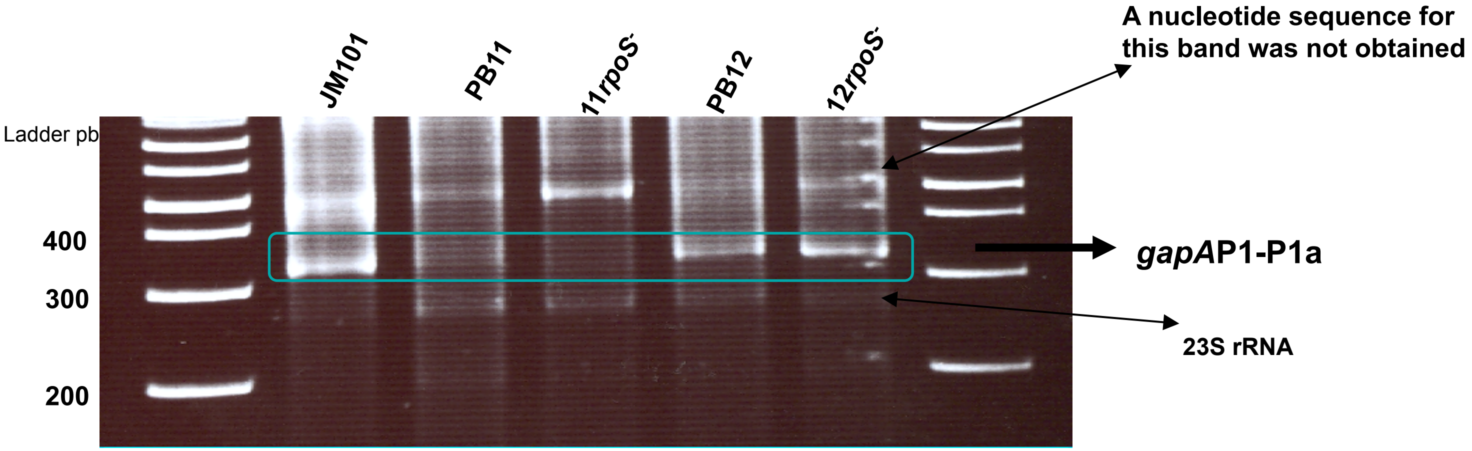

The detected TSS of the *gapA* gene is the same as one previously reported (41,43).

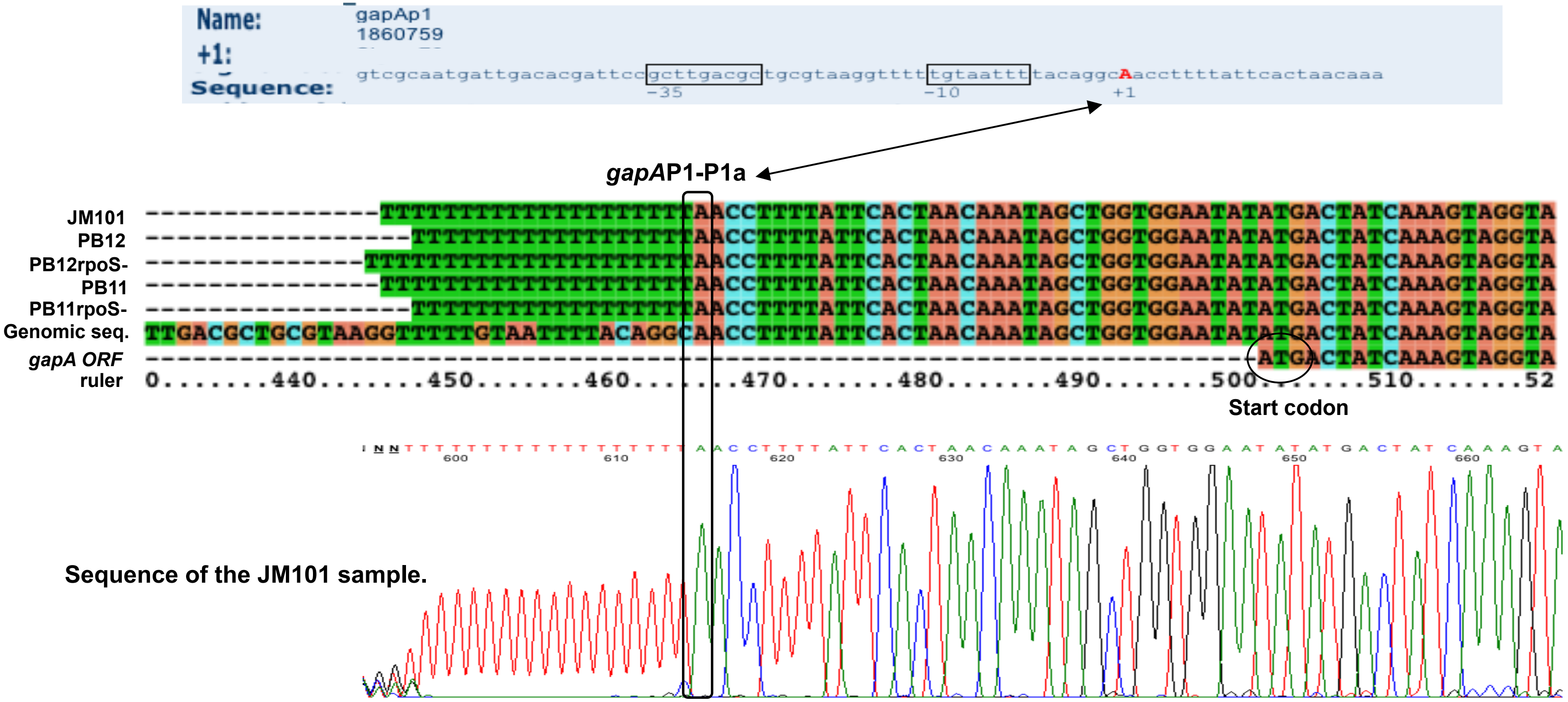

e. *gapA* Glyceraldehyde 3-phosphate dehydrogenase A

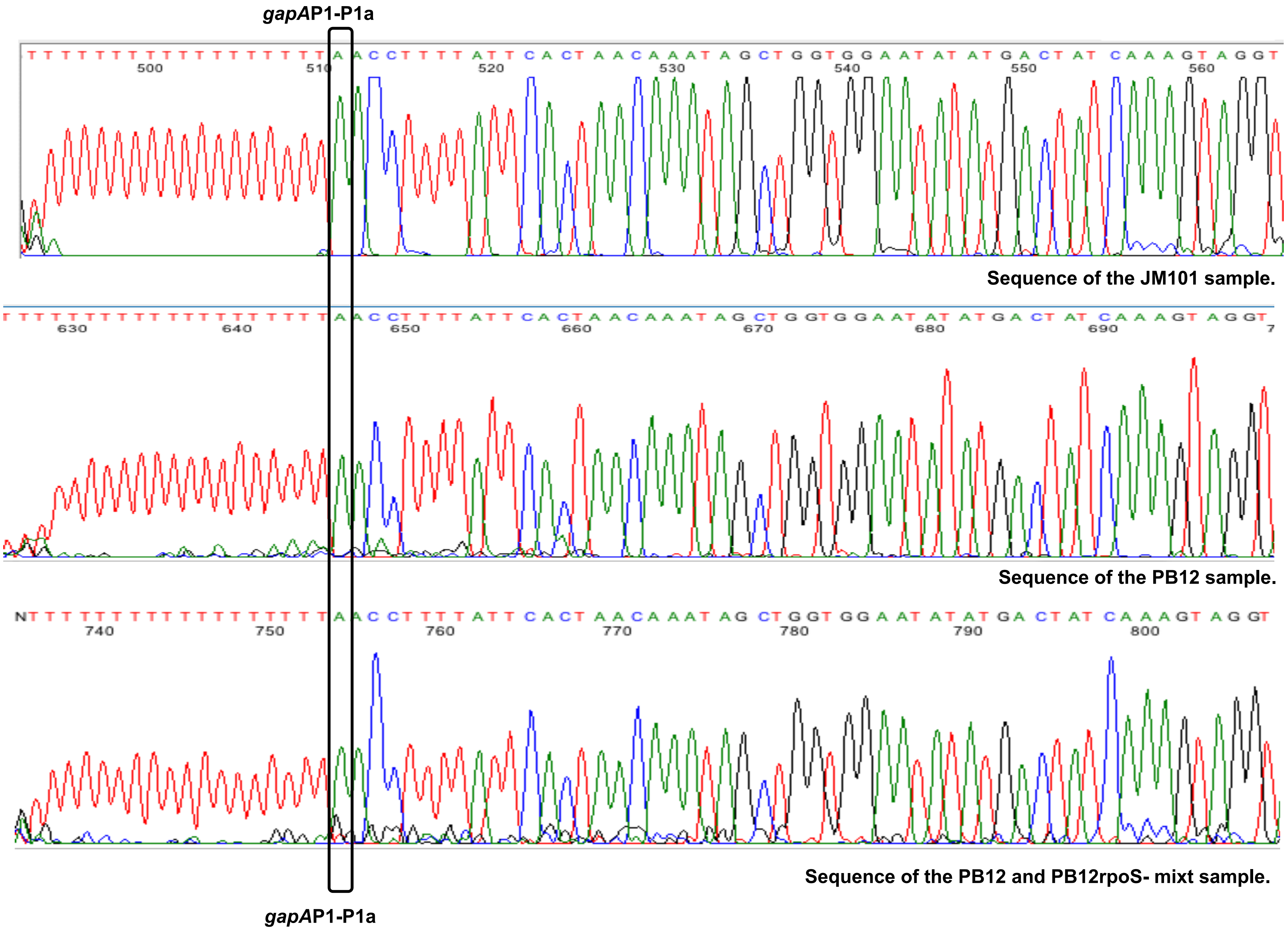

f-1. *epd* D-erythrose 4-phosphate dehydrogenase

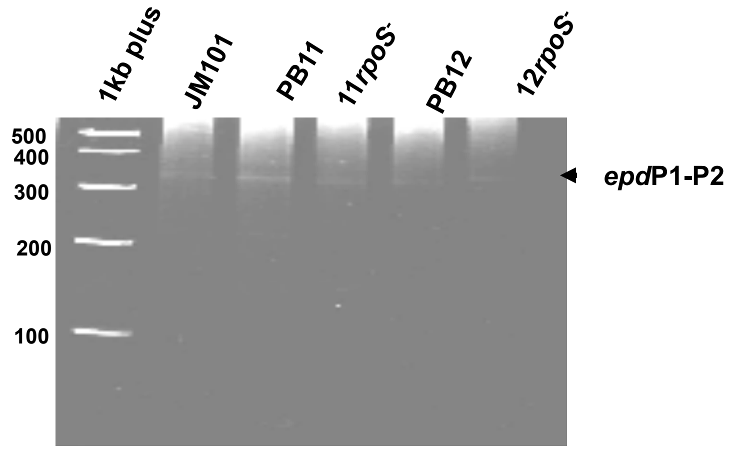

The detected TSS of the *epd* gene is the same as the one previously reported (45).

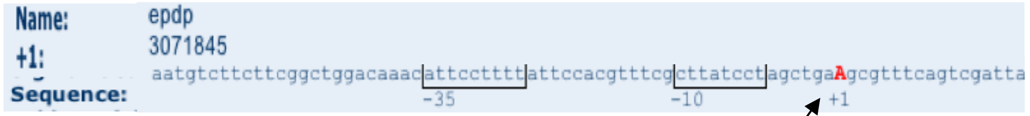

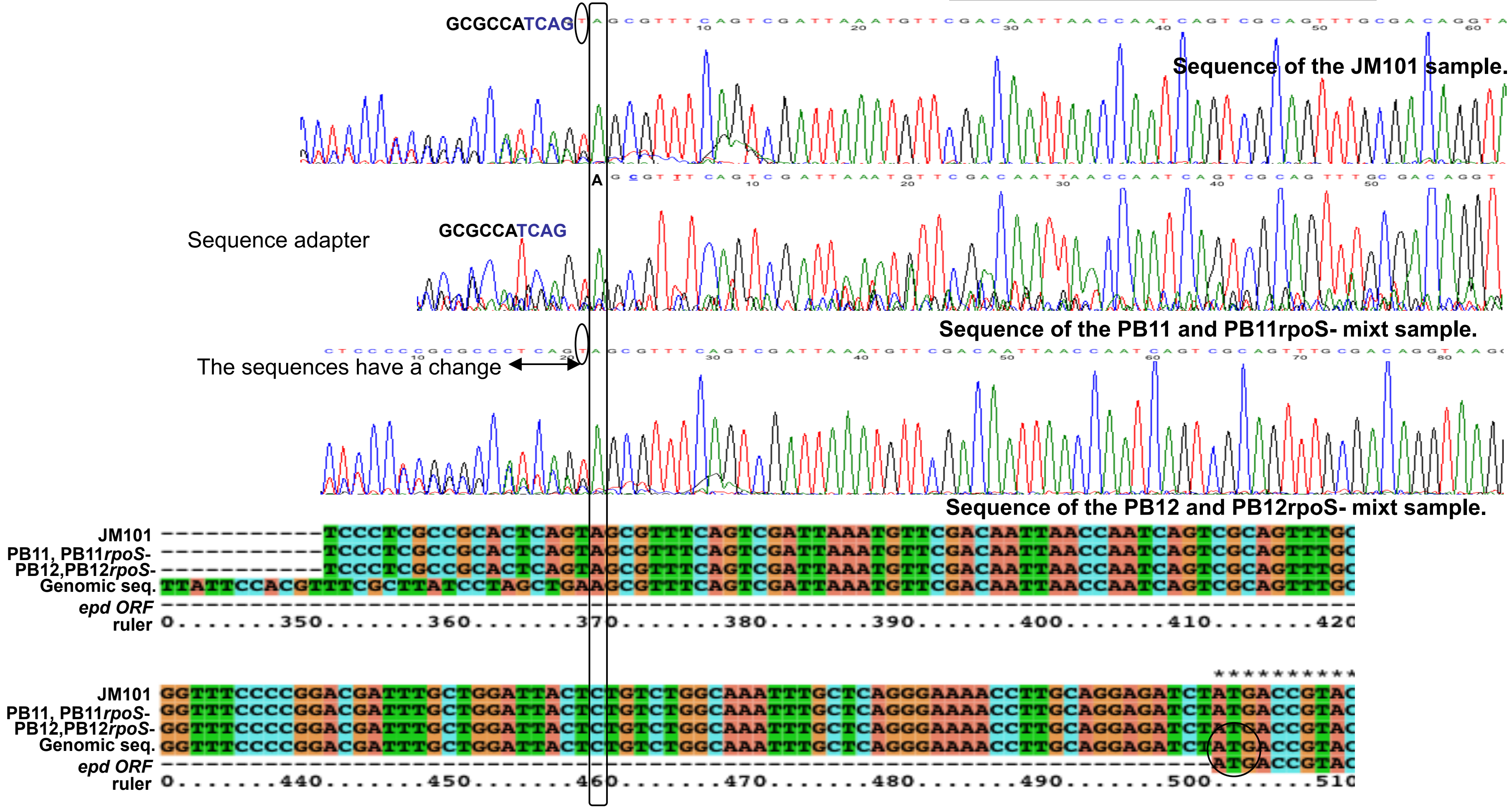

f-2. *pgk,* Phosphoglycerate kinase.

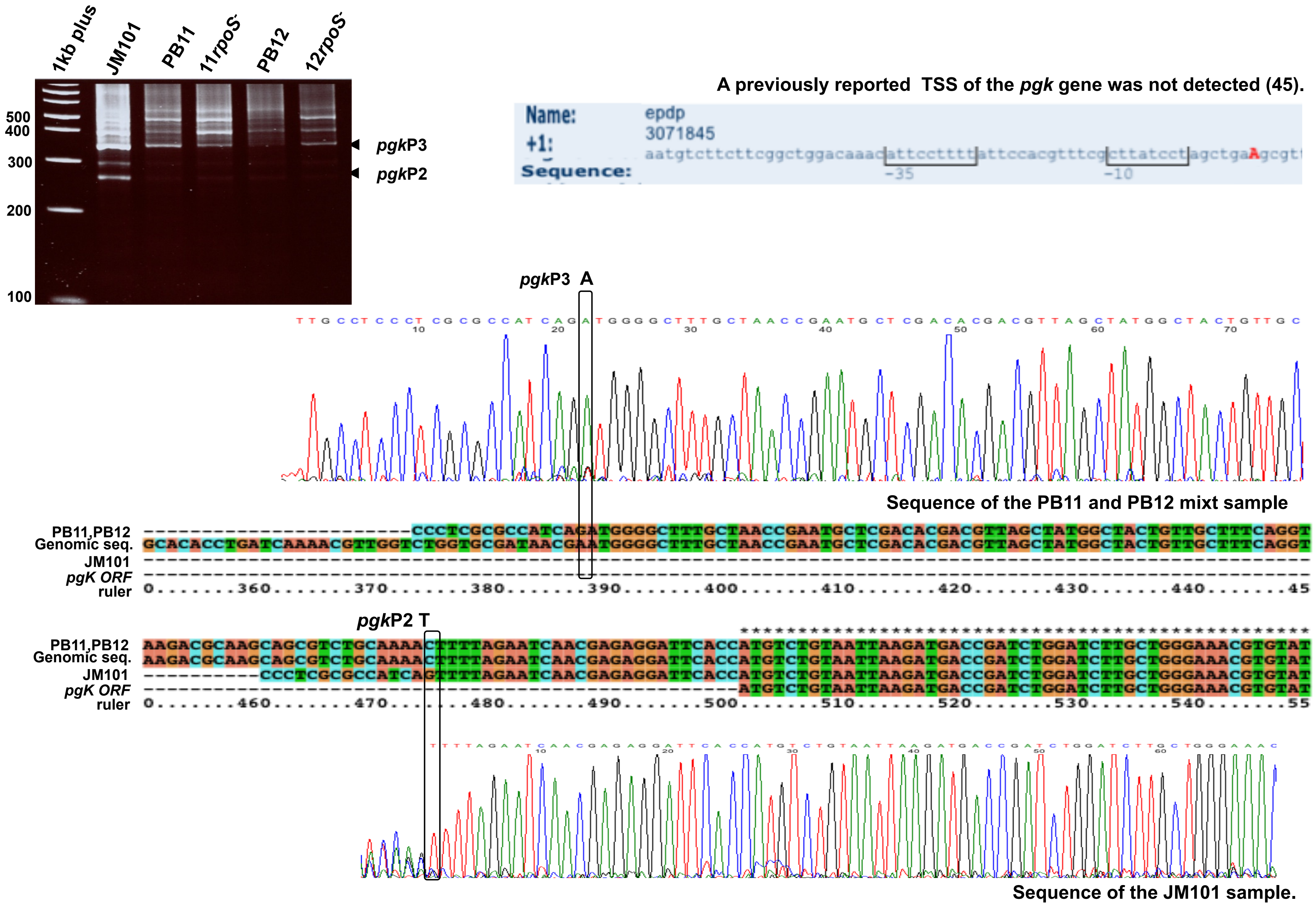

g. *gpmA* Phosphoglyceromutase A

One of the detected TSSs of the *gpmA* gene is different from one previously inferred by two nucleotides (46).

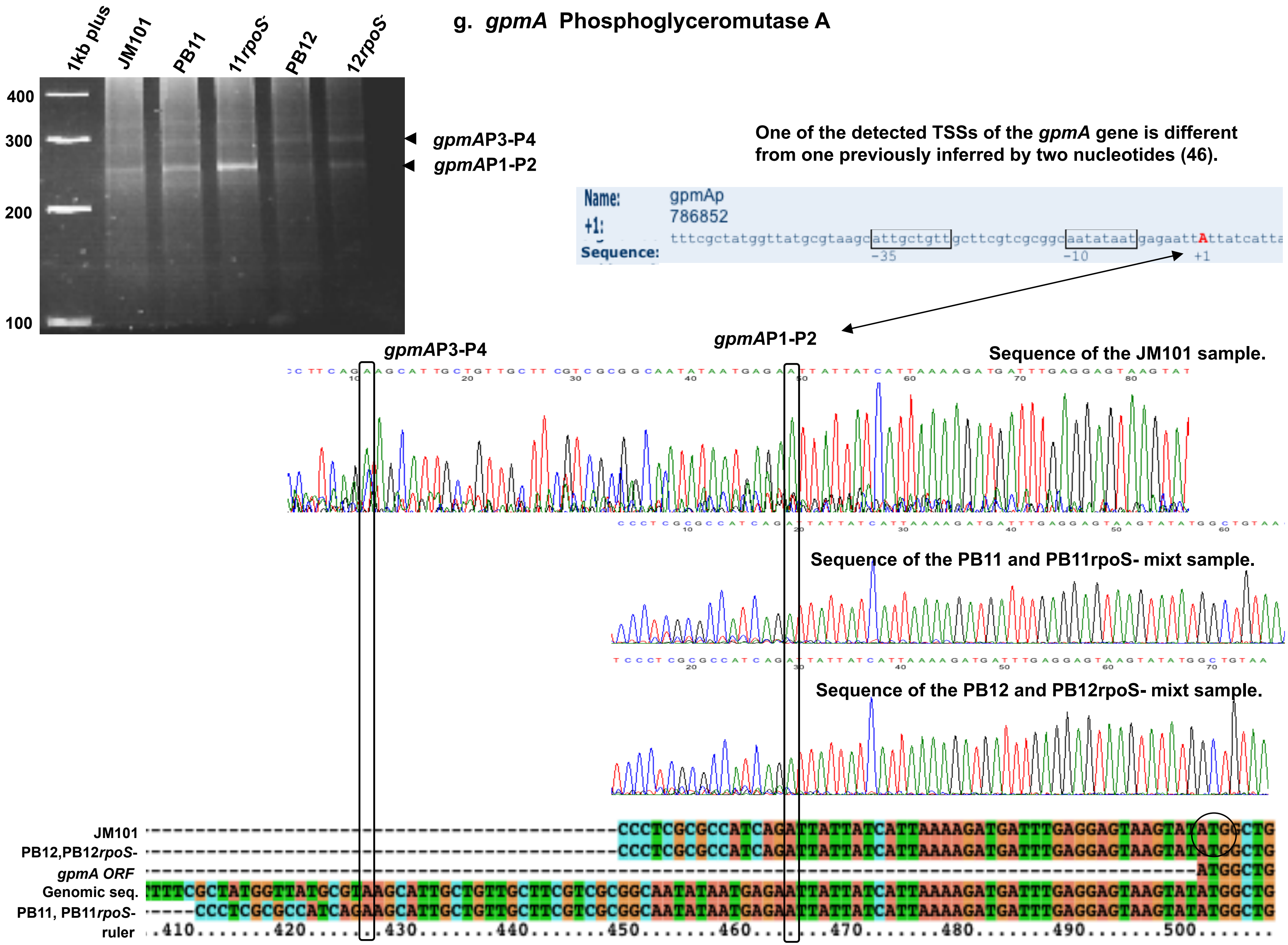

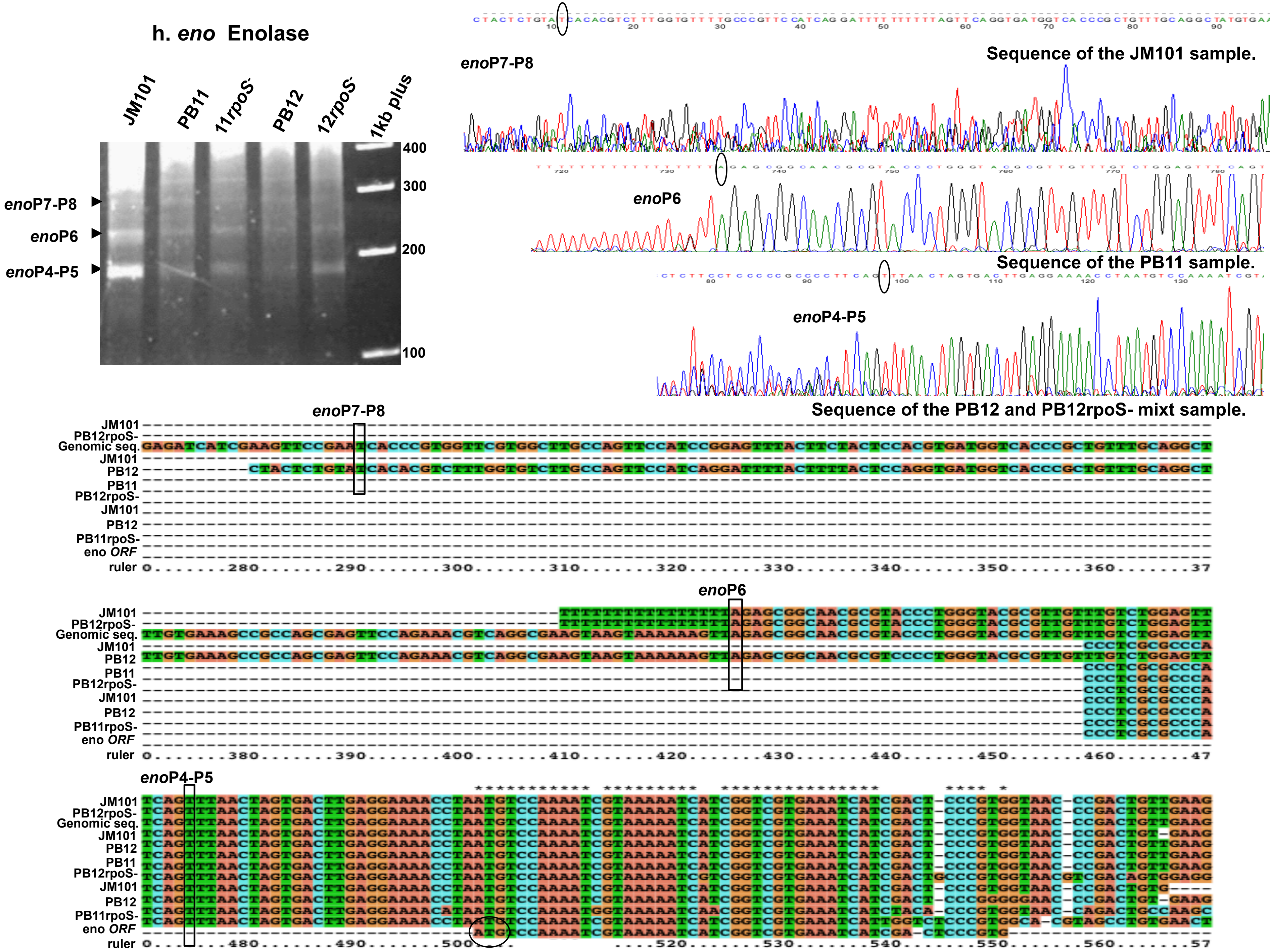

h. eno Enolase
JM101
PB11
11rpoS-
PB12
12rpoS-
1kb plus
400
300
200
100
enoP7-P8
enoP6
enoP4-P5
Sequence of the JM101 sample.
Sequence of the PB11 sample.
Sequence of the PB12 and PB12rpoS- mixt sample.
JM101
PB12rpoS-
Genomic seq.
PB12
PB11
PB11rpoS-
eno ORF
ruler

h.1. *eno* Enolase

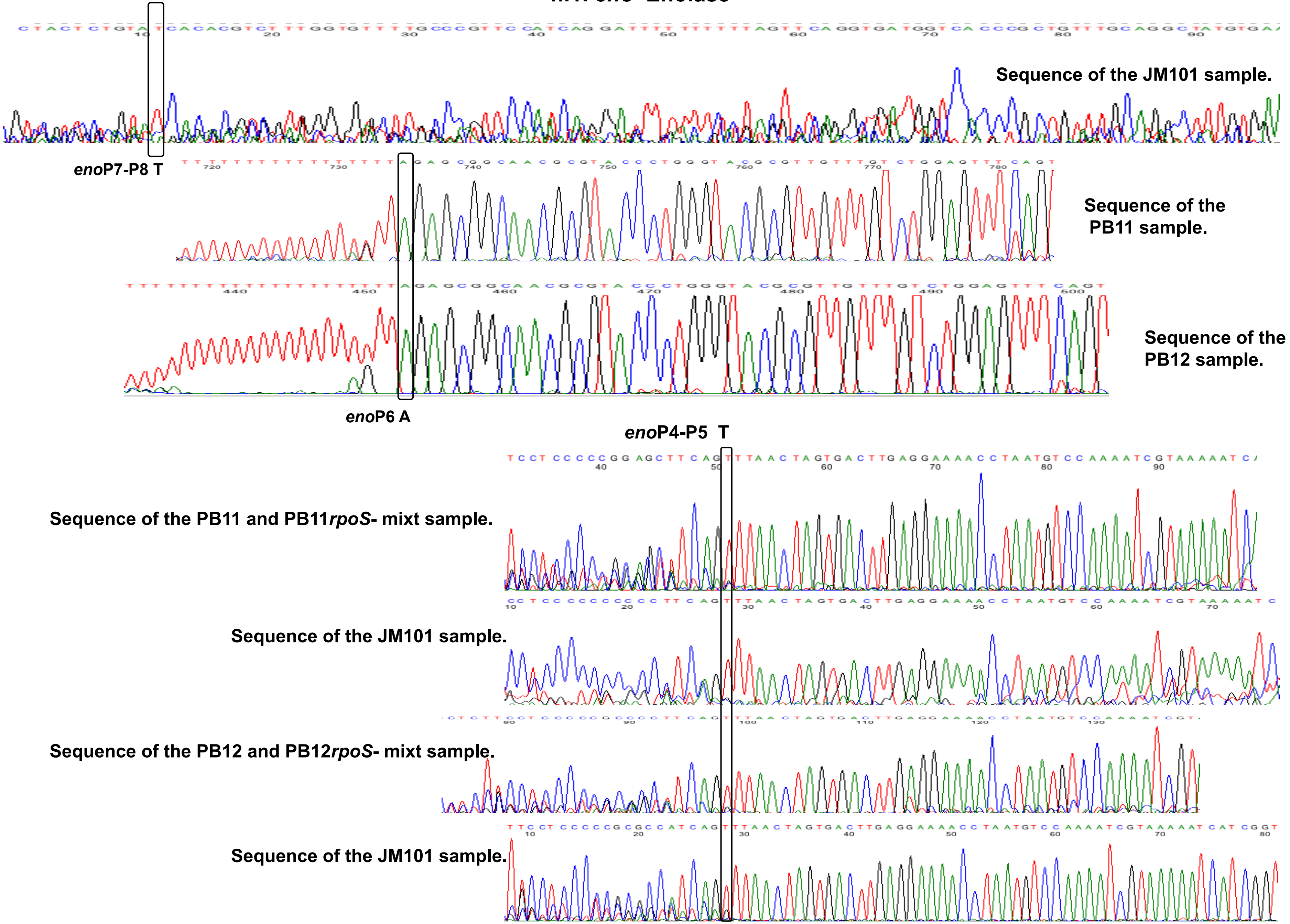

h.2 *eno* Enolase

Four independent experiments for this gene produced the same results.

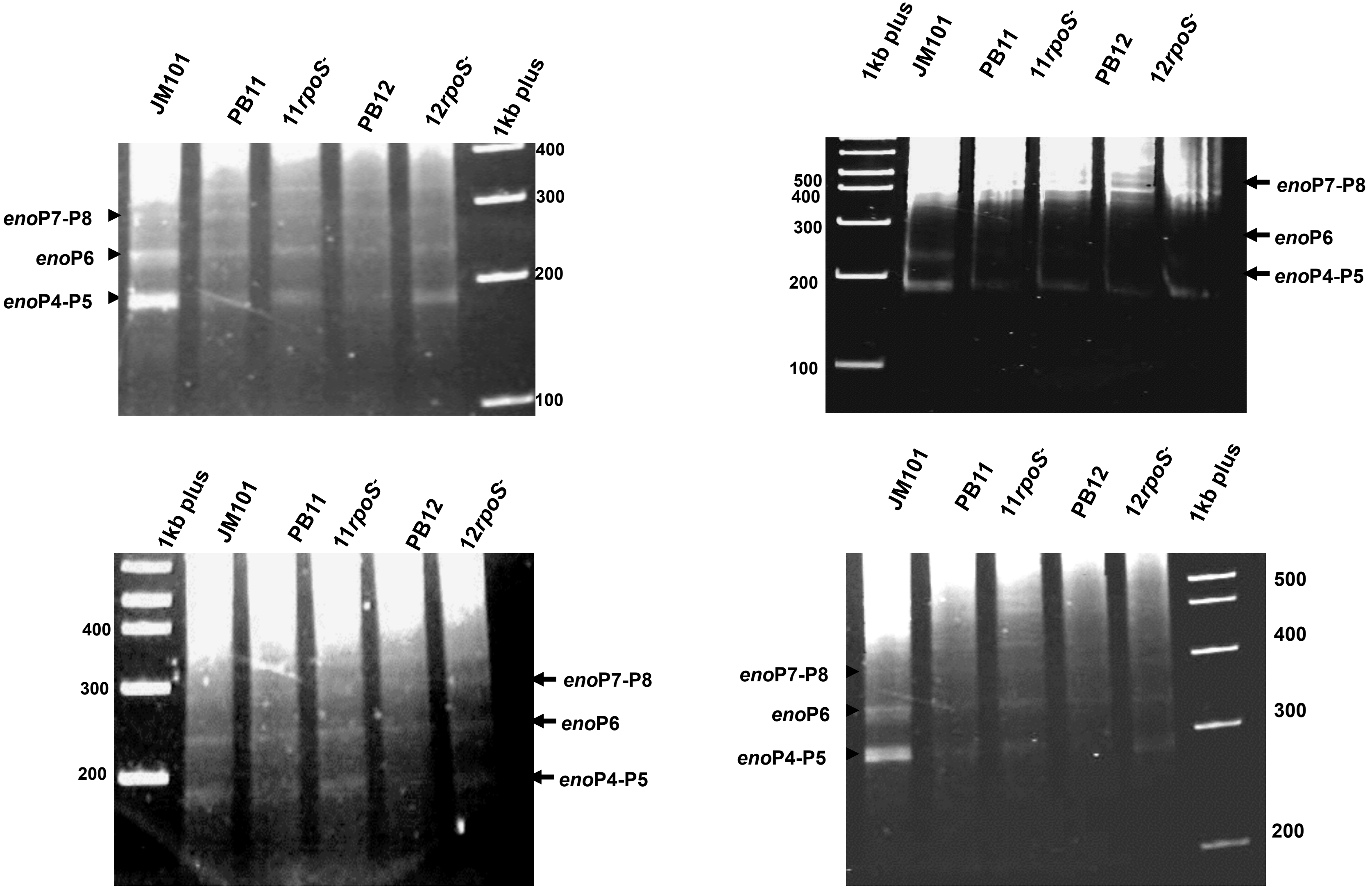

Three previously reported TSSs for this gene with long untranslated regions were not detected (47).

i-1 *pykF* Pyruvate kinase F

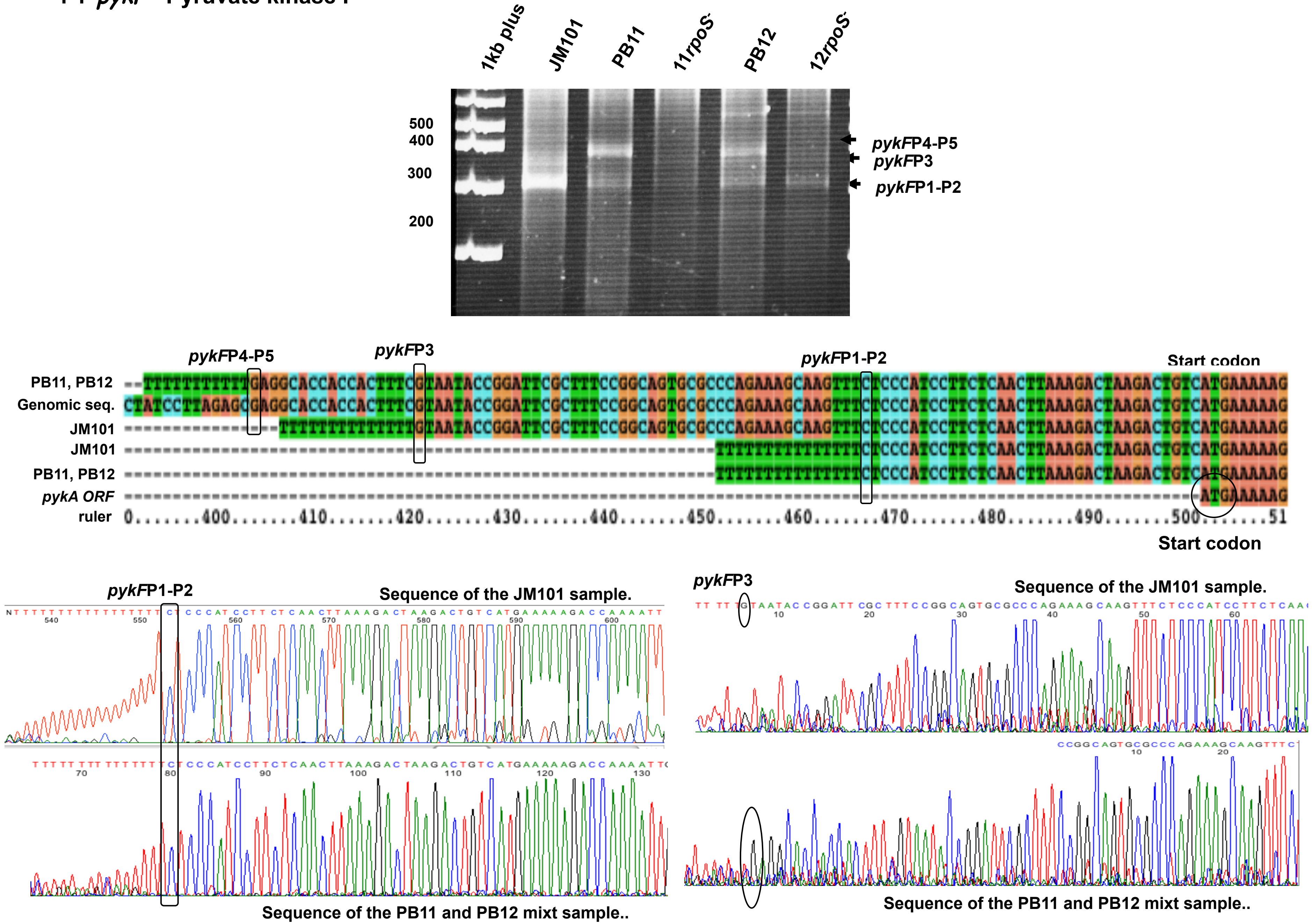

i-2 *pykA* Pyruvate kinase A

Two independent experiments produced the same results.

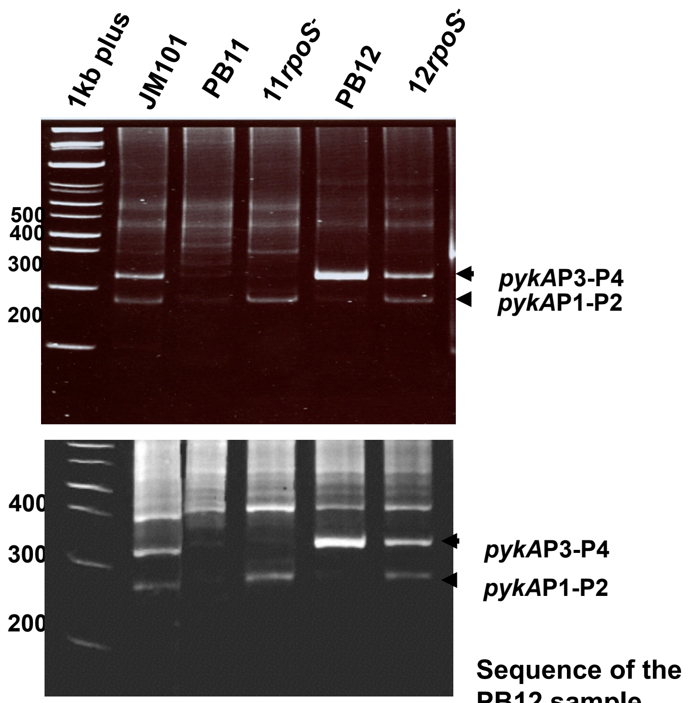

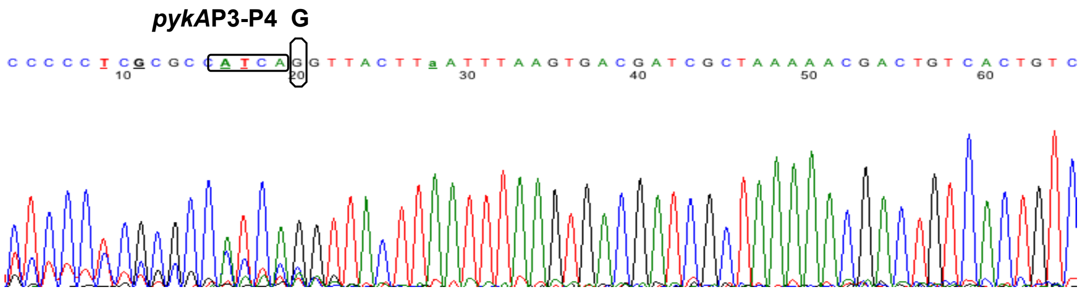

Sequence of the JM101 sample.

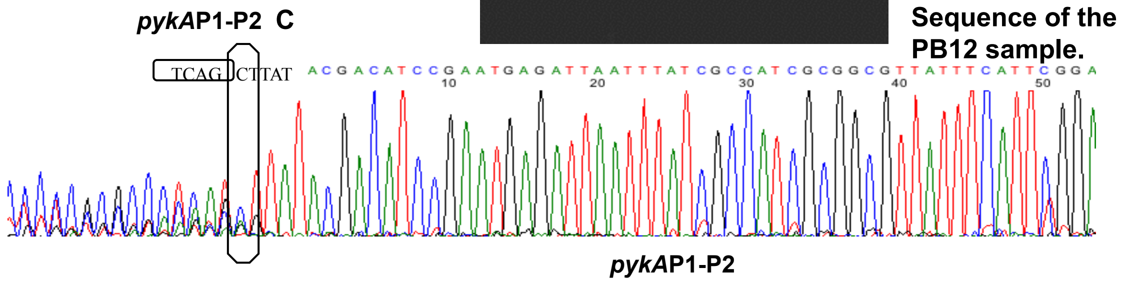

Sequence of the PB12 sample.

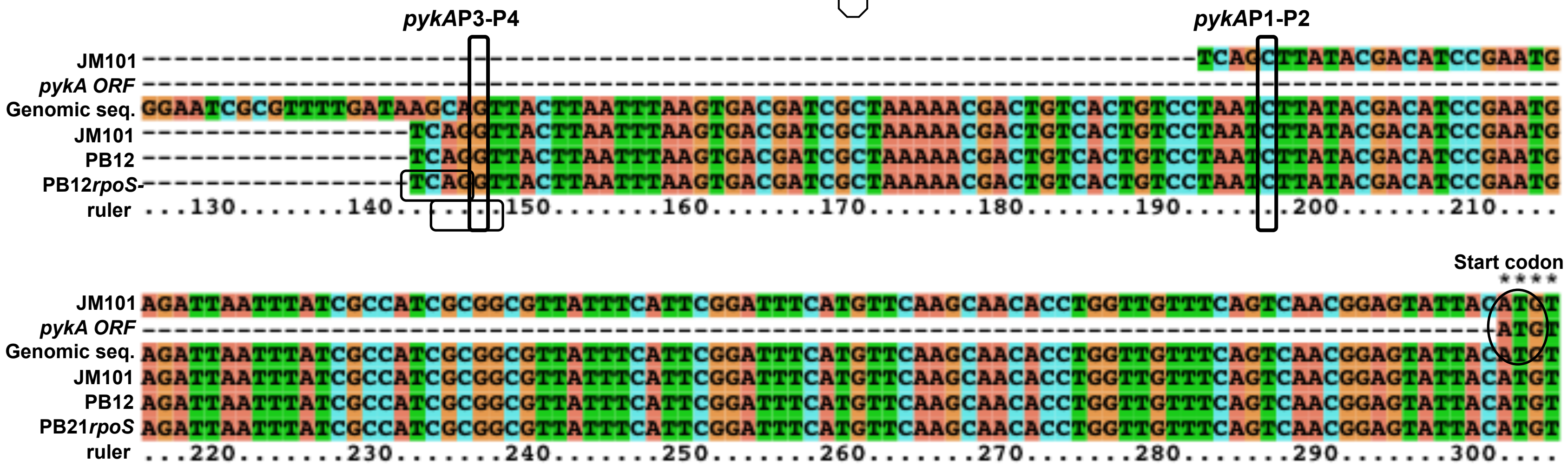

i-2 *pykA* Pyruvate kinase A

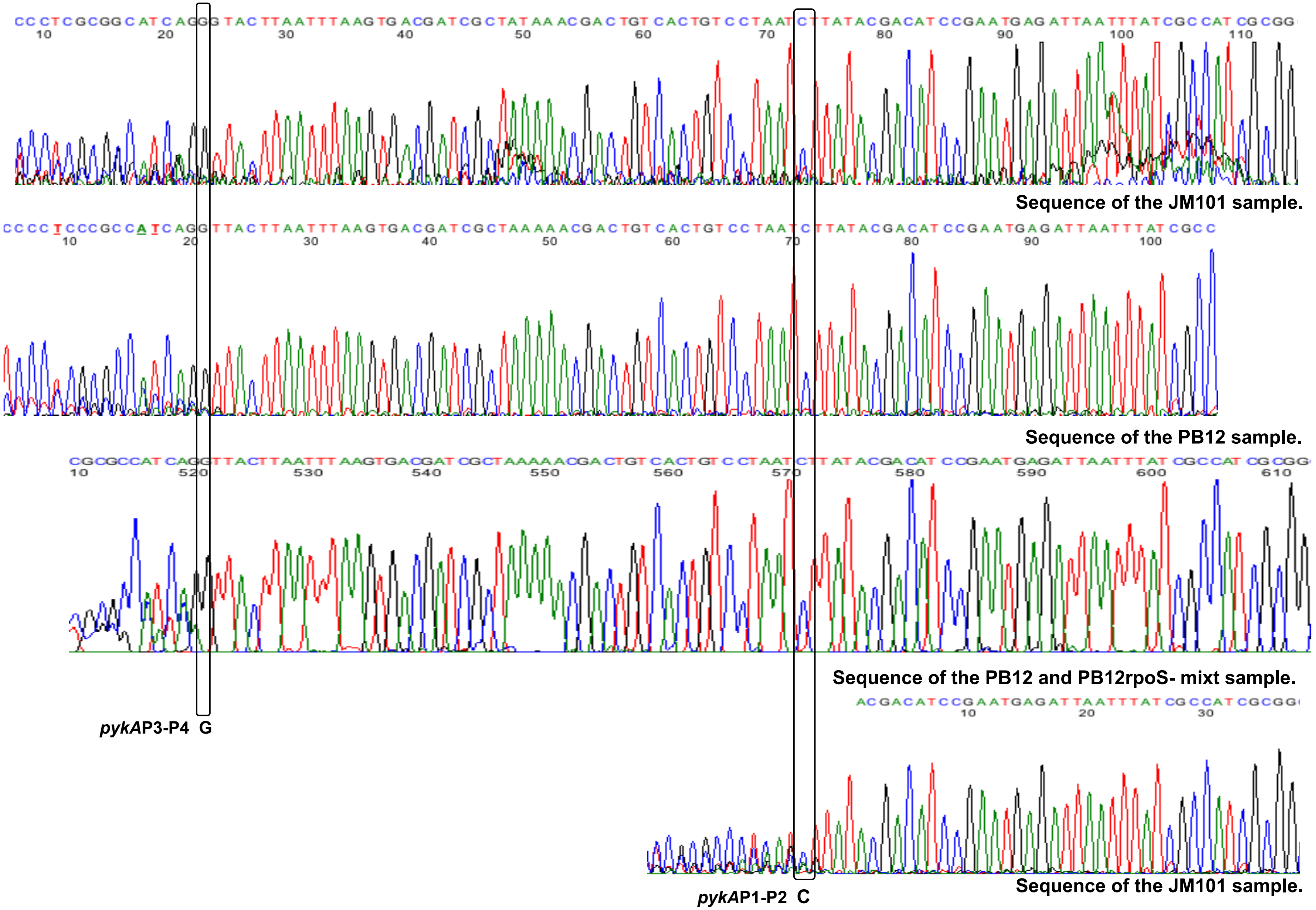

j-1 *pdhR* Transcriptional regulator of the Pyruvate dehydrogenase

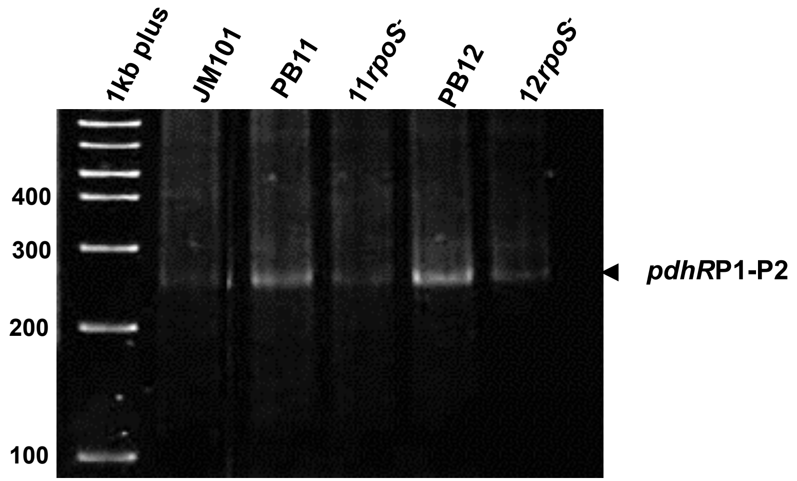

The detected TSS of the *pdhR* gene is the same as the one previously reported (51,52).

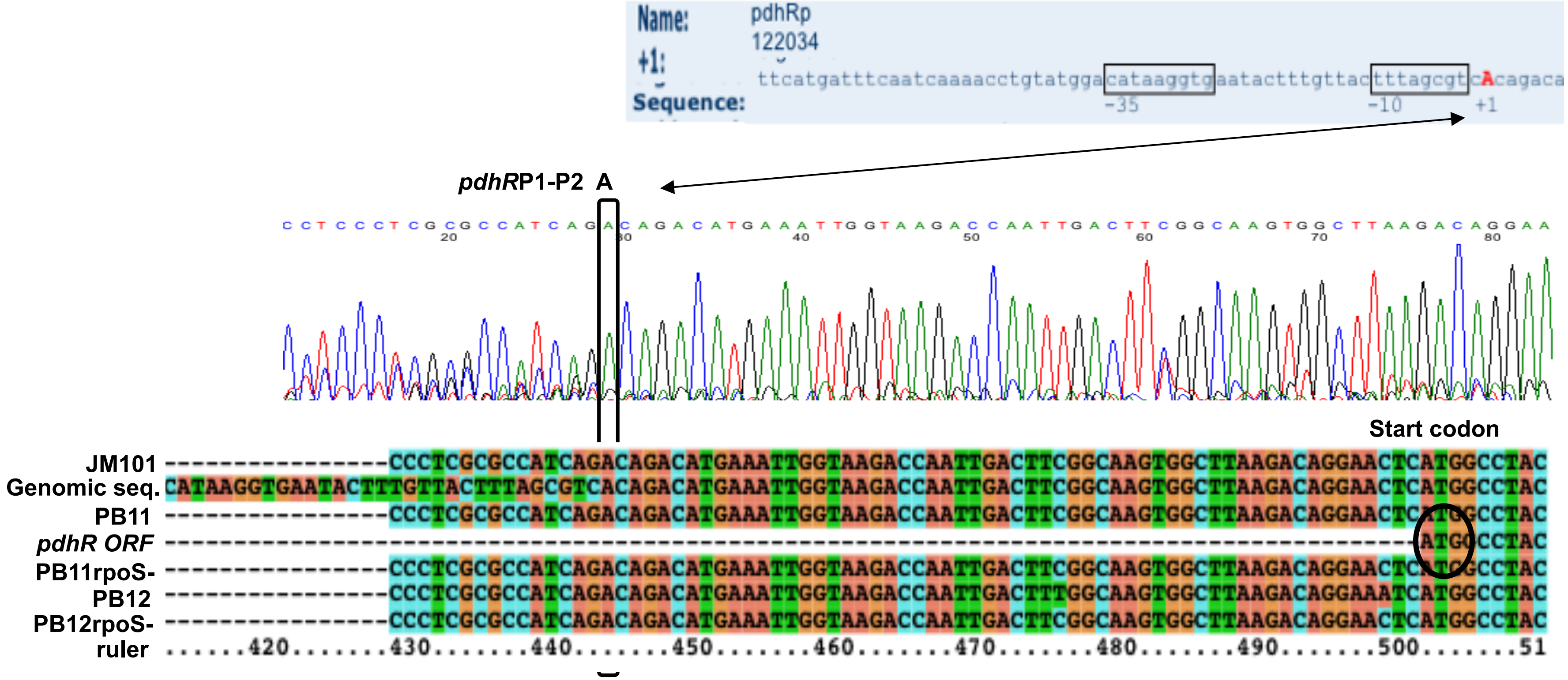

j-1 *pdhR* Transcriptional regulator of the Pyruvate dehydrogenase

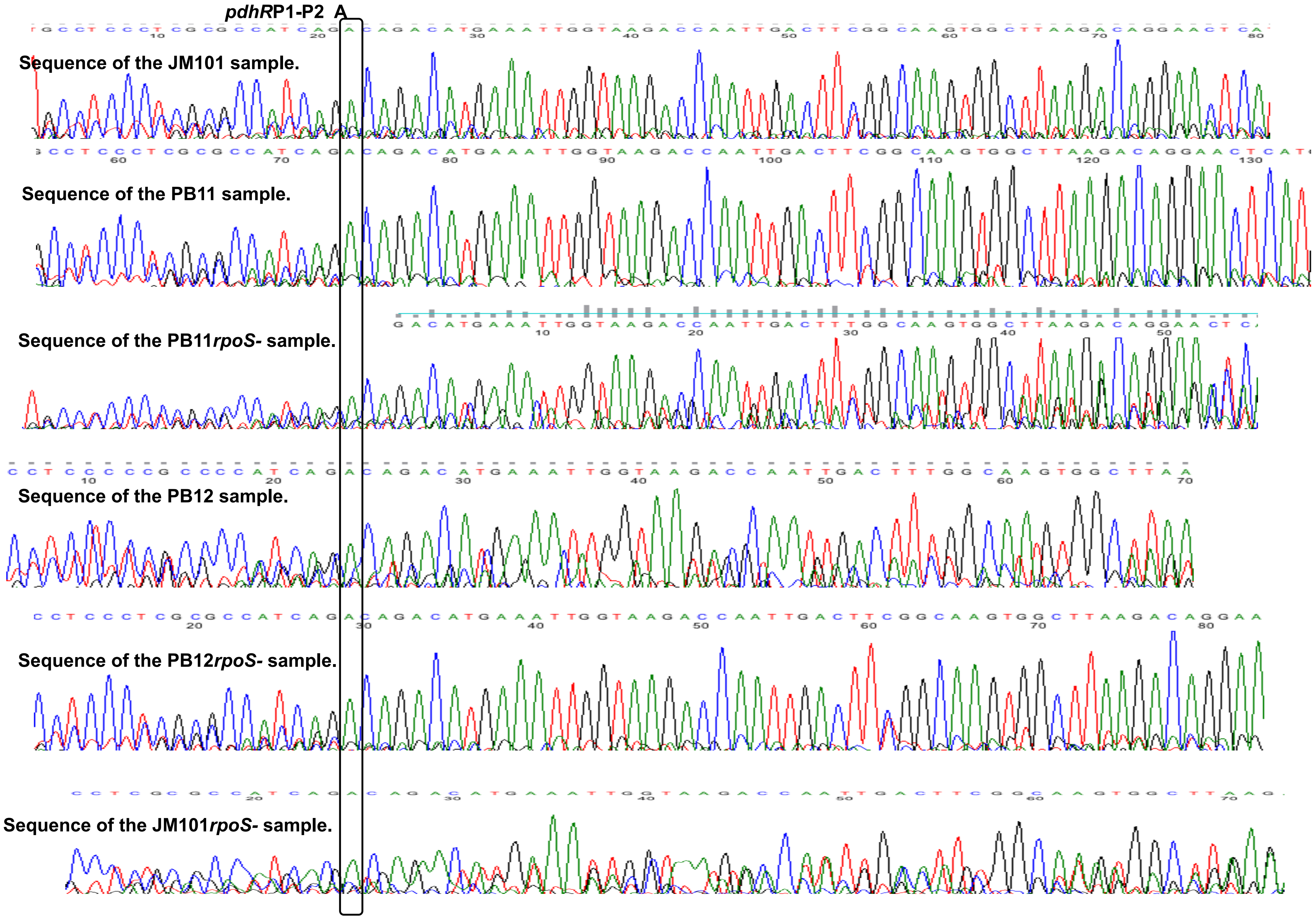

j-2 *aceE* Pyruvate dehydrogenase E1p subunit

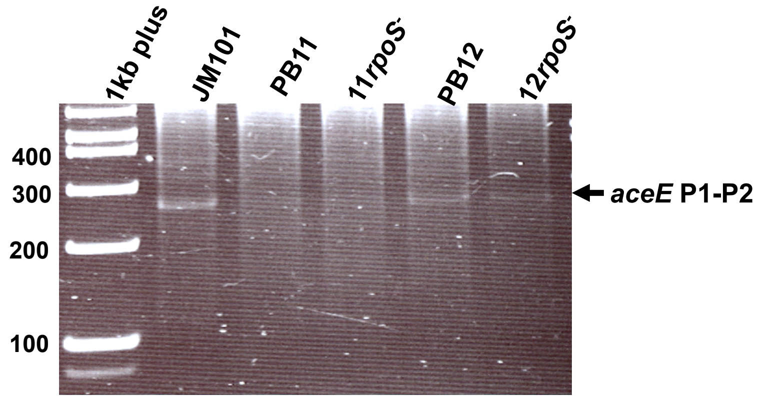

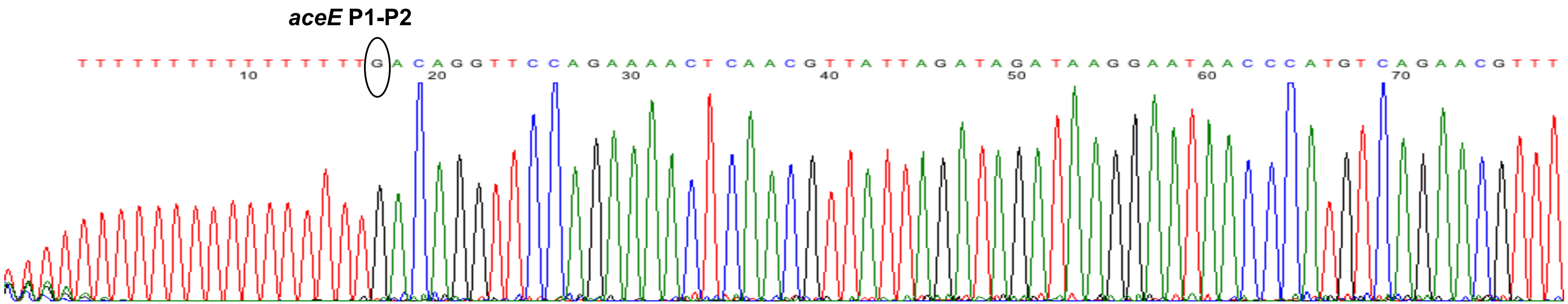

Sequence of the JM101 sample.

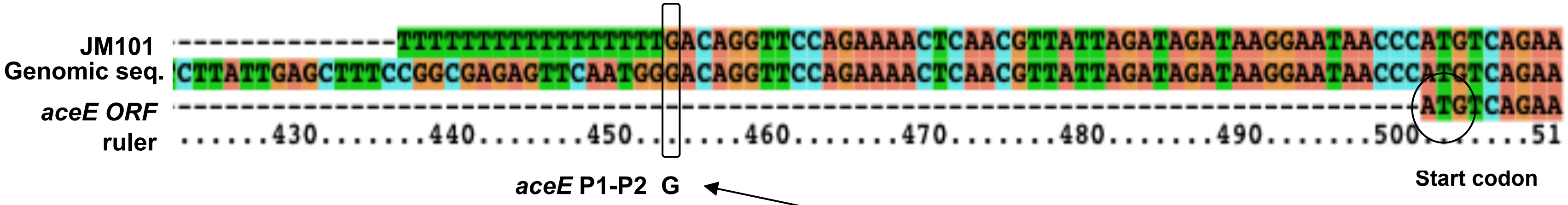

**Name:**	aceEp
**+1:**	122970
**Sequence:**	aagcgcggcaactaaacgtagaacctgtcttattgagctttccggcgagagttcaatgggAcaggttccagaaaactcaac

The detected TSS of the *aceE* gene is different from a previously reported by one nucleotide (50,51).

k. *poxB* Pyruvate oxidase

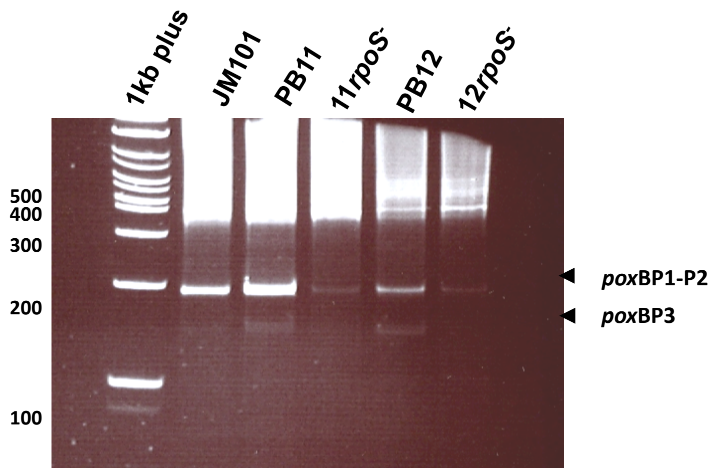

The detected TSS of the *poxB* gene is the same as the one previously reported (57,58).

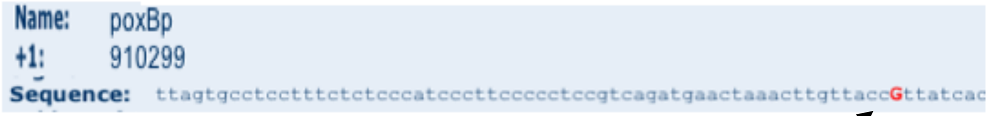

two methodologies

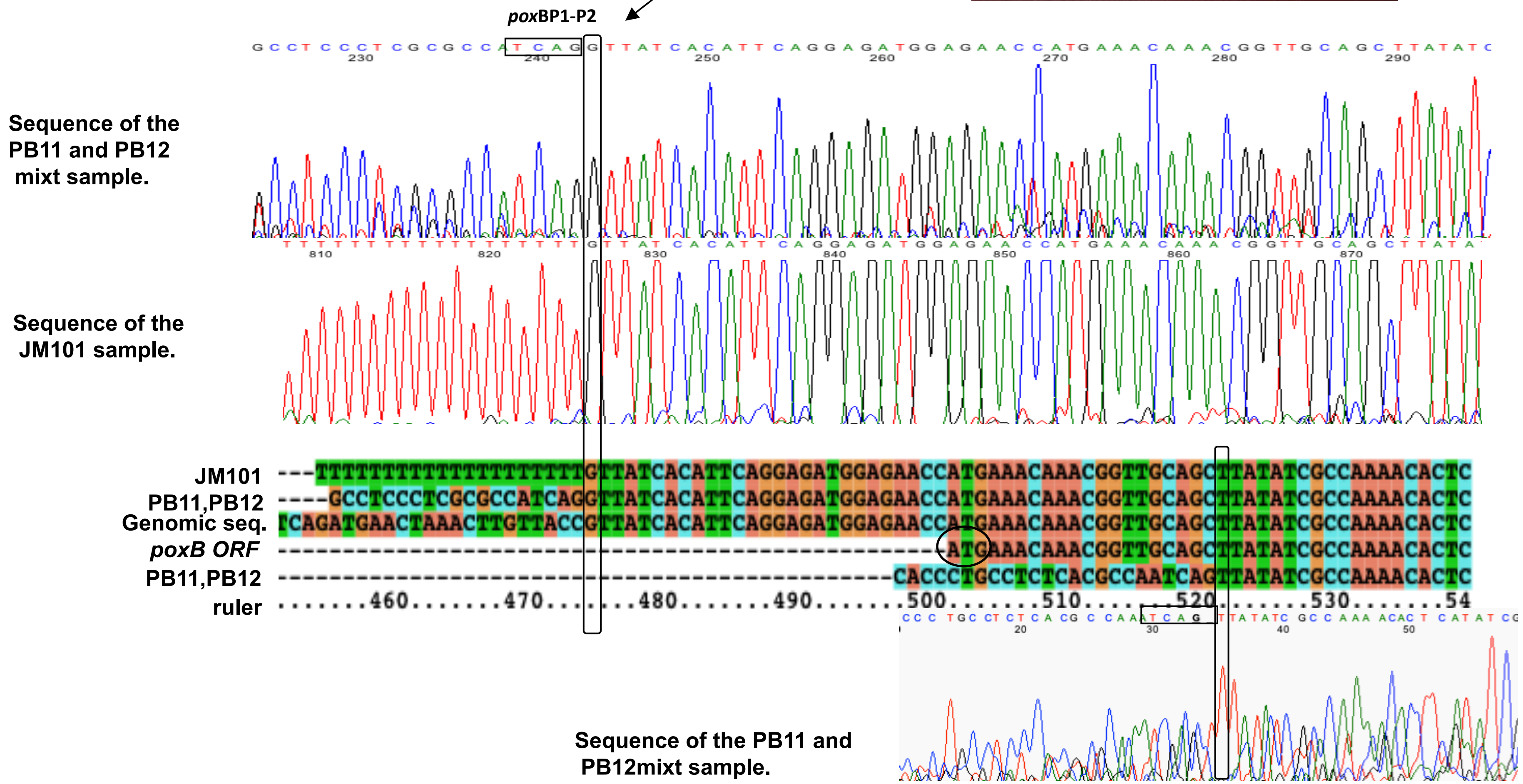

I. *acs* Acetyl-CoA synthase

The detected TSS of the *acs* gene is the same as the one previously reported (60,61).

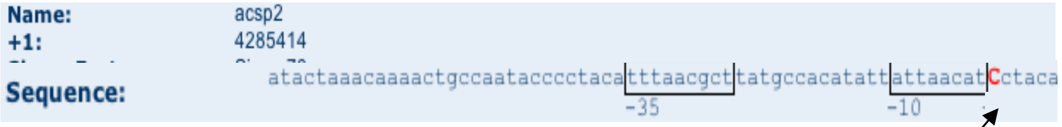

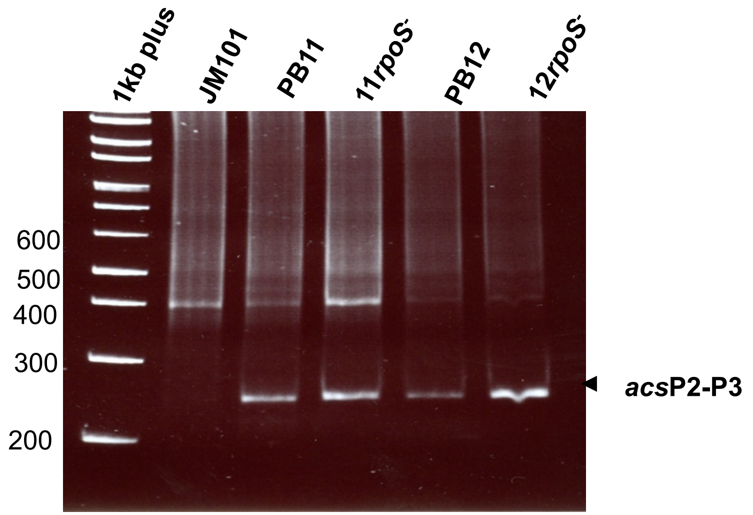

Two different methodologies were Utilized for the detection of the TSS.

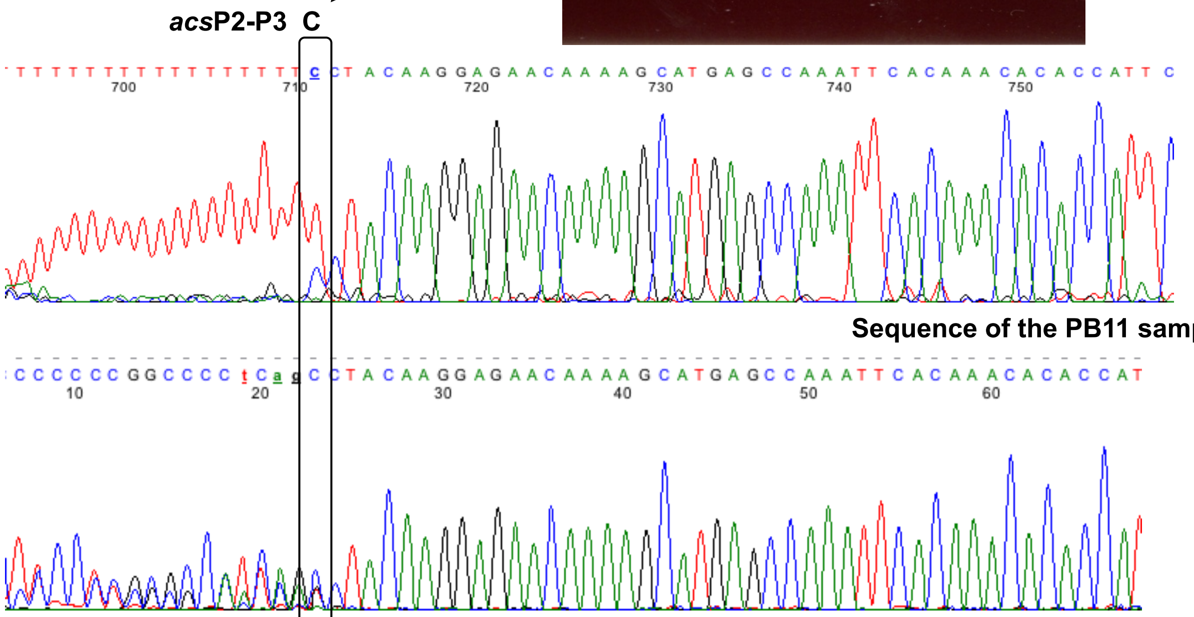

Sequence of the PB11 sample.

Sequence of the PB12 sample.

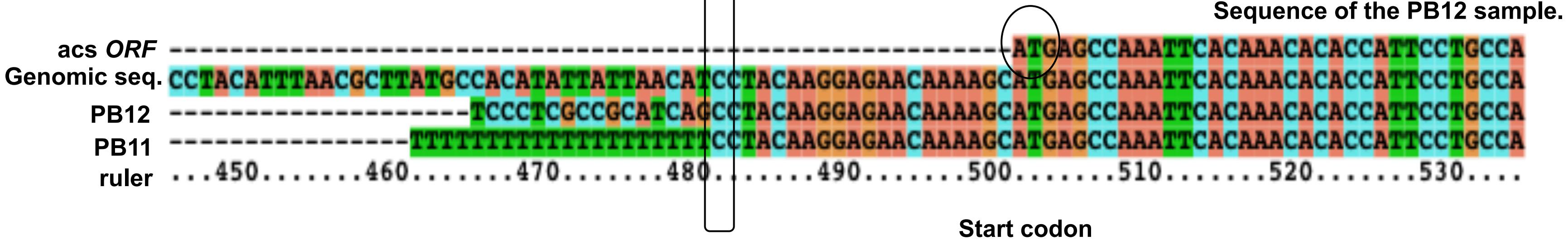

Start codon
