# Supplementary material for: Transcription Analysis of Central Metabolism Genes in Escherichia coli. Possible Roles of σ38 in Their Expression, as a Response to Carbon Limitation
Source: PLoS One. 2009 Oct 19;4(10):e7466. doi: 10.1371/journal.pone.0007466 (PMC2759082; doi:10.1371/journal.pone.0007466)
Supplement: Figure S2 — Detailed experimental strategy and complete set of results of in vitro transcription with purified E-sigma38 or E-sigma70. a; schematic representation of the promoter and terminator regions of the pSR plasmid used for in vitro transcription. This figure presents the way DNA fragments carrying each specific promoter region were cloned in plasmid pSR as EcoRI-HindIII fragments. As an example the nucleotide sequence of the EcoRI-HindIII fragment carrying the poxB promoter region, inserted into pSR, is presented. This vector has a rho-independent terminator, depicted in its stem and loop structure, such that the transcripts terminate at 88 nucleotides from the HindIII site marked by an asterisk. In the case of the poxB gene an RNA transcript of 88 nucleotides plus the size of the transcript from the +1(G) to the HindIII site (62 nucleotides), resulted in a transcript of 150 nucleotides (see also figure S2h). b; nucleotide sequence of the pgi promoter region inserted into pSR. As shown, two independent in vitro transcription experiments were performed for this gene. Transcripts originating from both putative overlapping dual pgiP1-P2 promoters were detected. c; nucleotide sequence of the pfkA promoter region inserted into pSR. As shown, two independent in vitro transcription experiments were performed for this gene. Transcripts originating from the putative overlapping dual pfkAP1-P2 promoters were detected. d; nucleotide sequence of the gapA promoter region inserted into pSR. As shown, an in vitro transcription experiment was performed for this gene. Transcripts originating from putative overlapping gapAP1-P1a dual promoters were detected. e; nucleotide sequence of the pykF promoter region inserted into pSR. As shown, an in vitro transcription experiment was performed for this gene. Transcripts originating from pykFP4-P5 and pykFP1-P2 putative dual promoters were detected. f; nucleotide sequence of the pykA promoter region inserted into pSR. As shown, two independent in [file pone.0007466.s002.pdf]

Figure S2

a. The nucleotide sequence of the *EcoR*I-*Hind*III segment carrying the promoter region of the *poxB* gene cloned in plasmid pSR, as well as other relevant DNA regions of this plasmid,are presented, as an example for the production of promoter gene specific fragments.

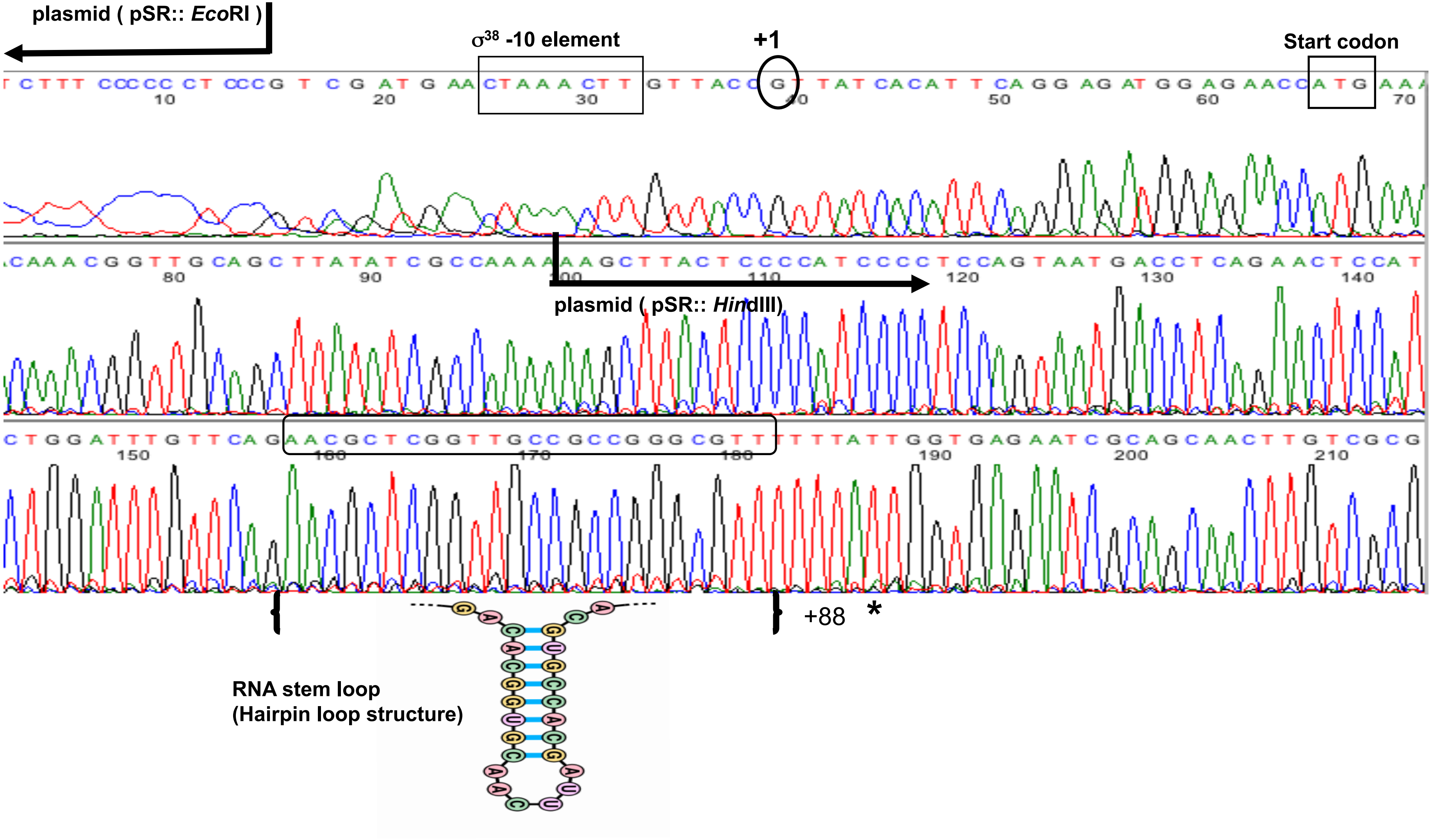

b. ***pgi*** **Glucosephosphate isomerase**

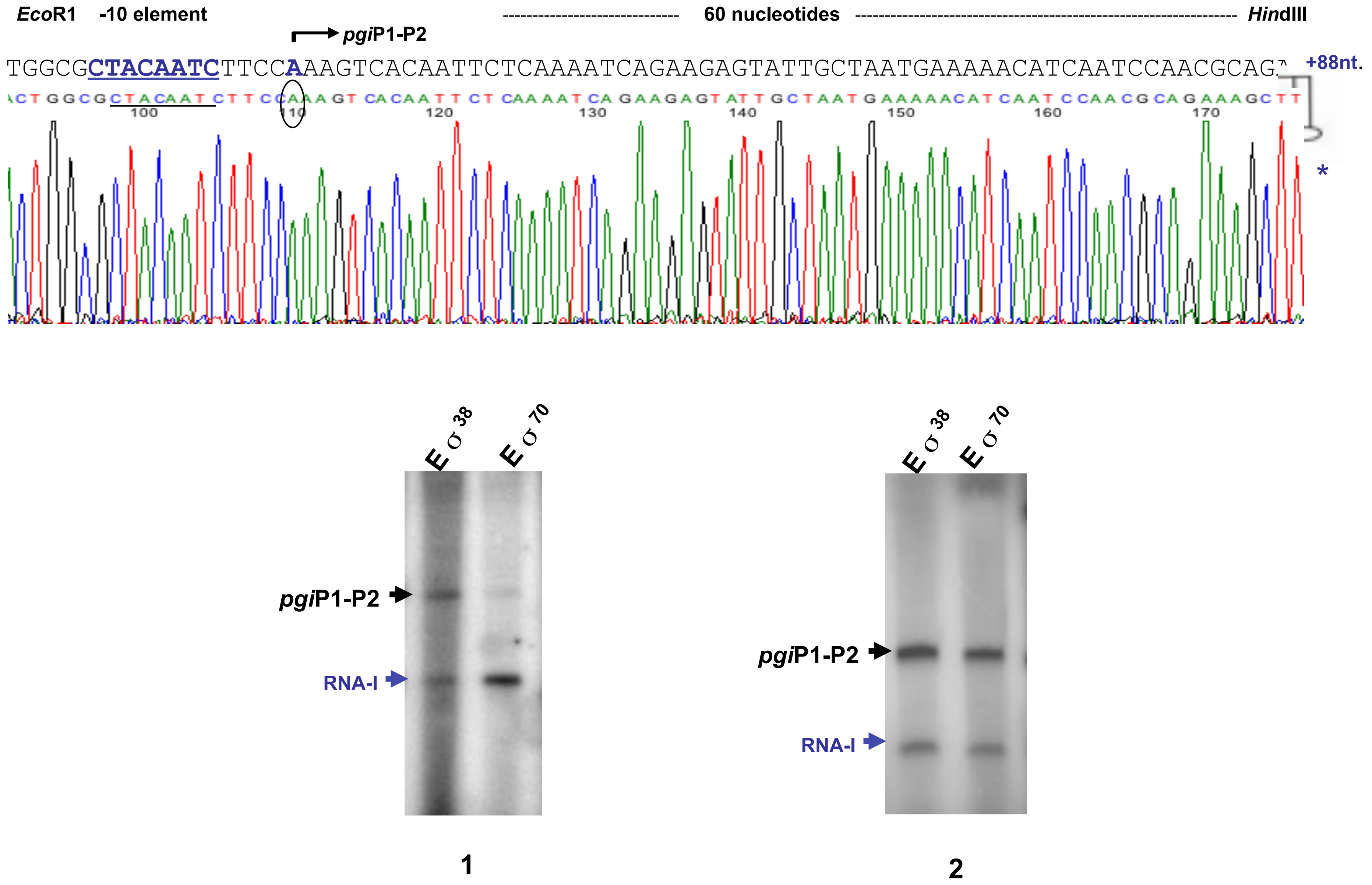

c. *pfkA* 6-phosphofructokinase I

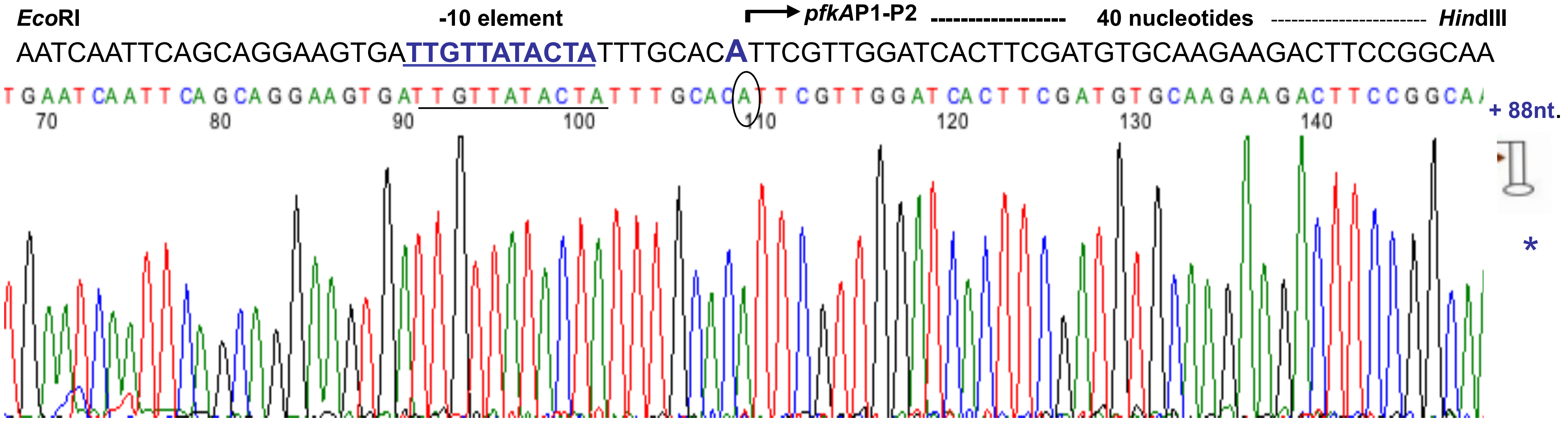

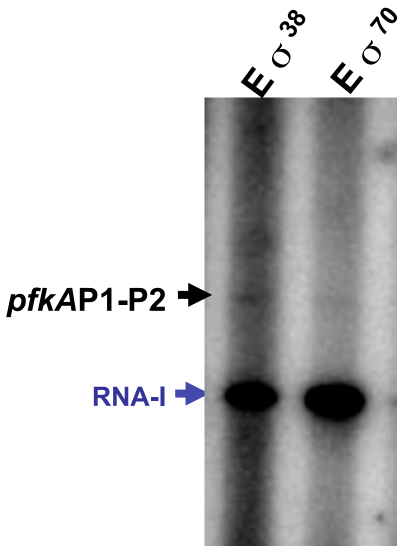

1

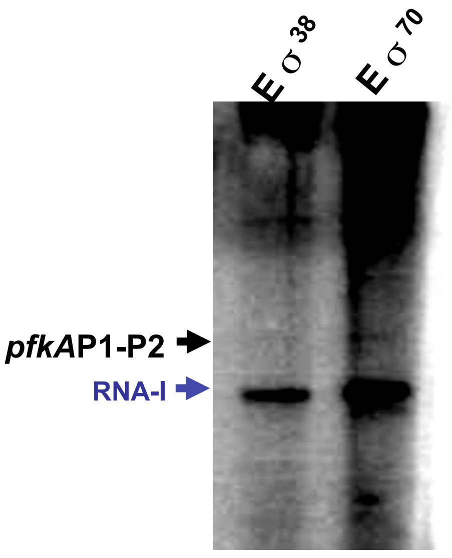

2

d. *gapA* Glyceraldehyde 3-phosphate dehydrogenase A

***Eco*RI** **-10 element** **→ *gapA*P1-Pia** **------------------- 62 nucleotides ----------------------- *Hind*III**

GCTGCGTAAGGTTTTT**<u>GTAATTTT</u>**ACAGGC**A**ACCTTTTATTCACTAACAAATAGCTGGTGGAATATAT

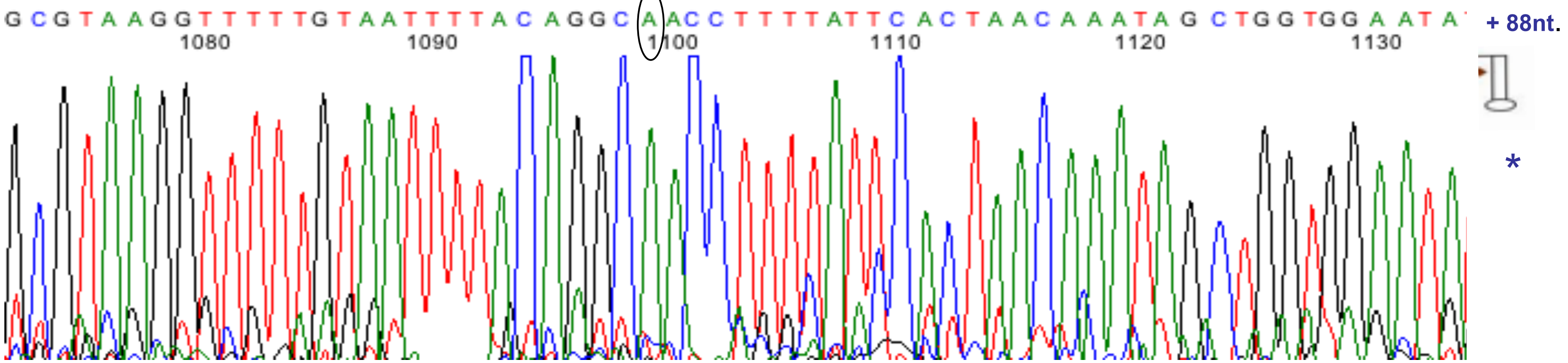

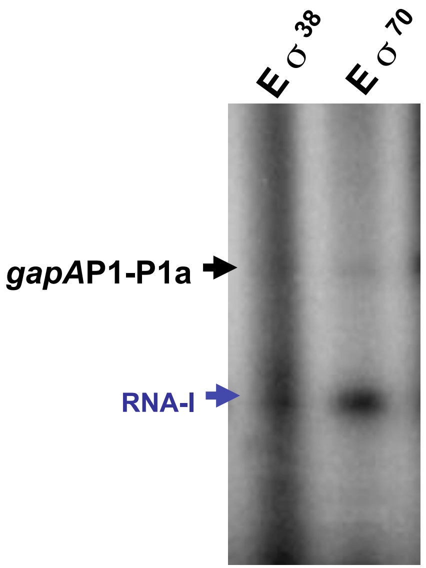

1

e.- *pykF* Pyruvate kinase F

***Eco*RI** **-10 element** **→ *pykF*P4-P5** ---------------------------------- **127 nucleotides** ------------------------------------

ACAAGCACACATTCCTCAC**<u>CTATCCTT</u>**AGAGC**G**AGGCACCACCACTTTC**G**TAATACCGGATTCGCTTTCCGGCAGTGC

→ *pykF*P1-P2 -- **62 nucleotides** -- ***Hin*dII**

GC**<u>CCAGAAAG</u>**CAAGTTT**C**TCCCATCCTTCTCAACTTAAAGACTAAGACTGTCATGAAAAAGACCAAAATTGTTTGCAC

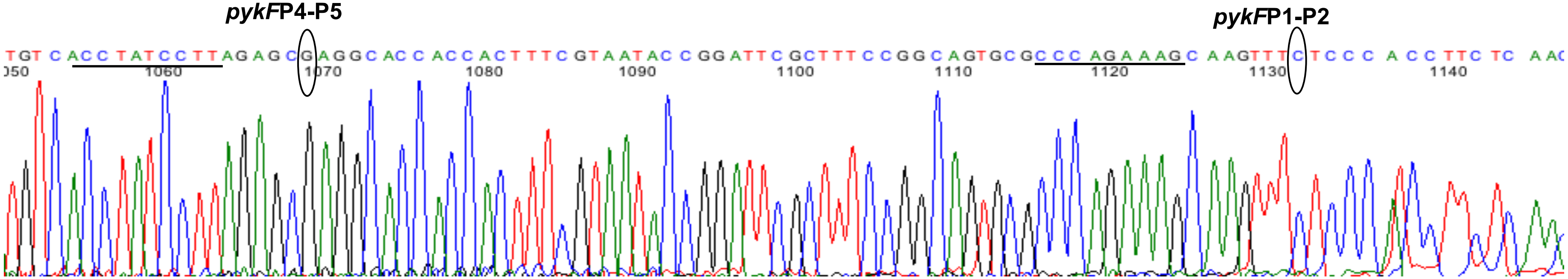

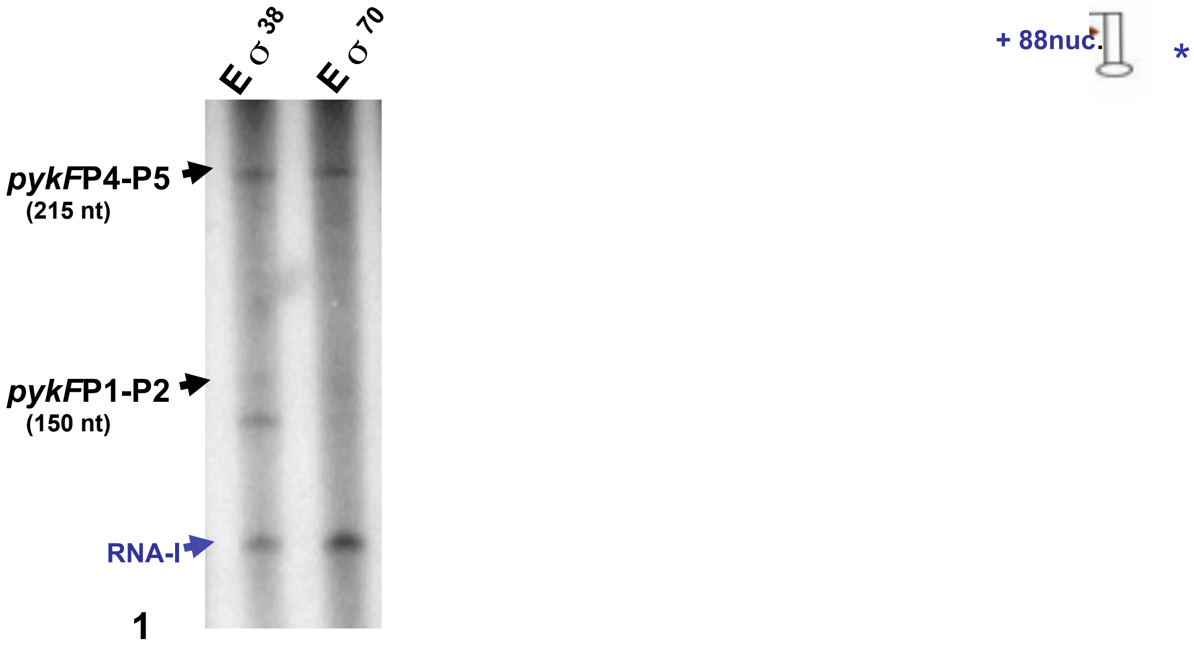

f. *pykA* Pyruvate kinase A

Eco*RI** **-10 element** **→ *pykA*P3-P4** ---------------------------- **111 nucleotides** -----------------------------Hind*III**

GCGCTGAAGGAAT**CGCGTTTTGATA**AGCA**G**TTACTTAATTTAAGTGACGATCGCTAAAAACGACTGTCA

→ *pykA*P1-P2 ---**62**--***Hind*III**

CTGTCCTAAT**C**TTATACGACATCCGAATGAGATTAATTTATCGCCATCGCGGCGTTATTTCATTCGGATTT

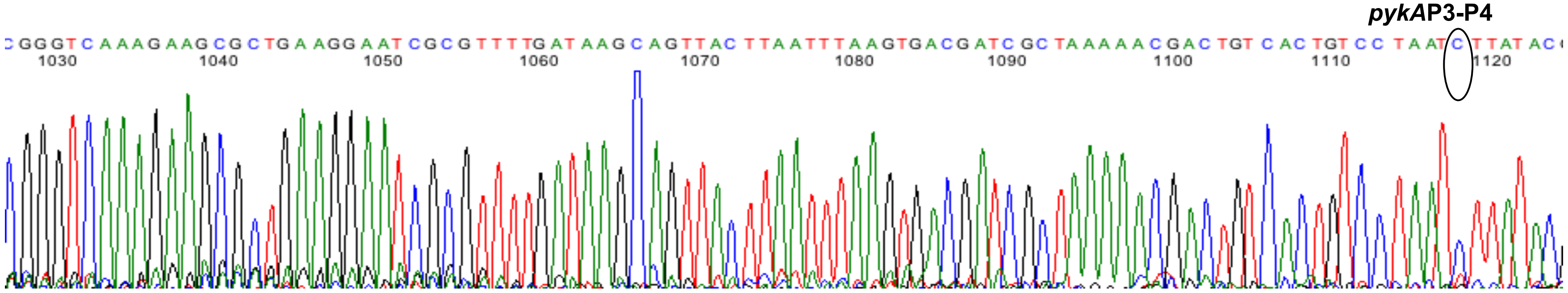

As shown, two independent experiments were performed for this gene.

+ 88nuc. *

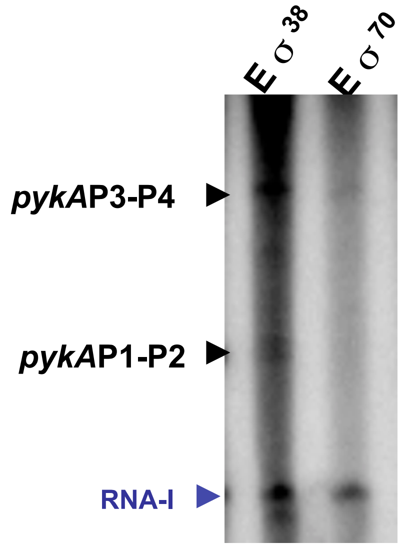

1

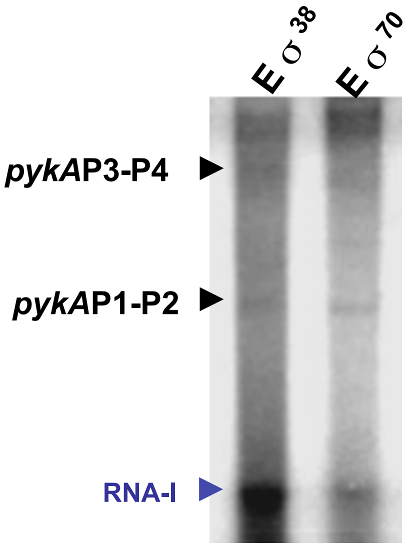

2

g. *pdhR* Transcriptional regulator of the pyruvate dehydrogenase

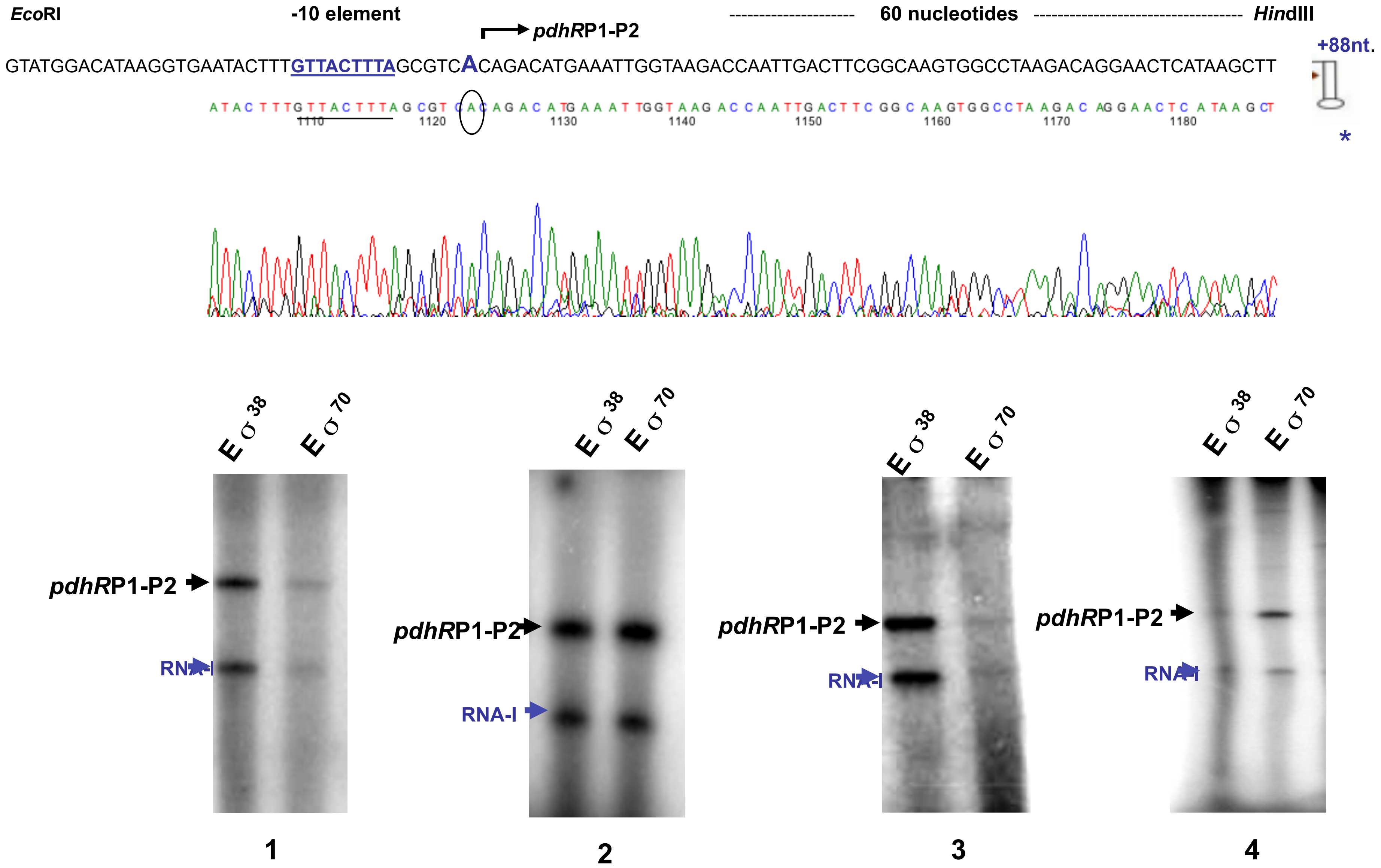

h. *poxB* Pyruvate oxidase

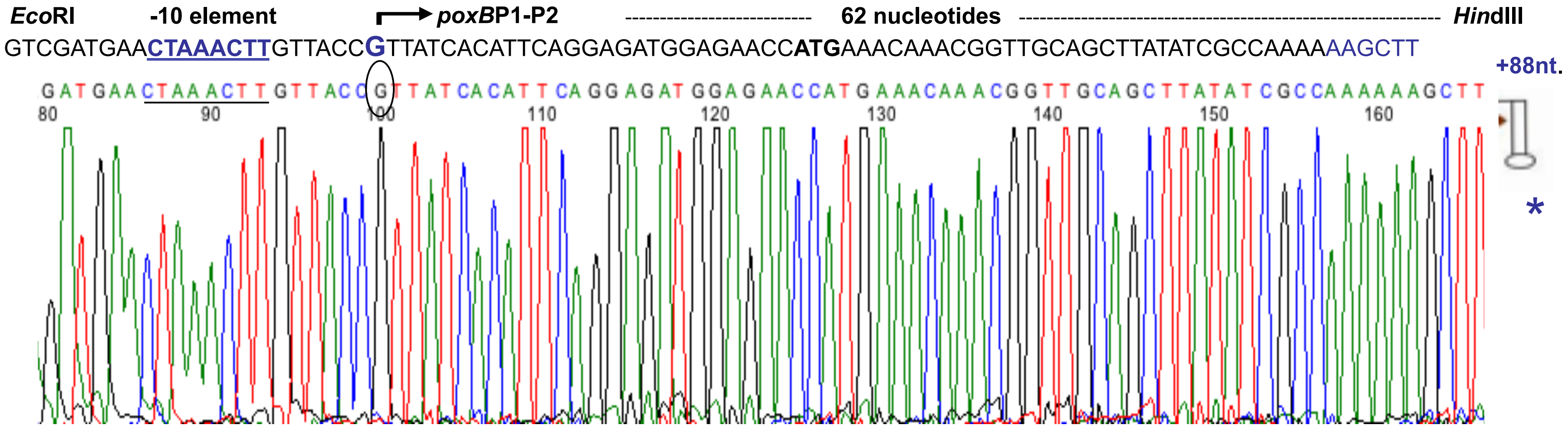

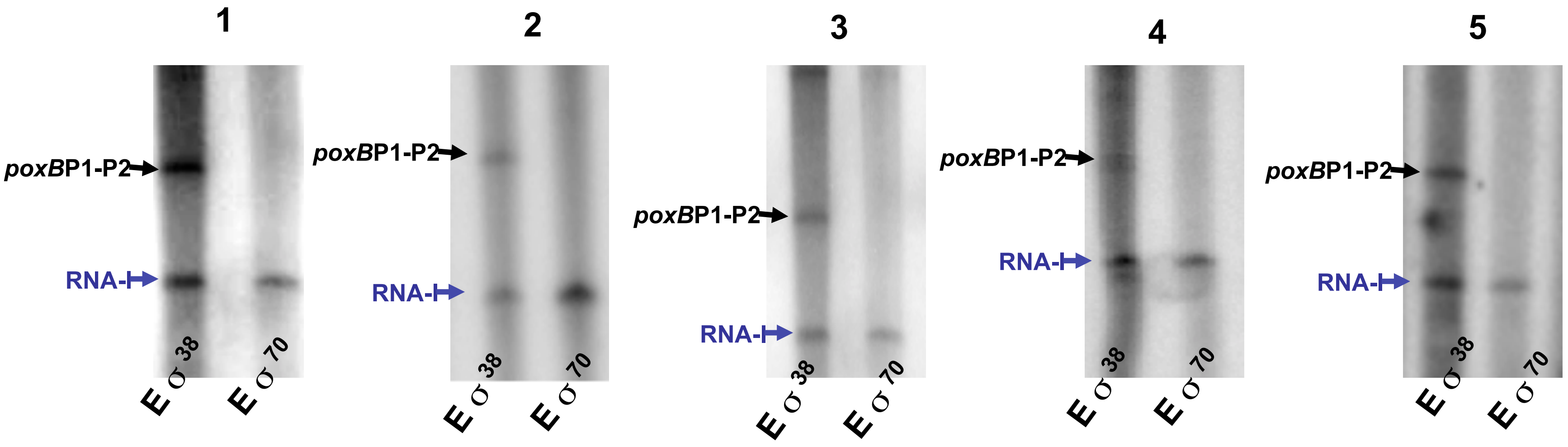

i. *acs* Acetyl-CoA synthase

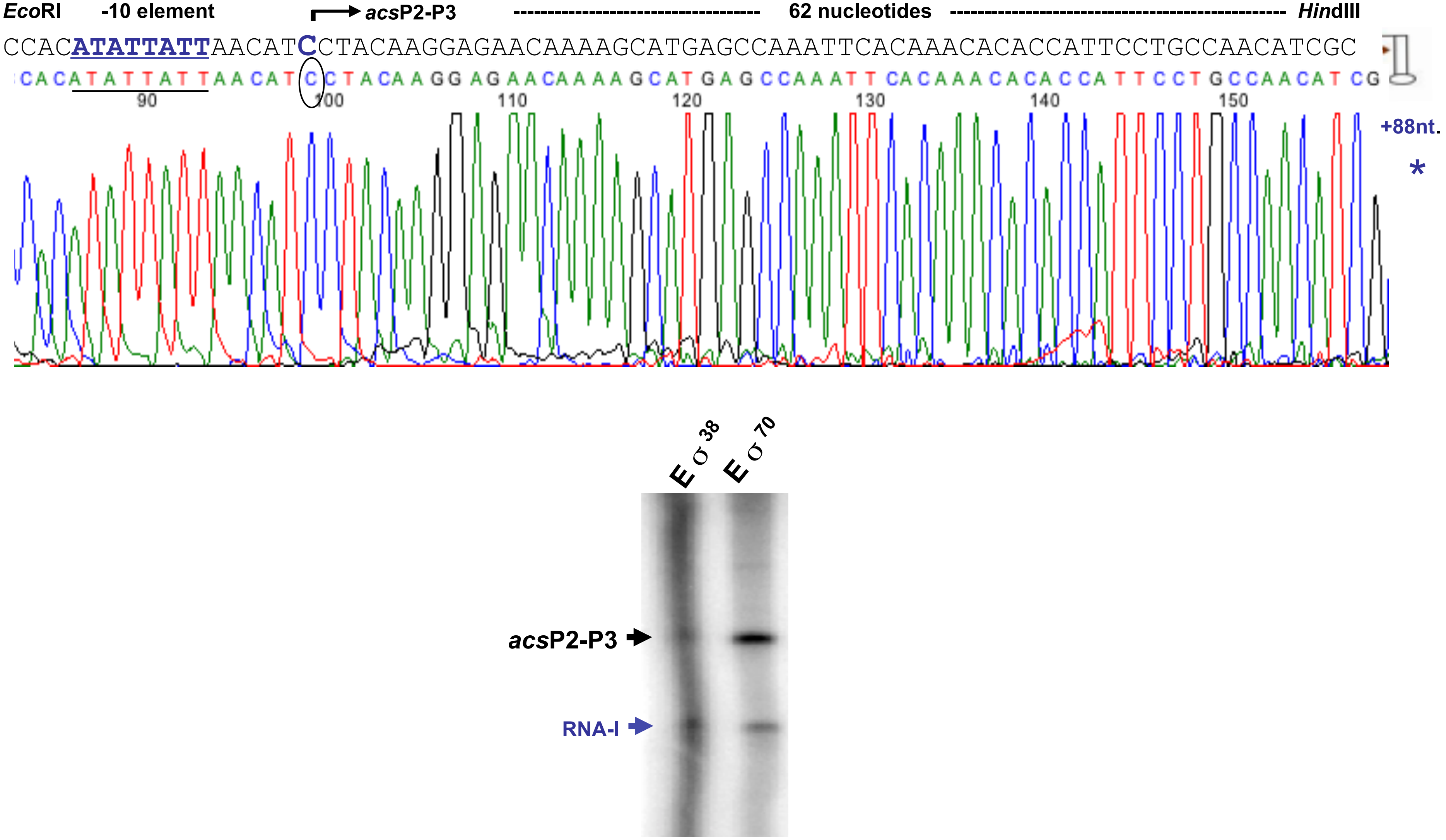

1
