# Supplementary material for: Transcription Analysis of Central Metabolism Genes in Escherichia coli. Possible Roles of σ38 in Their Expression, as a Response to Carbon Limitation
Source: PLoS One. 2009 Oct 19;4(10):e7466. doi: 10.1371/journal.pone.0007466 (PMC2759082; doi:10.1371/journal.pone.0007466)
Supplement: Figure S3 — The strategy for the construction of DNA fusions carrying the “closest to the ATG” promoters of selected glycolytic genes with a reported cat gene that confers chloramphenicol resistance (Cmr) is presented. Chromosomal DNA regions were constructed carrying these promoters of the selected genes together with the first 20 bp of the structural gene. Nucleotide sequences of the oligonucleotides that were utilized for amplifying the selected specific DNA fragments are presented in table S1. Each experiment utilized one forward (Fw) and one reverse (Rv) fragments to produce the PCR amplified products. Selected amplified DNA fragments were cloned into the SmaI site of plasmid pKK238; this site is in front of the cat reporter gene of this plasmid. The sizes of recombinant molecules in the inserts, were assayed by digesting plasmids with PstI. As can be seen in the gel, we were able to clone all the “promoter or dual promoters closest to the ATG initiation codon” of the analyzed genes. DNA fragments carrying these promoters: pgiP1-P2 (PCR size of 171 bp and 1127 bp after PstI digestion, respectively), pfkAP3-P4 (140 and 1096), tpiAP1-P2 (117 and 1073), enoP4-P5 (105 and 1064), and pgkP2 (108 and 1064), are presented in the figure and in the gel. The agarose gel exhibits PstI digested DNA from the different recombination molecules, including the vector digested with the same enzyme that generates a band of 953 bp (column B). All cloned DNA fragments had higher molecular weights than 953 bp after PstI digestion, indicating that the insert is present. The gel also includes molecular weight markers (column A). The nucleotide sequences of the inserted fragments confirmed the existence of the cloned DNA fragments (data not shown). (1.49 MB PPT) [file pone.0007466.s003.ppt]

## Slide 1
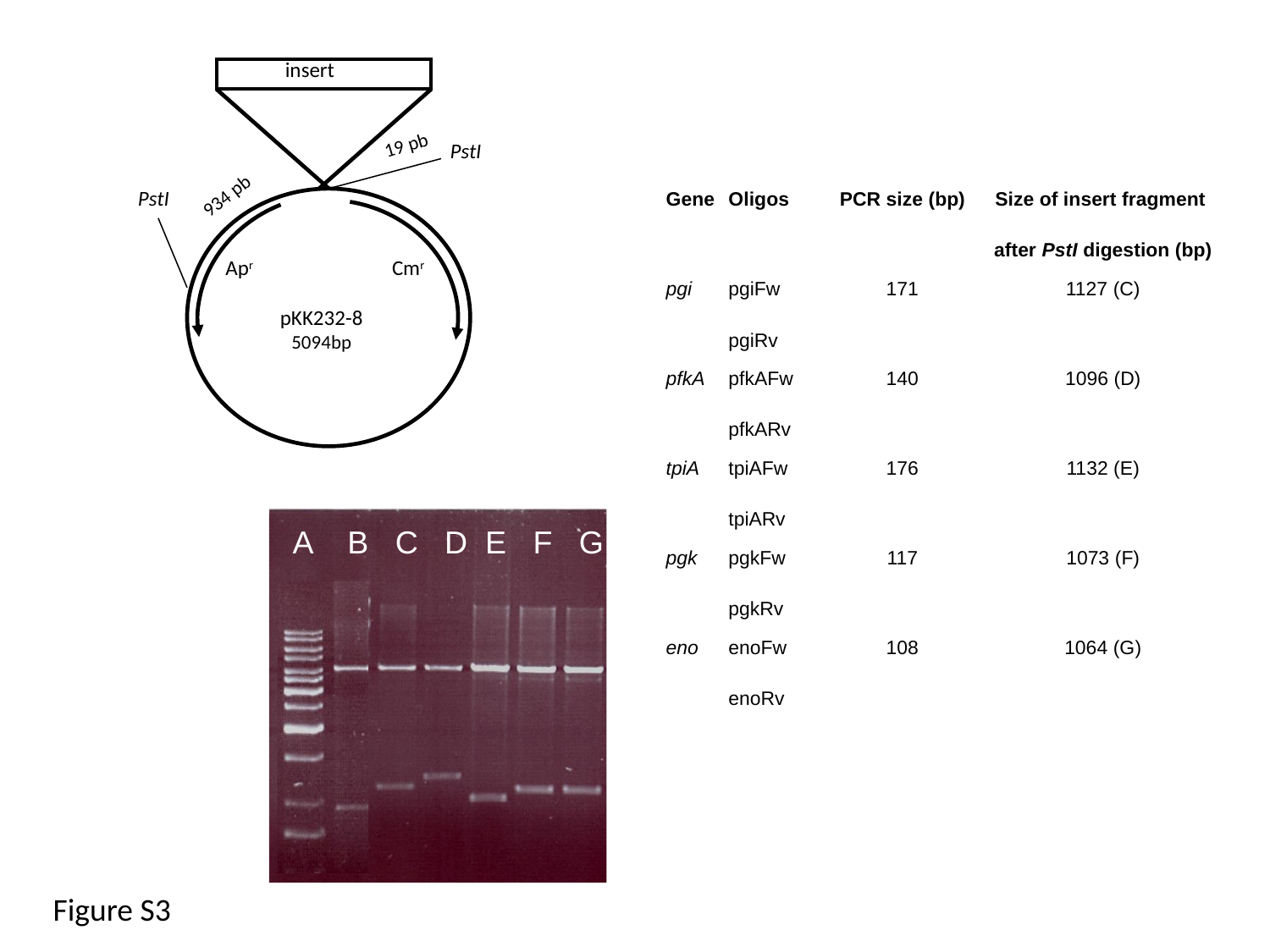

insert
19 pb
PstI
934 pb
PstI
Apr
Cmr
pKK232-8
5094bp
| Gene | Oligos | PCR size (bp) | Size of insert fragment |
| --- | --- | --- | --- |
| | | | after PstI digestion (bp) |
| pgi | pgiFw | 171 | 1127 (C) |
| | pgiRv | | |
| pfkA | pfkAFw | 140 | 1096 (D) |
| | pfkARv | | |
| tpiA | tpiAFw | 176 | 1132 (E) |
| | tpiARv | | |
| pgk | pgkFw | 117 | 1073 (F) |
| | pgkRv | | |
| eno | enoFw | 108 | 1064 (G) |
| | enoRv | | |
A B C D E F G
Figure S3
